# Modeling Platelet P2Y_1_/_12_ Pathway to Integrin Activation

**Published:** 2024-10-16

**Authors:** Keshav B. Patel, Wolfgang Bergmeier, Aaron L. Fogelson

**Affiliations:** 1 Department of Mathematics, University of Utah, Salt Lake City, UT, 84112; 2 Department of Biochemistry and Biophysics, University of North Carolina at Chapel Hill, Chapel Hill, NC, 27599; 3 Blood Research Center, University of North Carolina at Chapel Hill, Chapel Hill, NC, 27599; 4 Department of Bioengineering, University of Utah, Salt Lake City, UT, 84112

## Abstract

Through experimental studies, many details of the pathway of integrin αIIVβ3 activation by ADP during the platelet aggregation process have been mapped out. ADP binds to two separate G protein coupled receptors on platelet surfaces, leading to alterations in the regulation of the small GTPase RAP1. We seek to (1) gain insights into the relative contributions of both pathways to RAP1-mediated integrin activation and to (2) predict cell behavior in response to a continuous range of external agonist concentrations. To this end, we develop a dynamical systems model detailing the action of each protein in the two pathways up to the regulation of RAP1. We perform a parameter estimation using flow cytometry data to determine a number of unknown rate constants. We then validate with already published data; in particular, the model confirmed the effect of impaired P2Y_1_ receptor desensitization or reduced RASA3 expression on RAP1 activation. We then predict the effect of protein expression levels on integrin activation and show that components of the P2Y_12_ pathway are critical to the regulation of integrin. This model aids in our understanding of interindividual variability in platelet response to ADP and therapeutic P2Y_12_ inhibition. It also provides a more detailed view of platelet activation in the ongoing mathematical study of platelet aggregation.

## INTRODUCTION

Platelets are the primary cellular component in arterial blood clots, supporting the coagulation response and recruitment of other cells/proteins to a growing thrombus. They perform a variety of processes that aid in clot growth and stabilization, such as releasing agonists into the environment to recruit other platelets, changing shape through cytoskeletal remodeling to increase surface area for reactions, and activating integrins on their surfaces for binding crosslinking proteins. For the purposes of this work, we will use the words “integrin activation” to refer to the conversion of integrin $\alpha_{\text{IIV}}\beta_{3}$ from a low fibrinogen affinity state to a high-affinity state.

Several molecules serve as receptor ligands that lead to integrin activation (1). Most ligands, like thrombin or collagen, initiate pathways that leads to irreversible activation of the small protein RAP1, which complexes with the integrin on its cytosolic end and leads to a conformation change on the extracellular end. Signaling pathways for other receptor-ligand bindings have been extensively studied by both experimentalists and modelers. Dunster et al. modeled the activation signals associated with collagen binding the GPVI receptor (2) and Lenoci et al. and Sveshnikova et al. modeled the irreversible activation of small GAP protein RAP1 by the PAR1 receptor (3, 4).

ADP binds to two different receptors, the G-protein coupled receptors (GPCR) P2Y_1_ and P2Y_12_, and is unique in that it leads to a partial, transient activation of integrin that lasts only a few minutes. Experimental work has uncovered much about the pathways that lead to the activation of integrin. Activation of P2Y_1_ induces activation of RAP1 via the calcium-dependent RAP-GEF, CalDAG-GEFI (CDGI), while activation of P2Y_12_ induces RAP1 activation through the inhibition of the RAP-GAP, RASA3 (5). The regulation of RAP1 is vital to maintaining platelet quiescence in the absence of clot formation, and therefore understanding the individual contribution of CalDAG-GEFI and RASA3 in integrin activation is of interest. Other models have been developed to study platelet activation and aggregation mediated by ADP (6), but to the authors’ knowledge, no detailed signaling pathways for ADP dependent activation have been developed.

In this work, we convert the collected information on both pathways to a system of ordinary differential equations (ODEs) that can be solved numerically to simulate a single platelet’s response to a given amount of P2Y_1_ and/or P2Y_12_ agonist. We use experimental data to estimate unknown parameters and validate the model against recently published data. We then explore how a simulated platelet responds to agonists under a change to the expression level of specified proteins. Unlike in experimental settings, we are able to tune expression with much finer precision to gain a more quantitative measure of sensitivity. We begin by describing the chemical signaling pathways upon platelet GPCR binding to ADP.

### Biological Background

**Figure 1:**
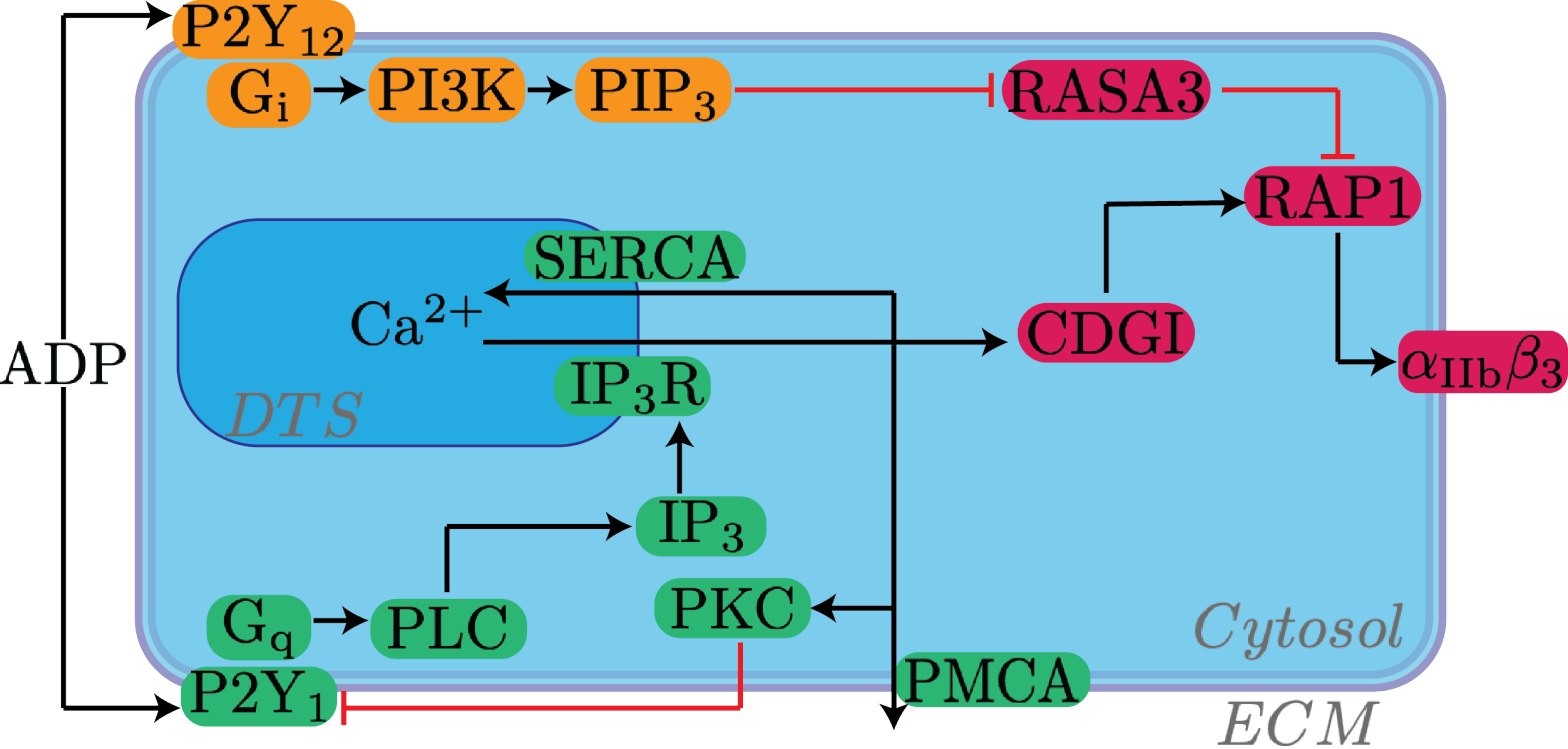
Schematic of intracellular pathways involved in integrin activation. ADP binds to G protein Coupled Receptors P2Y_1_ and P2Y_12_ on platelet surfaces, each initiating its own signaling pathway and both contributing to a change in in RAP1-GTP concentration.

Figure 1 shows schematically how the P2Y_1_ and P2Y_12_ receptors are activated by ADP and, through different pathways, cause changes to both the activator and inhibitor of RAP1.

The extracellular domains of the platelet P2Y_1_ and P2Y_12_ receptors bind to ADP in the blood plasma. Upon binding to ADP, these receptors act as GEF enzymes on the membrane-associated G proteins G_q_ and G_i_, respectively. The G proteins then unbind from the receptor and bind to membrane-associated enzymes PLC and PI3K, respectively. Both PLC and PI3K use the lipid PIP_2_ as substrate: PLC converts PIP_2_ to the membrane-associated molecule DAG and the cytosolic molecule IP_3_, and PI3K converts PIP_2_ to membrane-associated PIP_3_. These inositol lipids are part of a large cycle of formation and degradation. IP_3_ binds to IP_3_ receptors (IP_3_R) embedded in the dense tubular system (DTS) membrane and triggers the release of calcium ions from the DTS into the cytosolic space. Platelets contain other intracellular calcium stores, e.g., the mitochondrial and acidic stores (7), which impact calcium levels on a significantly longer timescale and therefore are ignored for this study. The chemically-gated IP_3_R channel is a four-subunit protein; each subunit contains an activating binding site for IP_3_, an activating binding site for calcium, and a slower inactivating binding site for calcium (8). Thus, the initial release of calcium from the DTS leads to a stage of positive feedback followed by negative feedback.

At rest, cytosolic calcium levels are maintained by exchanges with the plasma, in which calcium is assumed to have a concentration around 1 mM. The model incorporates the action of the PMCA pump, a passive leak current on the plasma membrane, and the action of the SERCA pump across the DTS membrane.

Calcium’s effect on integrin activation is mediated through its binding to the GEF enzyme CalDAG-GEFI. CalDAG-GEFI contains two EF domains which each binds to calcium with a dissociation constant $K_{\text{GEF},\text{M}}=80$ nM (9). Calcium-bound CalDAG-GEFI converts RAP1 from its inactive GDP-bound form to its active GTP-bound form. The active form of RAP1 complexes with a variety of proteins on the cytosolic tail of $\alpha_{\text{IIV}}\beta_{3}$, namely TALIN and KINDLIN3, to trigger integrin activation. RASA3 is a membrane-associated protein constitutively active in converting active RAP1 to its inactive GDP-bound form. RASA3 can be inactivated by interacting with PIP_3_, although the exact mechanism has not been determined (10). CalDAG-GEFI and RASA3 have nearly equal copy numbers in mouse platelets (approximately 30,000 plt^−1^) whereas RAP1 in its two major isoforms has a total copy number of approximately 200,000 plt^−1^ (11).

## MATERIALS AND METHODS

We used Mass Action or Michalis-Menten kinetics to describe each reaction in the P2Y_1_ and P2Y_12_ pathways. The concentration, denoted by [⋅], or surface density, denoted by ${\left[\cdot \right]}_{S}$, of each type of molecule in its various states was tracked using an ordinary differential equation (ODE). The overall model, therefore, comprised an extensive coupled system of ODEs that we must solve simultaneously, and we accomplished this using LLNL’s DLSODE solver package (12).

Here, we discuss the setup of the differential equation system. We used the model of Purvis et al. (13) as a starting point for our modeling; below, we discuss explicitly where we deviated and extended their model. The system was described by 82 differential equations; see the supplemental material for a complete list of the model’s equations. We then describe the statistical methods used to inform unknown model parameters. Finally, we describe the experimental methods used to generate the data against which we validate the model.

### Model components and numerical setup

#### ADP Receptors:

Following prior modeling work on G protein coupled receptors (14), we explicitly tracked the state changes of the P2Y_1_ and P2Y_12_ receptors as they bind to ADP and their specific G protein. Each binding, unbinding, and catalytic reaction is described using the Law of Mass Action. Receptors bound to an inactive G protein can activate it, regardless of whether ADP is also bound to the receptor. However, the presence of ADP significantly increases the receptor’s efficiency.

We made two notable changes to the model as implemented in Purvis et al. (13). First, this and other previous models include an “inactive” GPCR state converted into an “active” state through a reversible, unimolecular reaction. We noted through numerical simulations (not shown) that the concentration for each inactive state quickly reached an equilibrium value proportional to the concentration for the corresponding active state. Therefore, we performed a quasi-steady-state (QSS) reduction to reduce the number of explicit states.

Second, we included a PKC-dependent inactivated state for P2Y_1_. We assumed that ADP-bound P2Y_1_ can be inactivated via phosphorylation of an intracellular site and reactivated via dephosphorylation. PKC mediates the phosphorylation process. The kinetic parameters for this reaction were estimated in this study.

#### Second Messengers:

The model explicitly tracked the second messenger proteins PLC and PI3K in their inactive state and active (i.e., bound to G protein) state (15). Binding and unbinding of G proteins were tracked through Mass Action kinetics, with an additional irreversible hydrolysis term, in which the second messenger both converts the α subunit to a GDP bound form and releases it into the cytosol. Once activated, PLC and PI3K act upon the phosphoinositol species PIP_2_, converting it to IP_3_ and PIP_3_, respectively. These inositol species are recycled through a variety of states by various other phosphatases and kinases (16–23).

#### Calcium Dynamics:

The flux of calcium ions across passive elements (namely, the IP_3_R and PM leak) are governed by the Nernst Equation. For example, the rate at which calcium moves across the DTS membrane through conducting IP_3_R channels is given by:

(1) $$R_{\text{DTS}}=\frac{N_{{\text{IP}}_{3}\text{R}}}{4}P_{0}\gamma_{{\text{IP}}_{3}\text{R}}\frac{RT}{{\left(zF\right)}^{2}}\log\left(\frac{{\left[{\text{Ca}}^{2+}\right]}_{\text{dts}}}{{\left[{\text{Ca}}^{2+}\right]}_{\text{cyt}}}\right),$$

where $N_{{\text{IP}}_{3}\text{R}}$ is the copy number of ${\text{IP}}_{3}\text{R}$ subunits, $P_{0}$ is the probability a single channel is in a conducting state, γ is the conductance of the channel, R is the universal gas constant, T is the temperature, z is the charge of a calcium ion, F is Faraday’s constant, and ${\left[{\text{Ca}}^{2+}\right]}_{\text{dts}}$ and ${\left[{\text{Ca}}^{2+}\right]}_{\text{cyt}}$ are the free calcium concentrations in the DTS and cytosol, respectively. We model the IP3R channel using the six-state model given by Sneyd and Dufour (8) and use their formula for computing $P_{0}$, which is dependent on the fraction of receptors in the two conducting states.

Once calcium is released into the cytosol, it can bind to various pumps, enzymes, and buffers. We modeled both the SERCA and PMCA pumps using 6-state transport models (24), where two calcium ions at a time are transported across their respective membranes. We explicitly modeled PKC binding of calcium and DAG using Mass Action kinetics. As described above, PKC molecules bound to both calcium and DAG can inactivate the P2Y_1_ receptor. To match the experimental setup, we included the binding of calcium to a fluorescent experimental probe with a concentration of 5 μM and a dissociation constant of 100 nM. This probe was only included during the parameter estimation that relied on calcium data. In all other experiments, we excluded the probe.

#### GEF/GAP Enzymes:

We modeled both the binding of calcium with CalDAG-GEFI (9) and the binding of PIP_3_ with RASA3 using Mass Action kinetics. We then modeled the activation and inactivation of RAP1 using Michalis-Menten reaction terms, such that the differential equation for the surface density of RAP1-GTP is given by:

(2) $$S_{\text{PM}}\frac{d}{dt}{\left[\text{RAP}1-\text{GTP}\right]}_{S}=V_{\text{cyt}}\frac{k_{\text{GEF}}[\text{CaIDAG}-\text{GEFI}-2\text{Ca}]{\left[\text{RAP}1-\text{GDP}\right]}_{S}}{\left(\frac{V_{\text{Cyt}}}{S_{\text{PM}}}\right)K_{\text{GEF},\text{M}}+{\left[\text{RAP}1-\text{GDP}\right]}_{S}}-S_{\text{PM}}\frac{k_{\text{GAP}}{\left[\text{RASA}3\right]}_{S}{\left[\text{RAP}1-\text{GTP}\right]}_{S}}{\left(\frac{V_{\text{cyt}}}{S_{\text{PM}}}\right)K_{\text{GAP},\text{M}}+{\left[\text{RAP}1-\text{GTP}\right]}_{S}},$$

where $S_{\text{PM}}$ is the surface area of the cell membrane, $V_{\text{cyt}}$ is the volume of the cytosol, and the catalytic rates and Michalis-Menten constants are estimated in this work.

#### Simulation Protocol:

The copy number of each protein within mouse platelets has been documented using proteomics (11). From the data, we computed the concentration or surface density of every species in the model. The model is run in two stages: an equilibrium stage and an experimental stage. In the equilibrium stage, we started by assuming that every molecule is in a form that is not bound to any other molecule, and we assumed there is no external ADP. We then ran the system to steady-state, which we determined by computing the relative change in protein states at each iteration and stopping when changes decreased below a threshold. The concentration and surface density values at steady state were then used as the initial conditions in the experimental stage, where at t=30 s, the external ADP concentration was instantaneously increased to a set value, and the simulation was run for 600 s.

#### Sensitivity Analysis:

We briefly describe the Method of Morris, a one-at-a-time global sensitivity analysis algorithm. The algorithm gives a way to estimate the derivative of a model output with respect to model parameters when no closed form of the output exists and when dealing with many model parameters. In this study, we are interested in describing the derivative of the maximum RAP1-GTP value with respect to protein copy numbers.

The method begins by defining a hypercube in parameter space; for each dimension, the center is given by the average value of a specified protein’s copy number, and the half-width is given by the standard deviation of that protein’s copy number. Beginning at the center, the algorithm defines several paths to the boundary of the hypercube such that only one parameter changes with each step along a path. The paths were computed using Python’s SALib package (25, 26). Each vertex on a path specifies a parameter set used for a single simulation. In each simulation, we recorded the peak number of RAP1-GTP and then used finite differences to approximate the derivative of the maximum RAP1-GTP concentration with respect to the single parameter changed in that step. This process generated a sampling of the derivative of peak RAP1-GTP as a function of each parameter within the hypercube; for each parameter, we reported the mean μ and standard deviation σ of the approximation to the derivative. A large value of μ suggests a high sensitivity with respect to the given parameter, and a large value of σ suggests a high correlation between the given parameter and other parameters in the model. We again relied on SALib to compute μ and σ for each parameter.

#### Total integrin response metric:

JON/A is a fluorescent probe that binds tightly to the integrin $\alpha_{\text{IIV}}\beta_{3}$ in its high-affinity state. JON/A is assumed to bind irreversibly to the integrin, and enough JON/A is added to ensure rapid binding between the two species. To compare model outputs to previously published JON/A binding assays, we reported the integral with respect to time of RAP1-GTP over the 600 s after agonist application. See the supplemental for further details.

### Experimental procedures and data extraction

#### Flow cytometry experiments:

Washed platelets in Tyrode’s buffer were diluted to 10^7^ platelets/mL and loaded with 5 *μ*M Fluo-4 (Thermo Fisher Scientific) for 30 minutes at 37°C in the dark. Afterward, the samples were diluted to 10^6^ platelets/mL in Tyrode’s buffer and activated with 10 *μ*M ADP in the presence of 1 mM Ca^2+^ and Alexa647-labeled fibrinogen (100 *μ*g/ml, Sigma) while being continuously sampled on a BD C6 Plus flow cytometer. Kinetic calcium mobilization and fibrinogen binding were analyzed in Flow Jo (Version 10) as mean fluorescence intensities over time.

#### Parameter estimation:

**Figure 2:**
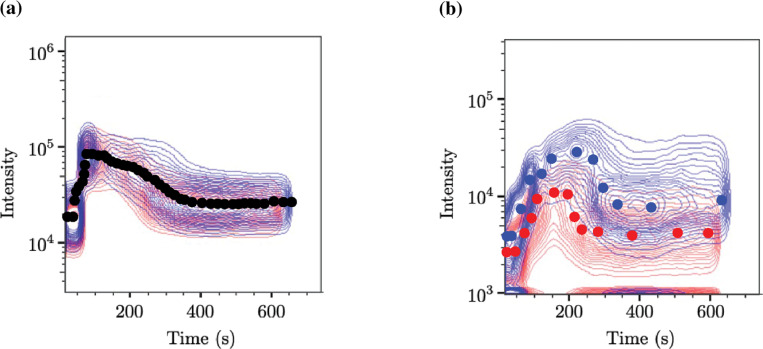
Flow cytometry experiments for WT (red) and RASA3^+/−^ (blue) platelets when 10 *μ*M of ADP was applied at 30 s. Scatter points indicate data that were collected for parameter estimation. Points were selected at approximately the peak fluorescence intensity for each specified time. (a) Fluorescent intensity of calcium-bound probe over time. (b) Fluorescent intensity of fibrinogen over time.

Previous models (13) of calcium signaling in cells and experimental work (11) with mice platelets have provided many of the rate constants and concentrations needed for this study. However, the rates governing the GEF and GAP action on RAP1 have yet to be entirely determined, and parameters estimated to fit prior calcium data were inconsistent with ours. Therefore, we performed a parameter estimation study to compare model outputs to our experimental data given in Figure 2. We first describe the method of converting fluorescence intensity to a measure of concentration and then the parameter estimation method.

For each dataset provided, we recorded the maximum intensity at select times to generate a single representative curve for each experiment. To convert calcium probe fluorescence intensity to concentration, we assumed that a relative increase in fluorescent intensity corresponds to an equivalent relative increase in the protein of interest. For calcium, we assumed a resting concentration of 40 nM and a peak concentration of 200 nM, in line with the literature (1). This data was used to estimate kinetic parameters for P2Y_1_, *G*_q_, SERCA, and PMCA. To reduce the number of parameters to estimate, we make the simplifying assumption that the kinetic parameters for P2Y_12_, G_i_, and PI3K are the same as those for P2Y_1_, G_q_, and PLC.

For fibrinogen data, we performed a similar rescaling, then assumed 1) that there are no fibrinogen bound to resting platelets and 2) a WT platelet activated by a saturating level of ADP allows an *a priori* known quantity of integrins to activate and 3) fibrinogen binds instantly to such an integrin. Finally, we used a 1:1 stochiometric relationship between RAP1-GTP and integrin to arrive at the amount of RAP1-GTP that is active intracellularly. We assume that either a maximum of 1% or 10% of integrins become activated, which equated to a maximum of 500 or 5000 RAP1-GTP, respectively; for either assumption, we ran a separate parameter estimation for rates governing CalDAG-GEFI, RASA3, and RAP1.

For any choice of parameters, we ran the model according to the simulation protocol described above. From the list of model outputs, we take the concentration of free cytosolic calcium and the number of activated RAP1 proteins, then compute the least squared error between the model and the datasets at specified times. The goal of the parameter estimation algorithm is to minimize this error, and for this, we relied on MATLAB’s fmincon function.

## RESULTS AND DISCUSSION

### Model outputs estimated to match experimental data:

**Figure 3:**
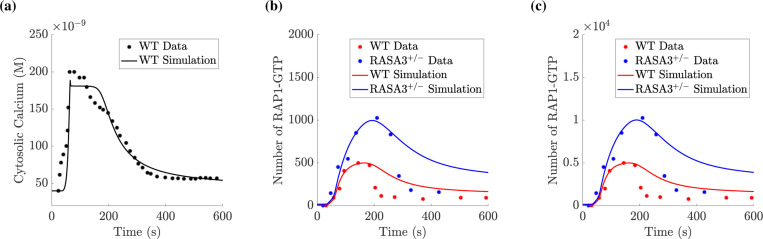
Parameter estimation allows for agreement between data and computational model. Solution to ODE system when using best-fit parameters. Using selected time points from the flow cytometry data, fluorescence intensity readings were rescaled and presented as scatter points. Solid curves depict results from the computational model. Black traces represent data/simulated calcium data, red traces represent data/simulated wildtype platelet RAP1-GTP, and blue traces represent data/simulated RASA3^+/−^ platelet RAP1-GTP. In simulations and experiments, 10 *μ*M of ADP was assumed to be added instantaneously at 30 s. (a) Calcium data was rescaled, assuming a resting concentration of 40 nM and peak concentration of 200 nM, and then it was overlayed with the computed free calcium concentration. (b) Fibrinogen binding data was assumed to correspond directly to RAP1-GTP levels at any given time and rescaled assuming no activated RAP1 at rest and a peak number of activated RAP1 of 500. (c) Using the same fibrinogen binding data, we assume a peak number of activated RAP1 of 5000.

Figure 3a compares a calcium spike dataset to the model’s calcium spike. Because the data and simulated calcium timecourse in the wildtype and RASA3 heterozygote are nearly identical, only the wildtype timecourse is given. The simulated calcium curves approximated the data well but smoothed over some of the dynamics near the peak.

Figures 3b-c show a comparison between the fibrinogen binding data and the simulated RAP1-GTP spike using the lower bound approximation (see Methods) of the data in Figure 3b and the upper bound approximation of the data in Figure 3c. For each of these assumptions about the number of integrins activated by ADP, our estimation procedure sought to minimize the sum of the mean squared differences between data and simulation for *both* the WT and RASA3+/− cases. Our model approximated the data well when ADP was applied to the peak RAP1-GTP response. However, simulations showed a slower return to baseline than the experimental data. For estimated parameter values, see Supplemental Tables **??** and **??**.

### Model sensitivity to protein copy number is unaffected by our data approximation:

Many of the simulations conducted in this work involve adjusting protein copy numbers. Therefore, understanding how our assumptions about the copy number data affected model outputs was useful. Thus, the global sensitivity analysis algorithm, the Method of Morris, was used to determine how changes in protein copy numbers change the maximum amount of RAP1-GTP seen in simulations. All protein copy numbers in the model were selected from the experimental literature (11), and each copy number was increased or decreased by one standard deviation from the mean.

**Figure 4:**
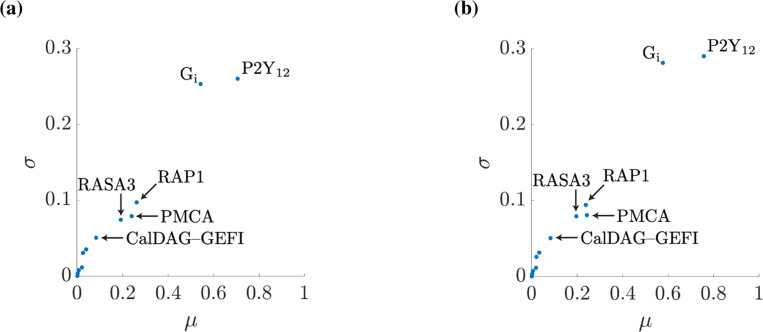
Sensitivity analysis reveals agreement between model outputs using either parameter set. Sensitivity of the maximum RAP1 response to protein copy numbers. Protein copy numbers are adjusted one at a time within a standard deviation of the literature value. For each adjustment made, the ratio of the relative change in maximum RAP1-GTP over the relative change in copy number is computed. The process is repeated for each protein at many points in parameter space. The ratios’sss average (μ) and standard deviation (σ) are presented. (a) Sensitivity of the model when using the lower bound estimated parameters as shown in Figure 3b. (b) Sensitivity of the model when using the upper bound estimated parameters as shown in Figure 3c. While thirteen proteins were examined for this study, only the proteins with a significant sensitivity were explicitly labeled.

Figure 4 shows the sensitivity of the maximum RAP1 response to the protein copy numbers. Comparing the two subfigures, we see little difference in the sensitivity of the model to copy numbers. We also note that in both figures, the same proteins have the most sensitivity, namely G_i_, P2Y_12_, PMCA, CalDAG-GEFI, and RASA3, meaning that a change in copy number of these proteins should yield similar relative responses. This served as evidence that under the appropriate normalization, the results generated by one estimated parameter set are similar to those generated by the other. We therefore present results using only our upper bound estimate parameter set for the remainder of this work.

Moreover, Figure 4 shows that the peak level of RAP1-GTP was most sensitive to changes in the P2Y_12_ receptor and G_i_ protein. This implies that the variability in expression of P2Y_12_ or G_i_ had the most significant effect on RAP1 activation. In particular, equivalent variability in the expression of RASA3, the immediate effector of RAP1, did not produce the same variation in the RAP1-GTP signal.

### model predicts saturating integrin response to ADP:

**Figure 5:**
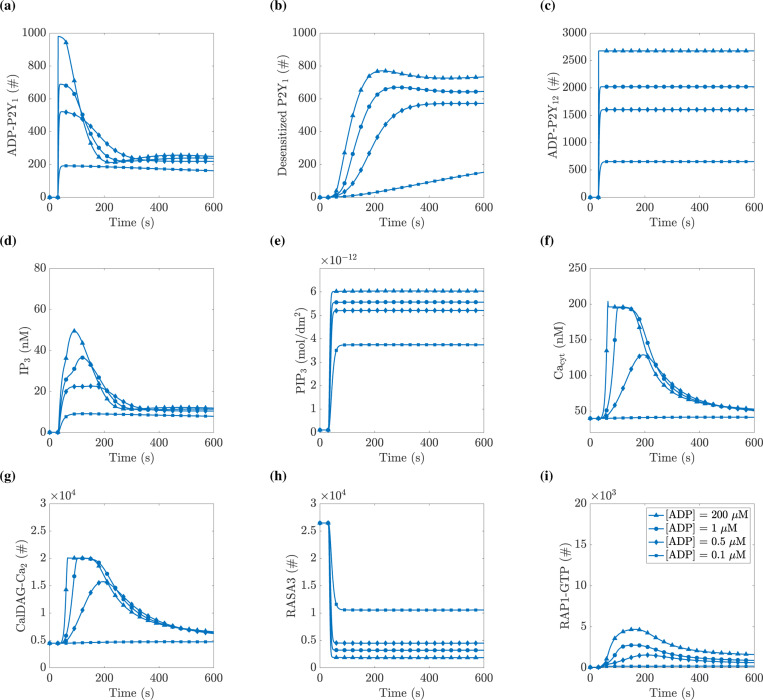
Model predicts transient activation of RAP1-GTP across ADP concentrations. At t=30 s, a specified concentration of ADP was instantaneously added to the extracellular space. The curves shown are for ADP at 0.1 *μ*M (squares), 0.5 *μ*M (diamonds), 1 *μ*M (circles), and 200 *μ*M (triangles). (a) the total number of P2Y_1_ receptors bound to ADP and not desensitized, (b) the total number of desensitized P2Y_1_ receptors, (c) the total number of P2Y_12_ receptors bound to ADP, (d) the total concentration of IP_3_, (e) the total surface density of PIP_3_, (f) the concentration of free cytosolic calcium, (g) the number of CalDAG-GEFI molecules bound with two calcium ions, (h) the number of RASA3 not bound to PIP_3_, and (i) the number of RAP1 bound to GTP. See Section **??** for details on which model equations were used in calculating total number or concentration.

We then examined how our simulated platelet responds to a range of applied agonist concentrations. Figure 5 shows how the numbers or concentrations of various chemicals changed over time for a given application of ADP at t=30 s. Figures 5a and 5c show that immediately upon application of ADP into the system, there was a rapid rise in the number of P2Y_1_ and P2Y_12_ receptors, respectively, bound with ADP. When 200 *μ*M of ADP was applied, essentially all P2Y_1_ and P2Y_12_ receptors were bound to ADP at the peak. Due to PKC’s desensitization of the P2Y_1_ receptor, the number of active P2Y_1_ receptors decreased from its peak value as the simulation progressed. Interestingly, the number of active P2Y_1_ receptors decreased most rapidly for the highest concentration of ADP. This decrease was matched by a fast rise in the number of desensitized P2Y_1_ receptors, as shown in Figure 5b. Therefore, as the concentration of ADP applied increased, the maximum response increased, but the time interval on which a near-peak level was maintained decreased.

Following receptor activation, second messengers PLC and PI3K became active by binding the appropriate G protein, leading to a rise in the production of the inositol species IP_3_ and PIP_3_, respectively. Figure 5d shows that the IP_3_ concentration rose with a slight delay compared to the receptor concentrations. Later in the simulations, as the P2Y_1_ receptors become desensitized, the rate of formation of IP_3_ decreased, leading to a decline in its concentration.

IP_3_ then activated IP_3_R, which led to an influx of calcium into the cytosol from the DTS. As seen in Figure 5f, the level of free cytosolic calcium increased, plateaued, and then decreased throughout the simulation. The rise in cytosolic calcium levels led to the activation of proteins PKC and CalDAG-GEFI, the latter of which is seen in Figure 5g following a similar pattern of rise, plateau, and decrease as the corresponding calcium trace.

Concurrently with IP_3_, calcium, and CalDAG-GEFI spiking, the P2Y_12_ pathway led to the formation of the PIP_3_ inositol species as seen in Figure 5e, followed by the inactivation of RASA3, as seen in Figure 5h. Unlike in the P2Y_1_ pathway, the lack of a desensitization mechanism for P2Y_12_ meant that PIP_3_ was constantly created by PI3K during the simulation, leading to a permanent decrease in the number of active RASA3 molecules.

The activation of CalDAG-GEFI and inactivation of RASA3 caused an increase in the amount of RAP1 bound to GTP, shown in Figure 5i. When the CalDAG-GEFI levels decreased, RAP1-GTP levels also decreased and approached their baseline levels. As shown more explicitly in Figure 8, the model’s response to ADP saturated around 10 *μ*M, so the 200 *μ*M ADP simulated experiment represents the maximum ADP response.

### RAP1 activation response increases with applied agonist:

We next attempted to validate our model’s output by comparison to data previously published (27). To do this, we simulated platelets’ exposure to ADP, which binds to both P2Y_1_ and P2Y_12_ receptors, and platelets’ exposure to the synthetic agonist MRS2365, which binds only to the P2Y_1_ receptor. To accommodate the synthetic agonist, the model was changed in two ways: 1) by setting the binding rate between agonist and P2Y_12_ to zero, i.e., $k_{\text{ADP}}^{\text{P}2{\text{Y}}_{12}}=0$, and 2) by lowering the dissociation constant for the agonist binding to P2Y_1_ from 0.6 to 0.01 *μ*M, i.e. $K_{\text{ADP}}^{\text{P}2{\text{Y}}_{1}}=0.01\;\mu \text{M}$.

**Figure 6:**
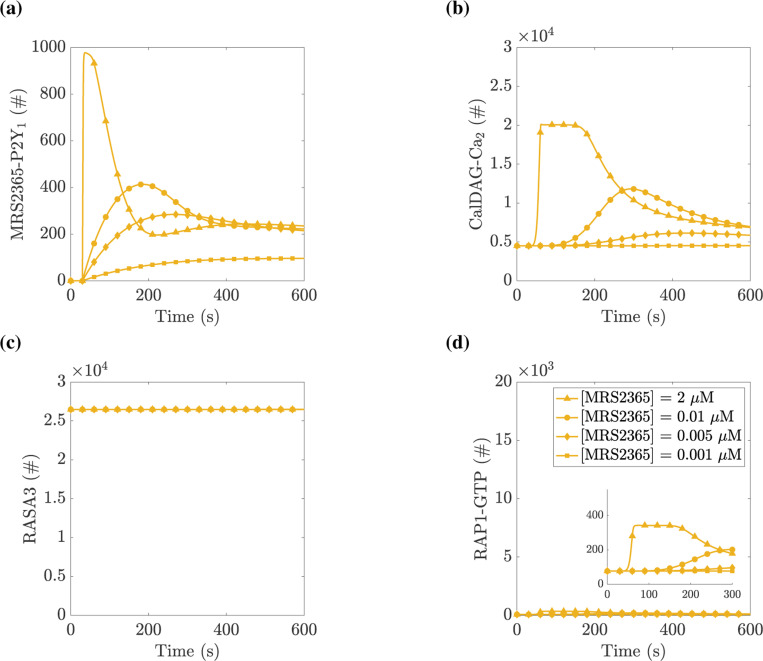
Simulated platelets exposed to MRS2365 show significantly reduced RAP1-GTP response. At t=30 s, a specified concentration of MRS2365 was instantaneously added to the extracellular space. The curves shown are for MRS2365 at 0.001 *μ*M (squares), 0.005 *μ*M (diamonds), 0.01 *μ*M (circles), and 2 *μ*M (triangles). (a) the total number of P2Y_1_ receptors bound to MRS2365 and not desensitized, (b) the number of CalDAG-GEFI molecules bound with two calcium ions, (c) the number of RASA3 not bound to PIP_3_, and (d) the number of RAP1 bound to GTP. See Section **??** for details on which model equations were used in calculating total number or concentration.

Figure 6 shows how WT platelets respond to the synthetic agonist. We found that the application of a saturating amount of MRS2365 yielded similar levels of P2Y_1_ and CalDAG-GEFI activation compared to those evoked by the application of a saturating amount of ADP (compare Figure 6a with Figure 5a and Figure 6b with Figure 5g). However, because the concentration of RASA3 remained high throughout the MRS2365 simulations, RAP1 activation was greatly limited.

**Figure 7:**
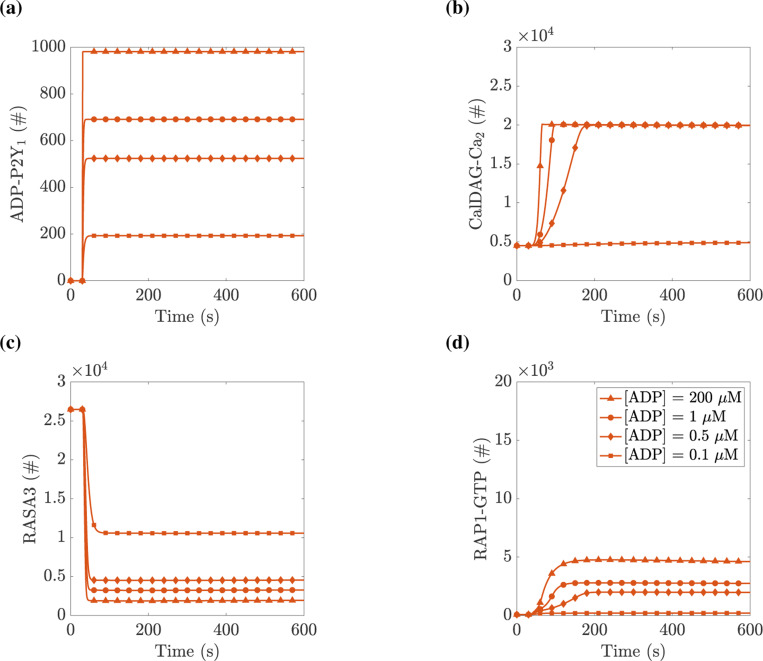
Simulated platelets lacking PKC-dependent P2Y_1_ desensitization show a significantly prolonged RAP1-GTP signal. At t=30 s, a specified concentration of ADP was instantaneously added to the extracellular space of $\text{P}2{\text{Y}}_{1}^{340-0\text{P}/340-0\text{P}}$ platelets. The curves shown are for ADP at 0.1 *μ*M (squares), 0.5 *μ*M (diamonds), 1 *μ*M (circles), and 200 *μ*M (triangles). (a) the total number of P2Y_1_ receptors bound to ADP, (b) the number of CalDAG-GEFI molecules bound with two calcium ions, (c) the number of RASA3 not bound to PIP_3_, and (d) the number of RAP1 bound to GTP. See Section **??** for details on which model equations were used in calculating total number or concentration.

We next considered platelets with a mutation to the intracellular tail of their P2Y_1_ receptor, which makes it unable to be phosphorylated and therefore the receptor is prevented from being desensitized to agonists and being taken up into the cell (27). These platelets are known as $\text{P}2{\text{Y}}_{1}^{340-0\text{P}/340-0\text{P}}$ platelets or simply 340–0P. Figure 7 shows how a 340–0P platelet responds to ADP. While the peak value of various proteins was similar to the wildtype case seen in Figure 5, the simulated mutant platelet’s P2Y_1_ signal never declined, and consequently the downstream calcium, CDGI, and RAP1 signals also did not decline.

**Figure 8:**
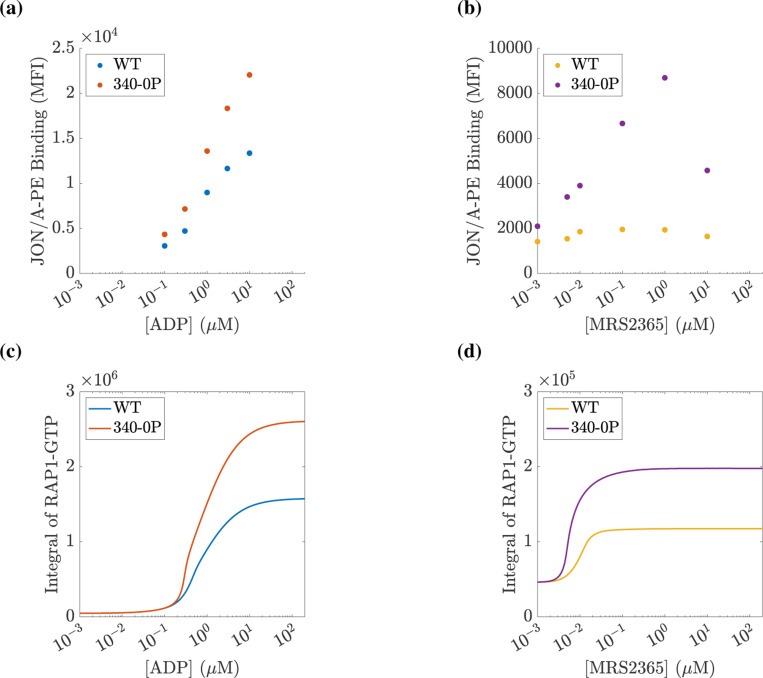
Total integrin response displays a saturating response to two types of P2Y agonists. Wildtype and $\text{P}2{\text{Y}}_{1}^{340-0\text{P}/340-0\text{P}}$ platelets are stimulated with a specified concentration of agonists for 10 minutes, and the time-integral of the RAP1-GTP curve is recorded. Subfigures (a) and (b) are recreated from (27) Figures 2c and 4c. (a) Mean Fluorescence Intensity of JON/A-PE binding to active $\alpha_{\text{IIb}}\beta_{3}$ integrins after a specified concentration of ADP is applied. (b) Mean Fluorescence Intensity of JON/A-PE binding to active $\alpha_{\text{IIb}}\beta_{3}$ integrins after a specified concentration of MRS2365 is applied. (c) Platelets are exposed to ADP, and (d) platelets are exposed to P2Y_1_ agonist MRS2365. Note the differences in vertical scales.

To gauge variations in platelet responses due to variations in agonist concentration, we performed computational experiments for a wide range of applied ADP and MRS2365 concentrations. In the corresponding physical experiments, the readout was the amount of the integrin probe JON/A bound irreversibly to activated integrin. As a proxy for the amount of JON/A bound at time t we used the time-integral of the RAP1-GTP curve up to that time. Figure 8 shows the results of these experiments. As expected, for both agonists, the RAP1-GTP response increased as the concentration of the agonist was increased. This response was consistently greater for 340–0P platelets than for WT ones. This is attributed to the fact that for the $\text{P}2{\text{Y}}_{1}^{340-0\text{P}/340-0\text{P}}$ platelets, the P2Y_1_ receptors remained active and continued to perform their GEF action on the G_q_ protein for the duration of the simulation. Consequently, the IP_3_, calcium, and RAP1-GTP responses are prolonged, and therefore, the area under the RAP1-GTP curve increased.

Two differences were evident in how both wildtype and 340–0P platelets responded to the two stimuli. First, the response to MRS2365 began at lower concentrations than that to ADP, consistent with the higher affinity of MRS2365 for the P2Y_1_ receptor. Second, the response to a saturating concentration of MRS2365 was lower than for a saturating concentration of ADP, consistent with unabated RASA3 GAP activity in the MRS2365-stimulated platelets.

We compared the results of Figures 2c and 4c in (27), recreated in Figure 8a and 8b, to the results of Figures 8c and 8d. First, comparing the experiments in which platelets are stimulated with ADP in Figure 8a and 8c, we saw an increase in the integrin response over the same range of applied ADP concentration for both wildtype and $\text{P}2{\text{Y}}_{1}^{340-0\text{P}/340-0\text{P}}$ platelets. Additionally, we saw that for a given applied ADP concentration, the 340–0P platelet signal was consistently larger than the wildtype platelet. We note that in Figure 8b and 8d, we saw a similar relationship between mutant and wildtype platelets. Deviations between the model and experiment are evident when comparing simulated versus experimental mutant platelets stimulated with MRS2365, where the experiments show a more gradual increase in integrin activation as a function of applied agonist concentration compared to simulations. In general, comparing our model to results from (27) showed good agreement, even when a single experimental modification is considered. As more modifications are combined into simulated platelets, the results may deviate from experiments.

### Changes to protein copy number in the P2Y_12_ pathway affect RAP1-GTP response more than in the P2Y_1_ pathway:

The model allowed for the continuous variation of parameters, specifically protein copy numbers. We leveraged this to assess the effects of protein expression levels on the integrin activation response to a specified stimulus. In each set of simulations, we set the copy number of one of the proteins RASA3, CDGI, P2Y_1_, or P2Y_12_ to a value between 5% and 150% of its literature value (while holding other copy numbers at their literature values). We stimulated the platelet with 10 *μ*M ADP.

**Figure 9:**
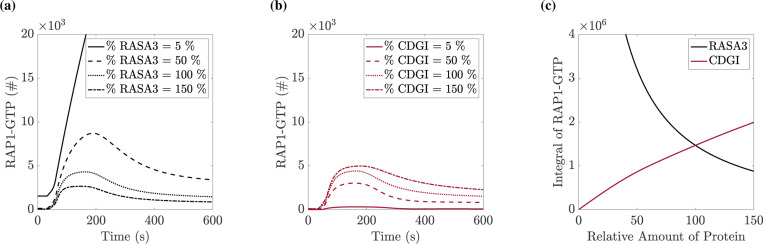
Total integrin response is more sensitive to RASA3 levels than CalDAG-GEFI levels. In each simulation, the starting copy number of one protein is varied between 5% and 150% of the literature value before applying 10 *μ*M ADP. (a) Timecourse of RAP1-GTP levels in platelets expressing a specified percentage of the base RASA3 copy number. (b) Timecourse of RAP1-GTP levels in platelets expressing a certain percentage of the base CalDAG-GEFI copy number. (c) Integral of the RAP1-GTP curve as one of the two protein copy numbers is varied from 5% to 150% of literature value.

Figure 9 shows the results of varying the RASA3 or CDGI copy numbers. We saw that a decrease in the number of RASA3 molecules or an increase in the number of CDGI molecules elicits an increase in the RAP1-GTP response. Figure 9a shows that when the copy number of RASA3 is decreased by 50%, integrin activation was stronger and more prolonged than the same simulations with 100% RASA3 expression. In contrast, Figure 9b shows that increasing the amount of CDGI by 50% did not significantly change the peak level but did increase the length of the RAP1-GTP response.

Figure 9c plots the RAP1-GTP response as a function of a specified protein’s copy number, where we saw that the sensitivity of the response to RASA3 copy number variations was much greater than that for CDGI variations. Changes in the CDGI copy number led to a small and approximately linear change in RAP1 activation. In contrast, responses to decreases in the RASA3 copy number are nonlinear, with a sharp increase in sensitivity as the RASA3 copy number decreases to and beyond 50% of its baseline value.

**Figure 10:**
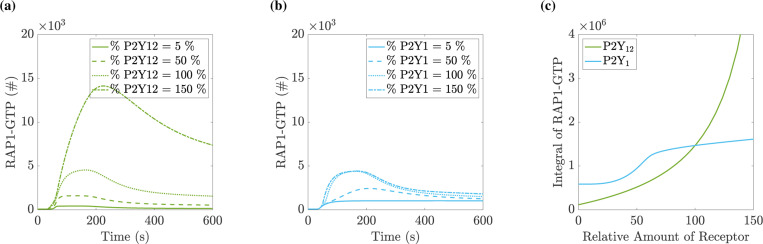
Total integrin response is more sensitive to P2Y_12_ levels than to P2Y_1_ levels. In each simulation, the starting copy number of one protein is varied between 5% and 150% of the literature-derived copy number before the application of 10 *μ*M ADP. (a) Timecourse of RAP1-GTP levels in platelets expressing a specified percentage of the base P2Y_12_ copy number. (b) Timecourse of RAP1-GTP levels in platelets expressing a certain percentage of the base P2Y_1_ copy number. (c) Integral of the RAP1-GTP curve as one of the two protein copy numbers is varied.

Figure 10 shows a similar dichotomy between responses to variations in copy numbers of the ADP receptors P2Y_1_ and P2Y_12_. With P2Y_12_ at 150%, integrin activation was much greater at all times, and high levels were maintained for an extended period compared to 100%. With P2Y_12_ at 50%, integrin activation is weak. With P2Y_1_ at 50%, integrin activation was approximately half that of activation at 100%. There was only a small difference in integrin activation for P2Y_1_ 100% and 150%. Examining model outputs, in this case, showed that although the amount of activated IP_3_ increased, the peak concentration of calcium did not increase significantly beyond 200 nM (data not shown).

## CONCLUSION

In this report, we developed a mathematical model of integrin activation on a platelet surface and fitted it to experimental data. Thanks to years of experimental studies, a chemical pathway has been defined sufficiently to allow such a model to exist. Previous platelet activation models rely on other major indicators, such as calcium signals or aggregometry readings (28, 29). While effective, inferring integrin activation information from only one of the two ADP-dependent pathways does not fully encapsulate the regulatory pathway’s complexity, making it difficult to extend to particular mutant platelets. Our model can be used in testing and making predictions without the need for a large number of experimental trials. It is quite economical to run; for reference, a single simulation takes approximately one minute to run ten simulated minutes on a standard personal computer. Thus, data generation is significantly faster than generation of an equivalent amount of data in the lab.

While our estimated parameters yielded a model that matched our experimental dataset from the point of ADP addition up to the peak, our model tended to lengthen the duration of the RAP1-GTP signal. Under the assumption that our estimated parameters are the ideal parameters for the model, a mechanism for desensitization of some signaling protein in the P2Y_12_ pathway, leading to a recovery of RASA3 levels, could shorten the RAP1-GTP signal duration. Without sufficient evidence for a biological mechanism, we save modeling this portion of the pathway for future work.

In experiments where we varied the applied ADP concentration, we found that a platelet’s response saturates beyond 10 *μ*M, consistent with experiments. Our highly detailed model allowed us to examine the individual responses of each protein in time. We showed the usefulness of our model beyond directly comparing to experimental data by varying parameters that would be difficult to vary continuously in the lab, like protein expression levels, and making predictions on integrin activation. In particular, we showed that the total integrin response varies more significantly with changes to proteins in the P2Y_12_ pathway compared to the P2Y_1_ pathway.

This model can be used to conduct initial tests on the behavior of platelets under any form of experimental condition before running experiments. In addition, the outputs of this model describe integrin activation over time in far more detail than is typical in mathematical models for hemostasis and thrombosis (30, 31), where the tracking of platelet aggregation and fibrin formation is of interest. This may lead to improvements in these larger-scale models.

## Supplementary Material

Supplement 1

## SIGNIFICANCE

A detailed dynamical systems model of integrin αIIbβ3 activation mediated by ADP is presented in this report. This model takes in years of experimental literature on the relevant pathways into simulations that are efficient to run and simple to manipulate parameters to match a platelet mutation. Unlike in experiments, our model can finely adjust any parameter and analyze how a platelet responds to any number of experimental settings. We present the utility of such a model through parameter study on the copy number of proteins key to integrin activation and show that integrin response changes more significantly in response to changes in the P2Y_12_ pathway over the P2Y_1_ pathway.
