# Supplementary material for: Modeling Platelet P2Y1/12 Pathway to Integrin Activation
Source: ArXiv. 2024 Oct 16:arXiv:2410.13015v1. Preprint. [Version 1] (PMC11527272)
Supplement: Supplementary file 1 [file NIHPP2410.13015v1-supplement-1.pdf]

## Contents


S1 Comparing integral of RAP1-GTP to JON/A binding assay . . . 2
S2 Grouped quantities reported in main text . . . 2
S3 Estimated Parameters . . . 2
S4 Full List of Model Equations . . . 4
S4.1 CalDAG-GEFI, RASA3, and RAP1 . . . 5
S4.1.1 CalDAG-GEFI . . . 5
S4.1.2 RASA3 . . . 6
S4.1.3 RAP1 . . . 7
S4.2 ADP . . . 8
S4.3 Proteins in $P2Y_{12}$ Pathway . . . 9
S4.3.1 $P2Y_{12}$ . . . 9
S4.3.2 $G_i$ . . . 13
S4.3.3 PI3K . . . 15
S4.4 Proteins in $P2Y_1$ pathway . . . 17
S4.5 $P2Y_1$ . . . 17
S4.5.1 $G_q$ . . . 21
S4.5.2 PLC . . . 23
S4.5.3 PKC . . . 26
S4.6 Calcium and Inositol Species . . . 28
S4.6.1 Inositol . . . 28
S4.6.2 Calcium . . . 33
S4.7 Calcium channels/pumps . . . 35
S4.7.1 $IP_3R$ . . . 35
S4.7.2 SERCA . . . 38
S4.7.3 PMCA . . . 40
S5 Quasi Steady State Approximation . . . 42


arXiv:2410.13015v1 [q-bio.MN] 16 Oct 2024

# S1 Comparing integral of RAP1-GTP to JON/A binding assay

Given that we already assume a one-to-one stoichiometric relationship between RAP1-GTP and integrin, we can write down the following ODE for the rate at which integrin binds to JON/A:

$$\frac{d}{dt}[\mathrm{integrin}-\mathrm{probe}]_S = k[\mathrm{probe}][\mathrm{RAP1}-\mathrm{GTP}]_S. \quad (1)$$

Assuming that 1) ADP is instantaneously added to the extracellular space at $t = 30$ s and 2) the total amount of probe is large compared to the number of integrins, we can explicitly integrate the equation over 600 s to find that:

$$[\mathrm{integrin}-\mathrm{probe}]_S(630) = k[\mathrm{probe}]\int_{30}^{630}[\mathrm{RAP1}-\mathrm{GTP}]_S(t)dt. \quad (2)$$

Thus, to find the amount of integrin bound to probe, we simply integrate the RAP1-GTP curve.

# S2 Grouped quantities reported in main text

In some cases in our model, a chemical species of interest (say, the $\mathrm{P2Y_1}$ receptor), is further split into multiple states. If we would like to know the total number of receptors with an ADP bound to it at time $t$, we sum up the amounts from each state and report the summed quantity. In particular, we make use of the following sums:

- $\mathrm{ADP-P2Y_1} \;=\; [\mathrm{ADP-P2Y_1}]_\mathrm{S} + [\mathrm{ADP-P2Y_1-G_qGDP}]_\mathrm{S} + [\mathrm{ADP-P2Y_1-G_qGTP}]_\mathrm{S} + [\mathrm{ADP-P2Y_1-G_q}]_\mathrm{S}$
- $\mathrm{ADP-P2Y_{12}} \;=\; [\mathrm{ADP-P2Y_{12}}]_\mathrm{S} + [\mathrm{ADP-P2Y_{12}-G_iGDP}]_\mathrm{S} + [\mathrm{ADP-P2Y_{12}-G_iGTP}]_\mathrm{S} + [\mathrm{ADP-P2Y_{12}-G_i}]_\mathrm{S}$
- $\mathrm{Desensitized\ P2Y_1} \;= [\mathrm{ADP-pP2Y_1}]_\mathrm{S} + [\mathrm{ADP-pP2Y_1-G_qGDP}]_\mathrm{S}$
- $\mathrm{IP_3} \;=\; [\mathrm{IP_3}] + \frac{\mathrm{S_{IM}}}{\mathrm{V_{cyt}}}\Big([\mathrm{IP_3R_o}]_\mathrm{S} + [\mathrm{IP_3R_a}]_\mathrm{S} + [\mathrm{IP_3R_{i2}}]_\mathrm{S} + [\mathrm{IP_3R_s}]_\mathrm{S}\Big)$

# S3 Estimated Parameters

| Parameter | Value (Max Calcium 100 nM) | Value (Max Calcium 200 nM) | Units |
|---|---|---|---|
| $K_{\mathrm{act}}^{\mathrm{P2Y_1}}$ & $K_{\mathrm{act}}^{\mathrm{P2Y_{12}}}$ | $4.906 \cdot 10^{-3}$ | $4.806 \cdot 10^{-3}$ | n.d. |
| $\alpha^{\mathrm{P2Y_1}}$ & $\alpha^{\mathrm{P2Y_{12}}}$ | 1.782 | 1.782 | n.d. |
| $\beta^{\mathrm{P2Y_1}}$ & $\beta^{\mathrm{P2Y_{12}}}$ | 8.573 | 8.508 | n.d. |
| $\delta^{\mathrm{P2Y_1}}$ & $\delta^{\mathrm{P2Y_{12}}}$ | $1.46 \cdot 10^{1}$ | $1.466 \cdot 10^{1}$ | n.d. |
| $\gamma^{\mathrm{P2Y_1}}$ & $\gamma^{\mathrm{P2Y_{12}}}$ | $2.324 \cdot 10^{1}$ | $2.361 \cdot 10^{1}$ | n.d. |
| $k_{\mathrm{G_qGDP}}^{\mathrm{P2Y_1}}$ & $k_{\mathrm{G_iGDP}}^{\mathrm{P2Y_{12}}}$ | $6.947 \cdot 10^{13}$ | $6.947 \cdot 10^{13}$ | $\mathrm{dm^2/mol\text{-}s}$ |
| $k_{-\mathrm{G_qGDP}}^{\mathrm{P2Y_1}}$ & $k_{-\mathrm{G_iGDP}}^{\mathrm{P2Y_{12}}}$ | $5.858 \cdot 10^{3}$ | $5.858 \cdot 10^{3}$ | 1/s |
| $k_{\mathrm{ADP}}^{\mathrm{P2Y_1}}$ & $k_{\mathrm{ADP}}^{\mathrm{P2Y_{12}}}$ | $5.716 \cdot 10^{5}$ | $5.983 \cdot 10^{5}$ | 1/M-s |
| $k_{-\mathrm{ADP}}^{\mathrm{P2Y_1}}$ & $k_{-\mathrm{ADP}}^{\mathrm{P2Y_{12}}}$ | 0.362 | 0.362 | 1/s |
| $k_{-\mathrm{GDP}}^{\mathrm{P2Y_1}}$ & $k_{-\mathrm{GDP}}^{\mathrm{P2Y_{12}}}$ | $2.625 \cdot 10^{1}$ | $2.601 \cdot 10^{1}$ | 1/s |
| $k_{\mathrm{GDP}}^{\mathrm{P2Y_1}}$ & $k_{\mathrm{GDP}}^{\mathrm{P2Y_{12}}}$ | $1.248 \cdot 10^{6}$ | $1.30 \cdot 10^{6}$ | 1/M-s |
| $k_{-\mathrm{GTP}}^{\mathrm{P2Y_1}}$ & $k_{-\mathrm{GTP}}^{\mathrm{P2Y_{12}}}$ | 8.057 | 8.077 | 1/s |
| $k_{\mathrm{GTP}}^{\mathrm{P2Y_1}}$ & $k_{\mathrm{GTP}}^{\mathrm{P2Y_{12}}}$ | $2.185 \cdot 10^{4}$ | $2.189 \cdot 10^{4}$ | 1/M-s |
| $k_{-\mathrm{G_qGTP}}^{\mathrm{P2Y_1}}$ & $k_{-\mathrm{G_iGTP}}^{\mathrm{P2Y_{12}}}$ | $3.358 \cdot 10^{-1}$ | $3.315 \cdot 10^{-1}$ | 1/s |
| $k_{\mathrm{G_qGTP}}^{\mathrm{P2Y_1}}$ & $k_{\mathrm{G_iGTP}}^{\mathrm{P2Y_{12}}}$ | $2.65 \cdot 10^{15}$ | $2.65 \cdot 10^{15}$ | $\mathrm{dm^2/mol\text{-}s}$ |
| $k_{-\mathrm{G_{q\alpha}GDP}}^{\mathrm{PLC}}$ & $k_{-\mathrm{G_{i\alpha}GDP}}^{\mathrm{PI3K}}$ | $1.247 \cdot 10^{1}$ | $1.247 \cdot 10^{1}$ | 1/s |

| | | | |
|---|---|---|---|
| $k^{\mathrm{PLC}}_{-\mathrm{G_{q\alpha}GTP}}$ & $k^{\mathrm{PI3K}}_{-\mathrm{G_{i\alpha}GTP}}$ | $1.207 \cdot 10^{-3}$ | $1.207 \cdot 10^{-3}$ | 1/s |
| $k^{\mathrm{PLC}}_{\mathrm{G_{q\alpha}GDP}}$ & $k^{\mathrm{PI3K}}_{\mathrm{G_{i\alpha}GDP}}$ | $6.098 \cdot 10^{11}$ | $6.098 \cdot 10^{11}$ | dm$^2$/mol-s |
| $k^{\mathrm{PLC}}_{\mathrm{G_{q\alpha}GTP}}$ & $k^{\mathrm{PI3K}}_{\mathrm{G_{i\alpha}GTP}}$ | $6.044 \cdot 10^{16}$ | $6.044 \cdot 10^{16}$ | dm$^2$/mol-s |
| $k^{\mathrm{PLC}}_{\mathrm{hydrolyze}}$ & $k^{\mathrm{PI3K}}_{\mathrm{hydrolyze}}$ | 1.293 | 1.189 | 1/s |
| $k_{\mathrm{GTP}}$ | $1.776 \cdot 10^{1}$ | $1.851 \cdot 10^{1}$ | 1/s |
| $k^{\mathrm{P2Y_1}}_{\mathrm{phos}}$ | $2.78 \cdot 10^{8}$ | $1.485 \cdot 10^{8}$ | 1/M-s |
| $k^{\mathrm{P2Y_1}}_{\mathrm{dephos}}$ | $1.313 \cdot 10^{-3}$ | $4.152 \cdot 10^{-3}$ | 1/s |
| $\gamma^{\mathrm{IP_3R}}$ | $4.286 \cdot 10^{-10}$ | $4.171 \cdot 10^{-10}$ | S |
| $\gamma^{\mathrm{leak}}$ | $7 \cdot 10^{-12}$ | $7.026 \cdot 10^{-12}$ | S/dm$^2$ |
| $k^{\mathrm{SERCA}}_{\mathrm{2f}}$ | $1.033 \cdot 10^{12}$ | $8.877 \cdot 10^{11}$ | 1/M$^2$-s |
| $k^{\mathrm{SERCA}}_{\mathrm{2r}}$ | $1.375 \cdot 10^{2}$ | $1.368 \cdot 10^{2}$ | 1/s |
| $k^{\mathrm{SERCA}}_{\mathrm{3f}}$ | $5.532 \cdot 10^{2}$ | $5.59 \cdot 10^{2}$ | 1/s |
| $k^{\mathrm{SERCA}}_{\mathrm{3r}}$ | $1.003 \cdot 10^{1}$ | 9.887 | 1/s |
| $k^{\mathrm{SERCA}}_{\mathrm{4f}}$ | $3.563 \cdot 10^{2}$ | $3.829 \cdot 10^{2}$ | 1/s |
| $k^{\mathrm{SERCA}}_{\mathrm{4r}}$ | $8.559 \cdot 10^{1}$ | $8.419 \cdot 10^{1}$ | 1/s |
| $k^{\mathrm{SERCA}}_{\mathrm{5f}}$ | $5.724 \cdot 10^{2}$ | $2.193 \cdot 10^{1}$ | 1/s |
| $k^{\mathrm{SERCA}}_{\mathrm{5r}}$ | $3.662 \cdot 10^{9}$ | $3.619 \cdot 10^{9}$ | 1/M$^2$-s |
| $k^{\mathrm{PMCA}}_{\mathrm{2f}}$ | $4.484 \cdot 10^{10}$ | $4.704 \cdot 10^{10}$ | 1/M$^2$-s |
| $k^{\mathrm{PMCA}}_{\mathrm{2r}}$ | $4.742 \cdot 10^{2}$ | $4.423 \cdot 10^{2}$ | 1/s |
| $k^{\mathrm{PMCA}}_{\mathrm{3f}}$ | $2.071 \cdot 10^{9}$ | $2.23 \cdot 10^{9}$ | 1/s |
| $k^{\mathrm{PMCA}}_{\mathrm{3r}}$ | $2.773 \cdot 10^{2}$ | $2.641 \cdot 10^{2}$ | 1/s |
| $k^{\mathrm{PMCA}}_{\mathrm{4f}}$ | 1.456 | 1.337 | 1/s |
| $k^{\mathrm{PMCA}}_{\mathrm{4r}}$ | $1.355 \cdot 10^{3}$ | $1.338 \cdot 10^{3}$ | 1/s |
| $k^{\mathrm{PMCA}}_{\mathrm{5f}}$ | $7.614 \cdot 10^{-1}$ | 1.925 | 1/s |
| $k^{\mathrm{PMCA}}_{\mathrm{5r}}$ | $1.843 \cdot 10^{5}$ | $1.105 \cdot 10^{4}$ | 1/M$^2$-s |

Tab. S1: Parameter values estimated against calcium data. The parameter estimation was run twice; once where it was assumed the peak calcium concentration reached 100 nM, and the second where it was assumed the peak calcium concentration reached 200 nM. The estimated values from the 200 nM assumption were used in the results of the main paper.

| Parameter | max RAP1-GTP = 500 (Max Ca 100 nM) | max RAP1-GTP = 5000 (Max Ca 100 nM) | max RAP1-GTP = 500 (Max Ca 200 nM) | max RAP1-GTP = 5000 (Max Ca 200 nM) | Units |
|---|---|---|---|---|---|
| $k^{\mathrm{PI3K}}_{\mathrm{PIP_2}}$ | $1 \cdot 10^{8}$ | $1 \cdot 10^{8}$ | $1 \cdot 10^{8}$ | $1 \cdot 10^{8}$ | 1/M-s |
| $k^{\mathrm{PI3K}}_{-\mathrm{PIP_2}}$ | 6870 | 6870 | 6870 | 6870 | 1/s |
| $k^{\mathrm{PI3K}}_{\mathrm{PIP_2,cat}}$ | 33.15 | 33.15 | 33.15 | 33.15 | 1/s |
| $k^{\mathrm{PIP_3Phosphatase}}_{-\mathrm{PIP_3,cat}}$ | 20 | 20 | 20 | 20 | 1/s |
| $K^{\mathrm{PIP_3Phosphatase}}_{-\mathrm{PIP_3,M}}$ | $2.5 \cdot 10^{-7}$ | $2.5 \cdot 10^{-7}$ | $2.5 \cdot 10^{-7}$ | $2.5 \cdot 10^{-7}$ | M |
| $k^{\mathrm{RASA3}}_{\mathrm{PIP_3}}$ | $4.8 \cdot 10^{10}$ | $4.8 \cdot 10^{10}$ | $4.8 \cdot 10^{10}$ | $4.8 \cdot 10^{10}$ | 1/M-s |
| $k^{\mathrm{RASA3}}_{-\mathrm{PIP_3}}$ | $1.6 \cdot 10^{3}$ | $1.6 \cdot 10^{3}$ | $1.6 \cdot 10^{3}$ | $1.6 \cdot 10^{3}$ | 1/s |
| $k^{\mathrm{GAP}}_{\mathrm{cat}}$ | $1.6 \cdot 10^{-2}$ | $1.011 \cdot 10^{-1}$ | $1.585 \cdot 10^{1}$ | $1.239 \cdot 10^{1}$ | 1/s |
| $K^{\mathrm{GAP}}_{\mathrm{M}}$ | $4.922 \cdot 10^{-8}$ | $8.078 \cdot 10^{-7}$ | $2.441 \cdot 10^{-4}$ | $1.666 \cdot 10^{-4}$ | M |
| $k^{\mathrm{GEF}}_{\mathrm{cat}}$ | $2.305 \cdot 10^{-3}$ | $1.5 \cdot 10^{-2}$ | $6.652 \cdot 10^{-3}$ | $4.0 \cdot 10^{-2}$ | 1/s |
| $K^{\mathrm{GEF}}_{\mathrm{M}}$ | $4.896 \cdot 10^{-6}$ | $2.412 \cdot 10^{-5}$ | $3.848 \cdot 10^{-4}$ | $1.915 \cdot 10^{-4}$ | M |

Tab. S2: Parameter values estimated against integrin binding data. The parameter estimation was run four times, or twice for each choice of peak calcium concentration assumption. For each choice, the parameter estimation was conducted under the assumption that the peak RAP1-GTP number was 500, and again under the assumption that the peak RAP1-GTP number was 5000. The estimated values assuming a peak calcium concentration of 200 nM and a peak RAP1-GTP number of 5000 were used in the results of the main paper unless otherwise stated.

## S4 Full List of Model Equations

All volume concentrations are denoted by $[\cdot]$ and all surface densities are denoted by $[\cdot]_S$. The volumes of this system are the platelet rich plasma (PRP), cytosol, and dense tubular structure (DTS), the volumes of which are denoted by $V_{\mathrm{PRP}}$, $V_{\mathrm{cyt}}$, and $V_{\mathrm{DTS}}$, respectively. The surfaces of this system are the plasma membrane and DTS membrane, the surface areas of which are denoted by $S_{\mathrm{PM}}$ and $S_{\mathrm{IM}}$, respectively.

| Parameter | Value | Units |
|---|---|---|
| $V_{\mathrm{PRP}}$ | $1 \cdot 10^{-3}$ | $\mathrm{dm}^3$ |
| $S_{\mathrm{PM}}$ | $7.35 \cdot 10^{-9}$ | $\mathrm{dm}^2$ |
| $V_{\mathrm{cyt}}$ | $6 \cdot 10^{-15}$ | $\mathrm{dm}^3$ |
| $S_{\mathrm{IM}}$ | $7.35 \cdot 10^{-8}$ | $\mathrm{dm}^2$ |
| $V_{\mathrm{DTS}}$ | $3.7 \cdot 10^{-16}$ | $\mathrm{dm}^3$ |

Tab. S3: Parameter values associated with the volumes of each container.

## S4.1 CalDAG-GEFI, RASA3, and RAP1

### S4.1.1 CalDAG-GEFI

| Parameter | Value | Units | Reference |
|---|---|---|---|
| $k_{\mathrm{Ca}}^{\mathrm{CalDAG-GEFI}}$ | $6 \cdot 10^6$ | 1/M-s | [3] |
| $k_{-\mathrm{Ca}}^{\mathrm{CalDAG-GEFI}}$ | 0.48 | 1/s | [3] |
| $N^{\mathrm{CalDAG-GEFI}}$ | 31595 | # | [17] |

Tab. S4: Parameter values associated with CalDAG-GEFI. At time $t = 0$ s in the steady-state simulation, [CalDAG] $= N^{\mathrm{CalDAG-GEFI}}/V_{\mathrm{cyt}}$ and all other states are initialized to 0.

$$V_{\mathrm{cyt}}\frac{d}{dt}[\mathrm{CalDAG-GEFI}] = \underbrace{-V_{\mathrm{cyt}}k_{\mathrm{Ca}}^{\mathrm{CalDAG-GEFI}}[\mathrm{Ca}_{\mathrm{cyt}}^{2+}][\mathrm{CalDAG-GEFI}] + V_{\mathrm{cyt}}k_{-\mathrm{Ca}}^{\mathrm{CalDAG-GEFI}}[\mathrm{CalDAG-GEFI-Ca}]}_{\text{CalDAG-GEFI binding calcium}} \tag{3}$$

$$\begin{aligned}V_{\mathrm{cyt}}\frac{d}{dt}[\mathrm{CalDAG-GEFI-Ca}] = &\underbrace{V_{\mathrm{cyt}}k_{\mathrm{Ca}}^{\mathrm{CalDAG-GEFI}}[\mathrm{Ca}_{\mathrm{cyt}}^{2+}][\mathrm{CalDAG-GEFI}] - V_{\mathrm{cyt}}k_{-\mathrm{Ca}}^{\mathrm{CalDAG-GEFI}}[\mathrm{CalDAG-GEFI-Ca}]}_{\text{CalDAG-GEFI binding calcium}}\\ &\underbrace{-V_{\mathrm{cyt}}k_{\mathrm{Ca}}^{\mathrm{CalDAG-GEFI}}[\mathrm{Ca}_{\mathrm{cyt}}^{2+}][\mathrm{CalDAG-GEFI-Ca}] + V_{\mathrm{cyt}}k_{-\mathrm{Ca}}^{\mathrm{CalDAG-GEFI}}[\mathrm{CalDAG-GEFI-2Ca}]}_{\text{CalDAG-GEFI-Ca binding calcium}}\end{aligned} \tag{4}$$

$$V_{\mathrm{cyt}}\frac{d}{dt}[\mathrm{CalDAG-GEFI-2Ca}] = \underbrace{V_{\mathrm{cyt}}k_{\mathrm{Ca}}^{\mathrm{CalDAG-GEFI}}[\mathrm{Ca}_{\mathrm{cyt}}^{2+}][\mathrm{CalDAG-GEFI-Ca}] - V_{\mathrm{cyt}}k_{-\mathrm{Ca}}^{\mathrm{CalDAG-GEFI}}[\mathrm{CalDAG-GEFI-2Ca}]}_{\text{CalDAG-GEFI-Ca binding calcium}} \tag{5}$$

### S4.1.2 RASA3

RASA3 inactivation is not well known experimentally, so for now we will treat it as a mass-action binding between RASA3 and $PIP_3$.

| Parameter | Value | Units | Reference |
|---|---|---|---|
| $k_{PIP_3}^{RASA3}$ | See Table S2 | 1/M-s | |
| $k_{-PIP_3}^{RASA3}$ | See Table S2 | 1/s | |
| $N^{RASA3}$ | 26959 | # | [17] |

Tab. S5: Parameter values associated with RASA3. At time $t = 0$ s in the steady-state simulation, $[\mathrm{RASA3}]_S = N^{\mathrm{RASA3}}/S_{\mathrm{PM}}$ and all other states are initialized to 0.

$$S_{\mathrm{PM}}\frac{d}{dt}[\mathrm{RASA3}]_S = \underbrace{S_{\mathrm{PM}}k_{-\mathrm{PIP}_3}^{\mathrm{RASA3}}[\mathrm{RASA3}-\mathrm{PIP}_3]_S - S_{\mathrm{PM}}\left(\frac{S_{\mathrm{PM}}}{V_{\mathrm{cyt}}}k_{\mathrm{PIP}_3}^{\mathrm{RASA3}}\right)[\mathrm{RASA3}]_S[\mathrm{PIP}_3]_S}_{\mathrm{PIP}_3\text{ inactivation of RASA3}} \tag{6}$$

$$S_{\mathrm{PM}}\frac{d}{dt}[\mathrm{RASA3}-\mathrm{PIP}_3]_S = \underbrace{-S_{\mathrm{PM}}k_{-\mathrm{PIP}_3}^{\mathrm{RASA3}}[\mathrm{RASA3}-\mathrm{PIP}_3]_S + S_{\mathrm{PM}}\left(\frac{S_{\mathrm{PM}}}{V_{\mathrm{cyt}}}k_{\mathrm{PIP}_3}^{\mathrm{RASA3}}\right)[\mathrm{RASA3}]_S[\mathrm{PIP}_3]_S}_{\mathrm{PIP}_3\text{ inactivation of RASA3}} \tag{7}$$

### S4.1.3 RAP1

| Parameter | Value | Units | Reference |
|---|---|---|---|
| $k_{\text{cat}}^{\text{GAP}}$ | See Table S2 | 1/s | |
| $K_{\text{M}}^{\text{GAP}}$ | See Table S2 | M | |
| $k_{\text{cat}}^{\text{GEF}}$ | See Table S2 | 1/s | |
| $K_{\text{M}}^{\text{GAP}}$ | See Table S2 | M | |
| $N^{\text{RAP1}}$ | 209601 | # | [17] |

Tab. S6: Parameter values associated with RAP1. At time $t = 0$ s in the steady-state simulation, $[\text{RAP1}-\text{GDP}]_S = N^{\text{RAP1}}/S_{\text{PM}}$ and all other states are initialized to 0.

$$S_{\text{PM}}\frac{d}{dt}[\text{RAP1}-\text{GTP}]_S = \underbrace{-S_{\text{PM}}\frac{k_{\text{cat}}^{\text{GAP}}[\text{RASA3}]_S}{\left(\frac{V_{\text{cyt}}}{S_{\text{PM}}}K_{\text{M}}^{\text{GAP}}\right)+[\text{RAP1}-\text{GTP}]_S}[\text{RAP1}-\text{GTP}]_S}_{\text{RASA3 inactivation of RAP1}} \\ \underbrace{+V_{\text{cyt}}\frac{k_{\text{cat}}^{\text{GEF}}[\text{CalDAG}-\text{GEFI}-2\text{Ca}]}{\left(\frac{V_{\text{cyt}}}{S_{\text{PM}}}K_{\text{M}}^{\text{GEF}}\right)+[\text{RAP1}-\text{GDP}]_S}[\text{RAP1}-\text{GDP}]_S}_{\text{CalDAG activation of RAP1}} \tag{8}$$

$$S_{\text{PM}}\frac{d}{dt}[\text{RAP1}-\text{GDP}]_S = \underbrace{S_{\text{PM}}\frac{k_{\text{cat}}^{\text{GAP}}[\text{RASA3}]_S}{\left(\frac{V_{\text{cyt}}}{S_{\text{PM}}}K_{\text{M}}^{\text{GAP}}\right)+[\text{RAP1}-\text{GTP}]_S}[\text{RAP1}-\text{GTP}]_S}_{\text{RASA3 inactivation of RAP1}} \\ \underbrace{-V_{\text{cyt}}\frac{k_{\text{cat}}^{\text{GEF}}[\text{CalDAG}-\text{GEFI}-2\text{Ca}]}{\left(\frac{V_{\text{cyt}}}{S_{\text{PM}}}K_{\text{M}}^{\text{GEF}}\right)+[\text{RAP1}-\text{GDP}]_S}[\text{RAP1}-\text{GDP}]_S}_{\text{CalDAG activation of RAP1}} \tag{9}$$

## S4.2 ADP

$$
\begin{aligned}
V_{\mathrm{PRP}}\frac{d}{dt}[\mathrm{ADP}] = {} & \underbrace{-S_{\mathrm{PM}}\left(k_{\mathrm{ADP}}^{\mathrm{P2Y_{12}}}\frac{\alpha^{\mathrm{P2Y_{12}}}K_{\mathrm{act}}^{\mathrm{P2Y_{12}}}+1}{K_{\mathrm{act}}^{\mathrm{P2Y_{12}}}+1}\right)[\mathrm{ADP}][\mathrm{P2Y_{12}}]_S + S_{\mathrm{PM}}k_{-\mathrm{ADP}}^{\mathrm{P2Y_{12}}}[\mathrm{ADP}-\mathrm{P2Y_{12}}]_S}_{\text{ADP Binding to P2Y}_{12}} \\
& \underbrace{\begin{aligned} & - S_{\mathrm{PM}}\left(k_{\mathrm{ADP}}^{\mathrm{P2Y_{12}}}\frac{\alpha^{\mathrm{P2Y_{12}}}\beta^{\mathrm{P2Y_{12}}}K_{\mathrm{act}}^{\mathrm{P2Y_{12}}}+1}{\beta^{\mathrm{P2Y_{12}}}K_{\mathrm{act}}^{\mathrm{P2Y_{12}}}+1}\right)[\mathrm{ADP}][\mathrm{P2Y_{12}}-\mathrm{G_iGDP}]_S \\ & \quad +S_{\mathrm{PM}}\left(\frac{k_{-\mathrm{ADP}}^{\mathrm{P2Y_{12}}}}{\gamma^{\mathrm{P2Y_{12}}}}\frac{\alpha^{\mathrm{P2Y_{12}}}\beta^{\mathrm{P2Y_{12}}}K_{\mathrm{act}}^{\mathrm{P2Y_{12}}}+1}{\alpha^{\mathrm{P2Y_{12}}}\beta^{\mathrm{P2Y_{12}}}\delta^{\mathrm{P2Y_{12}}}K_{\mathrm{act}}^{\mathrm{P2Y_{12}}}+1}\right)[\mathrm{ADP}-\mathrm{P2Y_{12}}-\mathrm{G_iGDP}]_S \end{aligned}}_{\text{ADP Binding to P2Y}_{12}\text{-G}_i\text{GDP}} \\
& \underbrace{-S_{\mathrm{PM}}\left(k_{\mathrm{ADP}}^{\mathrm{P2Y_{1}}}\frac{\alpha^{\mathrm{P2Y_{1}}}K_{\mathrm{act}}^{\mathrm{P2Y_{1}}}+1}{K_{\mathrm{act}}^{\mathrm{P2Y_{1}}}+1}\right)[\mathrm{ADP}][\mathrm{P2Y_{1}}]_S + S_{\mathrm{PM}}k_{-\mathrm{ADP}}^{\mathrm{P2Y_{1}}}[\mathrm{ADP}-\mathrm{P2Y_{1}}]_S}_{\text{ADP Binding to P2Y}_{1}} \\
& \underbrace{\begin{aligned} & - S_{\mathrm{PM}}\left(k_{\mathrm{ADP}}^{\mathrm{P2Y_{1}}}\frac{\alpha^{\mathrm{P2Y_{1}}}\beta^{\mathrm{P2Y_{1}}}K_{\mathrm{act}}^{\mathrm{P2Y_{1}}}+1}{\beta^{\mathrm{P2Y_{1}}}K_{\mathrm{act}}^{\mathrm{P2Y_{1}}}+1}\right)[\mathrm{ADP}][\mathrm{P2Y_{1}}-\mathrm{G_qGDP}]_S \\ & \quad +S_{\mathrm{PM}}\left(\frac{k_{-\mathrm{ADP}}^{\mathrm{P2Y_{1}}}}{\gamma^{\mathrm{P2Y_{1}}}}\frac{\alpha^{\mathrm{P2Y_{1}}}\beta^{\mathrm{P2Y_{1}}}K_{\mathrm{act}}^{\mathrm{P2Y_{1}}}+1}{\alpha^{\mathrm{P2Y_{1}}}\beta^{\mathrm{P2Y_{1}}}\delta^{\mathrm{P2Y_{1}}}K_{\mathrm{act}}^{\mathrm{P2Y_{1}}}+1}\right)[\mathrm{ADP}-\mathrm{P2Y_{1}}-\mathrm{G_qGDP}]_S \end{aligned}}_{\text{ADP Binding to P2Y}_{1}\text{-G}_q\text{GDP}}
\end{aligned} \tag{10}
$$

## S4.3 Proteins in $P2Y_{12}$ Pathway

### S4.3.1 $P2Y_{12}$

The $P2Y_{12}$ model is exactly the same as what Purvis et al. used for $P2Y_1$ [12]. However, we performed a Quasi-Steady State reduction to reduce the number of equations by four. See later in the supplemental for further discussion.

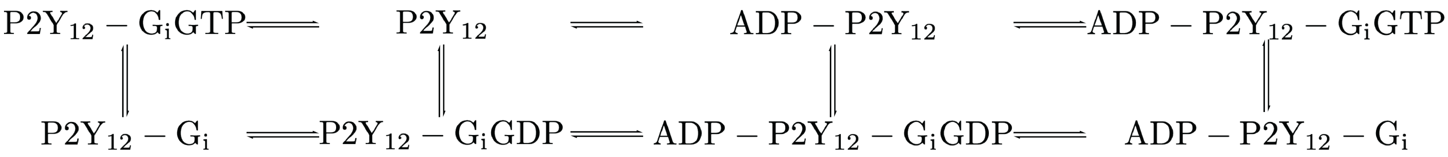


Fig. S1: $P2Y_{12}$ reaction diagram

| Parameter | Value | Units | Reference |
|---|---|---|---|
| $K_{act}^{P2Y_{12}}$ | See Table S1 | n.d. | |
| $\alpha^{P2Y_{12}}$ | See Table S1 | n.d. | |
| $\beta^{P2Y_{12}}$ | See Table S1 | n.d. | |
| $\delta^{P2Y_{12}}$ | See Table S1 | n.d. | |
| $\gamma^{P2Y_{12}}$ | See Table S1 | n.d. | |
| $k_{G_iGDP}^{P2Y_{12}}$ | See Table S1 | $dm^2$/mol-s | |
| $k_{-G_iGDP}^{P2Y_{12}}$ | See Table S1 | 1/s | |
| $k_{G_iGTP}^{P2Y_{12}}$ | See Table S1 | $dm^2$/mol-s | |
| $k_{-G_iGTP}^{P2Y_{12}}$ | See Table S1 | 1/s | |
| $k_{ADP}^{P2Y_{12}}$ | See Table S1 | 1/M-s | |
| $k_{-ADP}^{P2Y_{12}}$ | See Table S1 | 1/s | |
| $k_{-GDP}^{P2Y_{12}}$ | See Table S1 | 1/s | |
| $k_{GDP}^{P2Y_{12}}$ | See Table S1 | 1/M-s | |
| $k_{-GTP}^{P2Y_{12}}$ | See Table S1 | 1/s | |
| $k_{GTP}^{P2Y_{12}}$ | See Table S1 | 1/M-s | |
| [GDP] | $1.3362 \cdot 10^{-5}$ | M | [12] |
| [GTP] | $7.117 \cdot 10^{-4}$ | M | [12] |
| $N^{P2Y_{12}}$ | 2681 | # | [17] |

Tab. S7: Parameter values associated with $P2Y_{12}$. At time At time $t = 0$ s in the steady-state simulation, $[P2Y_{12}]_S = N^{P2Y_{12}}/S_{PM}$ and all other states are initialized to 0.

$$
\begin{aligned}
S_{\mathrm{PM}}\frac{d}{dt}[\mathrm{P2Y_{12}}]_S = {} & \underbrace{-S_{\mathrm{PM}}\left(k_{\mathrm{ADP}}^{\mathrm{P2Y_{12}}}\frac{\alpha^{\mathrm{P2Y_{12}}}K_{\mathrm{act}}^{\mathrm{P2Y_{12}}}+1}{K_{\mathrm{act}}^{\mathrm{P2Y_{12}}}+1}\right)[\mathrm{ADP}][\mathrm{P2Y_{12}}]_S + S_{\mathrm{PM}}k_{-\mathrm{ADP}}^{\mathrm{P2Y_{12}}}[\mathrm{ADP}-\mathrm{P2Y_{12}}]_S}_{\text{ADP Binding}} \\
& \underbrace{\begin{aligned} & -S_{\mathrm{PM}}\left(k_{\mathrm{G_iGDP}}^{\mathrm{P2Y_{12}}}\frac{\beta^{\mathrm{P2Y_{12}}}K_{\mathrm{act}}^{\mathrm{P2Y_{12}}}}{K_{\mathrm{act}}^{\mathrm{P2Y_{12}}}+1}\right)[\mathrm{G_iGDP}]_S[\mathrm{P2Y_{12}}]_S \\ & \quad +S_{\mathrm{PM}}\left(k_{-\mathrm{G_iGDP}}^{\mathrm{P2Y_{12}}}\frac{\beta^{\mathrm{P2Y_{12}}}K_{\mathrm{act}}^{\mathrm{P2Y_{12}}}}{\beta^{\mathrm{P2Y_{12}}}K_{\mathrm{act}}^{\mathrm{P2Y_{12}}}+1}\right)[\mathrm{P2Y_{12}}-\mathrm{G_iGDP}]_S \end{aligned}}_{\mathrm{G}_i\text{GDP Binding}} \\
& \underbrace{\begin{aligned} & -S_{\mathrm{PM}}\left(k_{\mathrm{G_iGTP}}^{\mathrm{P2Y_{12}}}\frac{K_{\mathrm{act}}^{\mathrm{P2Y_{12}}}}{K_{\mathrm{act}}^{\mathrm{P2Y_{12}}}+1}\right)[\mathrm{G_iGTP}]_S[\mathrm{P2Y_{12}}]_S \\ & \quad +S_{\mathrm{PM}}k_{-\mathrm{G_iGTP}}^{\mathrm{P2Y_{12}}}[\mathrm{P2Y_{12}}-\mathrm{G_iGTP}]_S \end{aligned}}_{\mathrm{G}_i\text{GTP Binding}}
\end{aligned}
\tag{11}
$$

$$
\begin{aligned}
S_{\mathrm{PM}}\frac{d}{dt}[\mathrm{ADP}-\mathrm{P2Y_{12}}]_S = {} & \underbrace{S_{\mathrm{PM}}\left(k_{\mathrm{ADP}}^{\mathrm{P2Y_{12}}}\frac{\alpha^{\mathrm{P2Y_{12}}}K_{\mathrm{act}}^{\mathrm{P2Y_{12}}}+1}{K_{\mathrm{act}}^{\mathrm{P2Y_{12}}}+1}\right)[\mathrm{ADP}][\mathrm{P2Y_{12}}]_S - S_{\mathrm{PM}}k_{-\mathrm{ADP}}^{\mathrm{P2Y_{12}}}[\mathrm{ADP}-\mathrm{P2Y_{12}}]_S}_{\text{ADP Binding}} \\
& \underbrace{\begin{aligned} & -S_{\mathrm{PM}}\left(k_{\mathrm{G_iGDP}}^{\mathrm{P2Y_{12}}}\frac{\alpha^{\mathrm{P2Y_{12}}}\beta^{\mathrm{P2Y_{12}}}K_{\mathrm{act}}^{\mathrm{P2Y_{12}}}}{\alpha^{\mathrm{P2Y_{12}}}K_{\mathrm{act}}^{\mathrm{P2Y_{12}}}+1}\right)[\mathrm{G_iGDP}]_S[\mathrm{ADP}-\mathrm{P2Y_{12}}]_S \\ & \quad +S_{\mathrm{PM}}\left(\frac{k_{-\mathrm{G_iGDP}}^{\mathrm{P2Y_{12}}}}{\gamma^{\mathrm{P2Y_{12}}}}\frac{\alpha^{\mathrm{P2Y_{12}}}\beta^{\mathrm{P2Y_{12}}}K_{\mathrm{act}}^{\mathrm{P2Y_{12}}}}{\alpha^{\mathrm{P2Y_{12}}}\beta^{\mathrm{P2Y_{12}}}\delta^{\mathrm{P2Y_{12}}}K_{\mathrm{act}}^{\mathrm{P2Y_{12}}}+1}\right)[\mathrm{ADP}-\mathrm{P2Y_{12}}-\mathrm{G_iGDP}]_S \end{aligned}}_{\mathrm{G}_i\text{GDP Binding}} \\
& \underbrace{\begin{aligned} & -S_{\mathrm{PM}}\left(k_{\mathrm{G_iGTP}}^{\mathrm{P2Y_{12}}}\frac{\alpha^{\mathrm{P2Y_{12}}}K_{\mathrm{act}}^{\mathrm{P2Y_{12}}}}{\alpha^{\mathrm{P2Y_{12}}}K_{\mathrm{act}}^{\mathrm{P2Y_{12}}}+1}\right)[\mathrm{G_iGTP}]_S[\mathrm{ADP}-\mathrm{P2Y_{12}}]_S \\ & \quad +S_{\mathrm{PM}}k_{-\mathrm{G_iGTP}}^{\mathrm{P2Y_{12}}}[\mathrm{ADP}-\mathrm{P2Y_{12}}-\mathrm{G_iGTP}]_S \end{aligned}}_{\mathrm{G}_i\text{GTP Binding}}
\end{aligned}
\tag{12}
$$

$$
\begin{aligned}
S_{\mathrm{PM}}\frac{d}{dt}[\mathrm{P2Y_{12}}-\mathrm{G_iGDP}]_S = {} & \underbrace{\begin{aligned} & -S_{\mathrm{PM}}\left(k_{\mathrm{ADP}}^{\mathrm{P2Y_{12}}}\frac{\alpha^{\mathrm{P2Y_{12}}}\beta^{\mathrm{P2Y_{12}}}K_{\mathrm{act}}^{\mathrm{P2Y_{12}}}+1}{\beta^{\mathrm{P2Y_{12}}}K_{\mathrm{act}}^{\mathrm{P2Y_{12}}}+1}\right)[\mathrm{ADP}][\mathrm{P2Y_{12}}-\mathrm{G_iGDP}]_S \\ & \quad +S_{\mathrm{PM}}\left(\frac{k_{-\mathrm{ADP}}^{\mathrm{P2Y_{12}}}}{\gamma^{\mathrm{P2Y_{12}}}}\frac{\alpha^{\mathrm{P2Y_{12}}}\beta^{\mathrm{P2Y_{12}}}K_{\mathrm{act}}^{\mathrm{P2Y_{12}}}+1}{\alpha^{\mathrm{P2Y_{12}}}\beta^{\mathrm{P2Y_{12}}}\delta^{\mathrm{P2Y_{12}}}K_{\mathrm{act}}^{\mathrm{P2Y_{12}}}+1}\right)[\mathrm{ADP}-\mathrm{P2Y_{12}}-\mathrm{G_iGDP}]_S \end{aligned}}_{\text{ADP Binding}} \\
& \underbrace{\begin{aligned} & +S_{\mathrm{PM}}\left(k_{\mathrm{G_iGDP}}^{\mathrm{P2Y_{12}}}\frac{\beta^{\mathrm{P2Y_{12}}}K_{\mathrm{act}}^{\mathrm{P2Y_{12}}}}{K_{\mathrm{act}}^{\mathrm{P2Y_{12}}}+1}\right)[\mathrm{G_iGDP}]_S[\mathrm{P2Y_{12}}]_S \\ & \quad -S_{\mathrm{PM}}\left(k_{-\mathrm{G_iGDP}}^{\mathrm{P2Y_{12}}}\frac{\beta^{\mathrm{P2Y_{12}}}K_{\mathrm{act}}^{\mathrm{P2Y_{12}}}}{\beta^{\mathrm{P2Y_{12}}}K_{\mathrm{act}}^{\mathrm{P2Y_{12}}}+1}\right)[\mathrm{P2Y_{12}}-\mathrm{G_iGDP}]_S \end{aligned}}_{\mathrm{G}_i\text{GDP Binding}} \\
& \underbrace{\begin{aligned} & +S_{\mathrm{PM}}k_{\mathrm{GDP}}^{\mathrm{P2Y_{12}}}[\mathrm{GDP}]_S[\mathrm{P2Y_{12}}-\mathrm{G_i}]_S \\ & \quad -S_{\mathrm{PM}}\left(k_{-\mathrm{GDP}}^{\mathrm{P2Y_{12}}}\frac{\beta^{\mathrm{P2Y_{12}}}K_{\mathrm{act}}^{\mathrm{P2Y_{12}}}}{\beta^{\mathrm{P2Y_{12}}}K_{\mathrm{act}}^{\mathrm{P2Y_{12}}}+1}\right)[\mathrm{P2Y_{12}}-\mathrm{G_iGDP}]_S \end{aligned}}_{\text{GDP Binding}}
\end{aligned}
\tag{13}
$$

$$S_{\mathrm{PM}}\frac{d}{dt}[\mathrm{ADP}-\mathrm{P2Y_{12}}-\mathrm{G_iGDP}]_S = \underbrace{S_{\mathrm{PM}}\left(k_{\mathrm{ADP}}^{\mathrm{P2Y_{12}}}\frac{\alpha^{\mathrm{P2Y_{12}}}\beta^{\mathrm{P2Y_{12}}}K_{\mathrm{act}}^{\mathrm{P2Y_{12}}}+1}{\beta^{\mathrm{P2Y_{12}}}K_{\mathrm{act}}^{\mathrm{P2Y_{12}}}+1}\right)[\mathrm{ADP}][\mathrm{P2Y_{12}}-\mathrm{G_iGDP}]_S - S_{\mathrm{PM}}\left(\frac{k_{-\mathrm{ADP}}^{\mathrm{P2Y_{12}}}}{\gamma^{\mathrm{P2Y_{12}}}}\frac{\alpha^{\mathrm{P2Y_{12}}}\beta^{\mathrm{P2Y_{12}}}K_{\mathrm{act}}^{\mathrm{P2Y_{12}}}+1}{\alpha^{\mathrm{P2Y_{12}}}\beta^{\mathrm{P2Y_{12}}}\delta^{\mathrm{P2Y_{12}}}K_{\mathrm{act}}^{\mathrm{P2Y_{12}}}+1}\right)[\mathrm{ADP}-\mathrm{P2Y_{12}}-\mathrm{G_iGDP}]_S}_{\text{ADP Binding}}$$
$$+\underbrace{S_{\mathrm{PM}}\left(k_{\mathrm{G_iGDP}}^{\mathrm{P2Y_{12}}}\frac{\alpha^{\mathrm{P2Y_{12}}}\beta^{\mathrm{P2Y_{12}}}K_{\mathrm{act}}^{\mathrm{P2Y_{12}}}}{\alpha^{\mathrm{P2Y_{12}}}K_{\mathrm{act}}^{\mathrm{P2Y_{12}}}+1}\right)[\mathrm{G_iGDP}]_S[\mathrm{ADP}-\mathrm{P2Y_{12}}]_S - S_{\mathrm{PM}}\left(\frac{k_{-\mathrm{G_iGDP}}^{\mathrm{P2Y_{12}}}}{\gamma^{\mathrm{P2Y_{12}}}}\frac{\alpha^{\mathrm{P2Y_{12}}}\beta^{\mathrm{P2Y_{12}}}K_{\mathrm{act}}^{\mathrm{P2Y_{12}}}}{\alpha^{\mathrm{P2Y_{12}}}\beta^{\mathrm{P2Y_{12}}}\delta^{\mathrm{P2Y_{12}}}K_{\mathrm{act}}^{\mathrm{P2Y_{12}}}+1}\right)[\mathrm{ADP}-\mathrm{P2Y_{12}}-\mathrm{G_iGDP}]_S}_{\mathrm{G}_i\text{GDP Binding}}$$
$$+\underbrace{S_{\mathrm{PM}}k_{\mathrm{GDP}}^{\mathrm{P2Y_{12}}}[\mathrm{GDP}][\mathrm{ADP}-\mathrm{P2Y_{12}}-\mathrm{G_i}]_S - S_{\mathrm{PM}}\left(k_{-\mathrm{GDP}}^{\mathrm{P2Y_{12}}}\frac{\alpha^{\mathrm{P2Y_{12}}}\beta^{\mathrm{P2Y_{12}}}\delta^{\mathrm{P2Y_{12}}}K_{\mathrm{act}}^{\mathrm{P2Y_{12}}}}{\alpha^{\mathrm{P2Y_{12}}}\beta^{\mathrm{P2Y_{12}}}\delta^{\mathrm{P2Y_{12}}}K_{\mathrm{act}}^{\mathrm{P2Y_{12}}}+1}\right)[\mathrm{ADP}-\mathrm{P2Y_{12}}-\mathrm{G_iGDP}]_S}_{\text{GDP Binding}} \tag{14}$$

$$S_{\mathrm{PM}}\frac{d}{dt}[\mathrm{P2Y_{12}}-\mathrm{G_iGTP}]_S = \underbrace{S_{\mathrm{PM}}k_{\mathrm{GTP}}^{\mathrm{P2Y_{12}}}[\mathrm{P2Y_{12}}-\mathrm{G_i}]_S[\mathrm{GTP}] - S_{\mathrm{PM}}k_{-\mathrm{GTP}}^{\mathrm{P2Y_{12}}}[\mathrm{P2Y_{12}}-\mathrm{G_iGTP}]_S}_{\text{GTP Binding}}$$
$$+\underbrace{S_{\mathrm{PM}}\left(k_{\mathrm{G_iGTP}}^{\mathrm{P2Y_{12}}}\frac{K_{\mathrm{act}}^{\mathrm{P2Y_{12}}}}{K_{\mathrm{act}}^{\mathrm{P2Y_{12}}}+1}\right)[\mathrm{G_iGTP}]_S[\mathrm{P2Y_{12}}]_S - S_{\mathrm{PM}}k_{-\mathrm{G_iGTP}}^{\mathrm{P2Y_{12}}}[\mathrm{P2Y_{12}}-\mathrm{G_iGTP}]_S}_{\mathrm{G}_i\text{GTP binding}} \tag{15}$$

$$S_{\mathrm{PM}}\frac{d}{dt}[\mathrm{ADP}-\mathrm{P2Y_{12}}-\mathrm{G_iGTP}]_S = \underbrace{S_{\mathrm{PM}}k_{\mathrm{GTP}}^{\mathrm{P2Y_{12}}}[\mathrm{ADP}-\mathrm{P2Y_{12}}-\mathrm{G_i}]_S[\mathrm{GTP}] - S_{\mathrm{PM}}k_{-\mathrm{GTP}}^{\mathrm{P2Y_{12}}}[\mathrm{ADP}-\mathrm{P2Y_{12}}-\mathrm{G_iGTP}]_S}_{\text{GTP Binding}}$$
$$+\underbrace{S_{\mathrm{PM}}\left(k_{\mathrm{G_iGTP}}^{\mathrm{P2Y_{12}}}\frac{\alpha^{\mathrm{P2Y_{12}}}K_{\mathrm{act}}^{\mathrm{P2Y_{12}}}}{\alpha^{\mathrm{P2Y_{12}}}K_{\mathrm{act}}^{\mathrm{P2Y_{12}}}+1}\right)[\mathrm{G_iGTP}]_S[\mathrm{ADP}-\mathrm{P2Y_{12}}]_S - S_{\mathrm{PM}}k_{-\mathrm{G_iGTP}}^{\mathrm{P2Y_{12}}}[\mathrm{ADP}-\mathrm{P2Y_{12}}-\mathrm{G_iGTP}]_S}_{\mathrm{G}_i\text{GTP binding}} \tag{16}$$

$$
\begin{aligned}
S_{\mathrm{PM}}\frac{d}{dt}[\mathrm{P2Y_{12}-G_i}]_S = &\underbrace{-S_{\mathrm{PM}}k_{\mathrm{GTP}}^{\mathrm{P2Y_{12}}}[\mathrm{P2Y_{12}-G_i}]_S[\mathrm{GTP}] + S_{\mathrm{PM}}k_{\mathrm{-GTP}}^{\mathrm{P2Y_{12}}}[\mathrm{P2Y_{12}-G_iGTP}]_S}_{\text{GTP Binding}} \\
&\underbrace{+ S_{\mathrm{PM}}\left(k_{\mathrm{-GDP}}^{\mathrm{P2Y_{12}}}\frac{\beta^{\mathrm{P2Y_{12}}}K_{\mathrm{act}}^{\mathrm{P2Y_{12}}}}{\beta^{\mathrm{P2Y_{12}}}K_{\mathrm{act}}^{\mathrm{P2Y_{12}}}+1}\right)[\mathrm{P2Y_{12}-G_iGDP}]_S - S_{\mathrm{PM}}k_{\mathrm{GDP}}^{\mathrm{P2Y_{12}}}[\mathrm{P2Y_{12}-G_i}]_S[\mathrm{GDP}]}_{\text{GDP binding}}
\end{aligned}
\tag{17}
$$

$$
\begin{aligned}
S_{\mathrm{PM}}\frac{d}{dt}[\mathrm{ADP-P2Y_{12}-G_i}]_S = &\underbrace{-S_{\mathrm{PM}}k_{\mathrm{GTP}}^{\mathrm{P2Y_{12}}}[\mathrm{ADP-P2Y_{12}-G_i}]_S[\mathrm{GTP}] + S_{\mathrm{PM}}k_{\mathrm{-GTP}}^{\mathrm{P2Y_{12}}}[\mathrm{ADP-P2Y_{12}-G_iGTP}]_S}_{\text{GTP Binding}} \\
&\underbrace{+ S_{\mathrm{PM}}\left(k_{\mathrm{-GDP}}^{\mathrm{P2Y_{12}}}\frac{\alpha^{\mathrm{P2Y_{12}}}\beta^{\mathrm{P2Y_{12}}}\delta^{\mathrm{P2Y_{12}}}K_{\mathrm{act}}^{\mathrm{P2Y_{12}}}}{\alpha^{\mathrm{P2Y_{12}}}\beta^{\mathrm{P2Y_{12}}}\delta^{\mathrm{P2Y_{12}}}K_{\mathrm{act}}^{\mathrm{P2Y_{12}}}+1}\right)[\mathrm{ADP-P2Y_{12}-G_iGDP}]_S - S_{\mathrm{PM}}k_{\mathrm{GDP}}^{\mathrm{P2Y_{12}}}[\mathrm{ADP-P2Y_{12}-G_i}]_S[\mathrm{GDP}]}_{\text{GDP binding}}
\end{aligned}
\tag{18}
$$

### S4.3.2 G$_i$

| Parameter | Value | Units | Reference |
|---|---|---|---|
| $k_{\mathrm{GTP}}$ | See Table S1 | 1/s | |
| $k^{\mathrm{G_i}}_{\mathrm{G_{i\beta\gamma}}}$ | 103584 | 1/M-s | [7] |
| $k^{\mathrm{G_i}}_{-\mathrm{G_{i\beta\gamma}}}$ | 7.7832 | 1/s | [7] |
| $N^{\mathrm{G_i}}$ | 27752 | # | [17] |

Tab. S8: Parameter values associated with $\mathrm{G_i}$. At time $t = 0$ s in the steady-state simulation, $[\mathrm{G_iGDP}]_S = N^{\mathrm{G_i}}/S_{\mathrm{PM}}$ and all other states are initialized to 0.

$$\begin{aligned} S_{\mathrm{PM}}\frac{d}{dt}[\mathrm{G_iGDP}]_S = & \underbrace{-S_{\mathrm{PM}}\left(k^{\mathrm{P2Y_{12}}}_{\mathrm{G_iGDP}}\frac{\beta^{\mathrm{P2Y_{12}}}K^{\mathrm{P2Y_{12}}}_{\mathrm{act}}}{K^{\mathrm{P2Y_{12}}}_{\mathrm{act}}+1}\right)[\mathrm{G_iGDP}]_S[\mathrm{P2Y_{12}}]_S + S_{\mathrm{PM}}\left(k^{\mathrm{P2Y_{12}}}_{-\mathrm{G_iGDP}}\frac{\beta^{\mathrm{P2Y_{12}}}K^{\mathrm{P2Y_{12}}}_{\mathrm{act}}}{\beta^{\mathrm{P2Y_{12}}}K^{\mathrm{P2Y_{12}}}_{\mathrm{act}}+1}\right)[\mathrm{P2Y_{12}-G_iGDP}]_S}_{\mathrm{G}_i\mathrm{GDP\ Binding}} \\ & \underbrace{-S_{\mathrm{PM}}\left(k^{\mathrm{P2Y_{12}}}_{\mathrm{G_iGDP}}\frac{\alpha^{\mathrm{P2Y_{12}}}\beta^{\mathrm{P2Y_{12}}}K^{\mathrm{P2Y_{12}}}_{\mathrm{act}}}{\alpha^{\mathrm{P2Y_{12}}}K^{\mathrm{P2Y_{12}}}_{\mathrm{act}}+1}\right)[\mathrm{G_iGDP}]_S[\mathrm{ADP-P2Y_{12}}]_S + S_{\mathrm{PM}}\left(\frac{k^{\mathrm{P2Y_{12}}}_{-\mathrm{G_iGDP}}}{\gamma^{\mathrm{P2Y_{12}}}}\frac{\alpha^{\mathrm{P2Y_{12}}}\beta^{\mathrm{P2Y_{12}}}K^{\mathrm{P2Y_{12}}}_{\mathrm{act}}}{\alpha^{\mathrm{P2Y_{12}}}\beta^{\mathrm{P2Y_{12}}}\delta^{\mathrm{P2Y_{12}}}K^{\mathrm{P2Y_{12}}}_{\mathrm{act}}+1}\right)[\mathrm{ADP-P2Y_{12}-G_iGDP}]_S}_{\mathrm{G}_i\mathrm{GDP\ Binding}} \\ & \underbrace{+S_{\mathrm{PM}}\left(\frac{S_{\mathrm{PM}}}{V_{\mathrm{cyt}}}k^{\mathrm{G_i}}_{\mathrm{G_{i\beta\gamma}}}\right)[\mathrm{G_{i\alpha}GDP}]_S[\mathrm{G_{i\beta\gamma}}]_S - S_{\mathrm{PM}}k^{\mathrm{G_i}}_{-\mathrm{G_{i\beta\gamma}}}[\mathrm{G_iGDP}]_S}_{\mathrm{G}_{i\beta\gamma}\text{ binding }\mathrm{G}_{i\alpha}\mathrm{GDP}} \end{aligned} \tag{19}$$

$$\begin{aligned} S_{\mathrm{PM}}\frac{d}{dt}[\mathrm{G_iGTP}]_S = & \underbrace{-S_{\mathrm{PM}}\left(k^{\mathrm{P2Y_{12}}}_{\mathrm{G_iGTP}}\frac{K^{\mathrm{P2Y_{12}}}_{\mathrm{act}}}{K^{\mathrm{P2Y_{12}}}_{\mathrm{act}}+1}\right)[\mathrm{G_iGTP}]_S[\mathrm{P2Y_{12}}]_S + S_{\mathrm{PM}}k^{\mathrm{P2Y_{12}}}_{-\mathrm{G_iGTP}}[\mathrm{P2Y_{12}-G_iGTP}]_S}_{\mathrm{G}_i\mathrm{GTP\ Binding}} \\ & \underbrace{-S_{\mathrm{PM}}\left(k^{\mathrm{P2Y_{12}}}_{\mathrm{G_iGTP}}\frac{\alpha^{\mathrm{P2Y_{12}}}K^{\mathrm{P2Y_{12}}}_{\mathrm{act}}}{\alpha^{\mathrm{P2Y_{12}}}K^{\mathrm{P2Y_{12}}}_{\mathrm{act}}+1}\right)[\mathrm{G_iGTP}]_S[\mathrm{ADP-P2Y_{12}}]_S + S_{\mathrm{PM}}k^{\mathrm{P2Y_{12}}}_{-\mathrm{G_iGTP}}[\mathrm{ADP-P2Y_{12}-G_iGTP}]_S}_{\mathrm{G}_i\mathrm{GTP\ Binding}} \\ & \underbrace{-S_{\mathrm{PM}}k^{\mathrm{G_i}}_{\mathrm{autohydrolysis}}[\mathrm{G_iGTP}]_S}_{\text{Autohydrolysis of }\mathrm{G}_i\mathrm{GTP}} \\ & \underbrace{+S_{\mathrm{PM}}\left(\frac{S_{\mathrm{PM}}}{V_{\mathrm{cyt}}}k^{\mathrm{G_i}}_{\mathrm{G_{i\beta\gamma}}}\right)[\mathrm{G_{i\alpha}GTP}]_S[\mathrm{G_{i\beta\gamma}}]_S - S_{\mathrm{PM}}k^{\mathrm{G_i}}_{-\mathrm{G_{i\beta\gamma}}}[\mathrm{G_iGTP}]_S}_{\mathrm{G}_{i\beta\gamma}\text{ binding }\mathrm{G}_{i\alpha}\mathrm{GTP}} \end{aligned} \tag{20}$$

$$S_{\mathrm{PM}}\frac{d}{dt}[\mathrm{G_{i\alpha}GTP}]_S = \underbrace{-S_{\mathrm{PM}}\left(k^{\mathrm{G_i}}_{\mathrm{G_{i\beta\gamma}}}\frac{S_{\mathrm{PM}}}{V_{\mathrm{cyt}}}\right)[\mathrm{G_{i\alpha}GTP}]_S[\mathrm{G_{i\beta\gamma}}]_S + S_{\mathrm{PM}}k^{\mathrm{G_i}}_{-\mathrm{G_{i\beta\gamma}}}[\mathrm{G_iGTP}]_S}_{\mathrm{G}_{i\beta\gamma}\text{ binding }\mathrm{G}_{i\alpha}\mathrm{GTP}} \underbrace{+S_{\mathrm{PM}}k^{\mathrm{PI3K}}_{-\mathrm{G_{i\alpha}GTP}}[\mathrm{PI3K-G_{i\alpha}GTP}]_S - S_{\mathrm{PM}}k^{\mathrm{PI3K}}_{\mathrm{G_{i\alpha}GTP}}[\mathrm{PI3K}]_S[\mathrm{G_{i\alpha}GTP}]_S}_{\text{GTP binding (activation)}} \tag{21}$$

$$S_{\mathrm{PM}}\frac{d}{dt}[\mathrm{G_{i\alpha}GDP}]_S = \underbrace{S_{\mathrm{PM}}k^{\mathrm{G_i}}_{\mathrm{autohydrolysis}}[\mathrm{G_iGTP}]_S}_{\text{Autohydrolysis of }\mathrm{G}_i\mathrm{GTP}} \underbrace{-S_{\mathrm{PM}}k^{\mathrm{G_i}}_{\mathrm{G_{i\beta\gamma}}}[\mathrm{G_{i\alpha}GDP}]_S[\mathrm{G_{i\beta\gamma}}]_S + S_{\mathrm{PM}}k^{\mathrm{G_i}}_{-\mathrm{G_{i\beta\gamma}}}[\mathrm{G_iGDP}]_S}_{\mathrm{G}_{i\beta\gamma}\text{ binding }\mathrm{G}_{i\alpha}\mathrm{GDP}} \underbrace{+S_{\mathrm{PM}}k^{\mathrm{PI3K}}_{-\mathrm{G_ii\alpha GDP}}[\mathrm{PI3K-G_{i\alpha}GDP}]_S - S_{\mathrm{PM}}k^{\mathrm{PI3K}}_{\mathrm{G_{i\alpha}GDP}}[\mathrm{PI3K}]_S[\mathrm{G_{i\alpha}GDP}]_S}_{\text{GDP binding}} \tag{22}$$

$$S_{\mathrm{PM}}\frac{d}{dt}[\mathrm{G_{i\beta\gamma}}]_S = \underbrace{S_{\mathrm{PM}}k^{\mathrm{G_i}}_{\mathrm{autohydrolysis}}[\mathrm{G_iGTP}]_S}_{\text{Autohydrolysis of }\mathrm{G}_i\mathrm{GTP}} \underbrace{-S_{\mathrm{PM}}\left(k^{\mathrm{G_i}}_{\mathrm{G_{i\beta\gamma}}}\frac{S_{\mathrm{PM}}}{V_{\mathrm{cyt}}}\right)[\mathrm{G_{i\alpha}GTP}]_S[\mathrm{G_{i\beta\gamma}}]_S + S_{\mathrm{PM}}k^{\mathrm{G_i}}_{-\mathrm{G_{i\beta\gamma}}}[\mathrm{G_iGTP}]_S}_{\mathrm{G}_{i\beta\gamma}\text{ binding }\mathrm{G}_{i\alpha}\mathrm{GTP}} \underbrace{-S_{\mathrm{PM}}\left(k^{\mathrm{G_i}}_{\mathrm{G_{i\beta\gamma}}}\frac{S_{\mathrm{PM}}}{V_{\mathrm{cyt}}}\right)[\mathrm{G_{i\alpha}GDP}]_S[\mathrm{G_{i\beta\gamma}}]_S + S_{\mathrm{PM}}k^{\mathrm{G_i}}_{-\mathrm{G_{i\beta\gamma}}}[\mathrm{G_iGDP}]_S}_{\mathrm{G}_{i\beta\gamma}\text{ binding }\mathrm{G}_{i\alpha}\mathrm{GDP}} \tag{23}$$

### S4.3.3 PI3K

| Parameter | Value | Units | Reference |
|---|---|---|---|
| $k^{\mathrm{PI3K}}_{\mathrm{hydrolyze}}$ | See Table S1 | 1/s | |
| $k^{\mathrm{PI3K}}_{-\mathrm{G_{i\alpha}GDP}}$ | See Table S1 | 1/s | |
| $k^{\mathrm{PI3K}}_{-\mathrm{G_{i\alpha}GTP}}$ | See Table S1 | 1/s | |
| $k^{\mathrm{PI3K}}_{\mathrm{G_{i\alpha}GDP}}$ | See Table S1 | dm$^2$/mol-s | |
| $k^{\mathrm{PI3K}}_{\mathrm{G_{i\alpha}GTP}}$ | See Table S1 | dm$^2$/mol-s | |
| $k^{\mathrm{PI3K}}_{\mathrm{PIP_3}}$ | See Table S2 | 1/M-s | |
| $k^{\mathrm{PI3K}}_{-\mathrm{PIP_3}}$ | See Table S2 | 1/s | |
| $k^{\mathrm{PI3K}}_{\mathrm{PIP_2,cat}}$ | See Table S2 | 1/s | |
| $N^{\mathrm{PI3K}}$ | 1916 | # | [17] |

Tab. S9: Parameter values associated with PI3K. At time $t = 0$ in the steady-state simulation, $[\mathrm{PI3K}]_S = N^{\mathrm{PI3K}}/S_{\mathrm{PM}}$ and all other states are initialized to 0.

$$S_{\mathrm{PM}}\frac{d}{dt}[\mathrm{PI3K}]_S = \underbrace{S_{\mathrm{PM}}k^{\mathrm{PI3K}}_{-\mathrm{G_{i\alpha}GTP}}[\mathrm{PI3K-G_{i\alpha}GTP}]_S - S_{\mathrm{PM}}k^{\mathrm{PI3K}}_{\mathrm{G_{i\alpha}GTP}}[\mathrm{PI3K}]_S[\mathrm{G_{i\alpha}GTP}]_S}_{\mathrm{G}_{i\alpha}\mathrm{GTP\ binding\ (activation)}} + \underbrace{S_{\mathrm{PM}}k^{\mathrm{PI3K}}_{-\mathrm{G_{i\alpha}GDP}}[\mathrm{PI3K-G_{i\alpha}GDP}]_S - S_{\mathrm{PM}}k^{\mathrm{PI3K}}_{\mathrm{G_{i\alpha}GDP}}[\mathrm{PI3K}]_S[\mathrm{G_{i\alpha}GDP}]_S}_{\mathrm{G}_{i\alpha}\mathrm{GDP\ binding}} \tag{24}$$

$$S_{\mathrm{PM}}\frac{d}{dt}[\mathrm{PI3K-G_{i\alpha}GTP}]_S = \underbrace{-S_{\mathrm{PM}}k^{\mathrm{PI3K}}_{-\mathrm{G_{i\alpha}GTP}}[\mathrm{PI3K-G_{i\alpha}GTP}]_S + S_{\mathrm{PM}}k^{\mathrm{PI3K}}_{\mathrm{G_{i\alpha}GTP}}[\mathrm{PI3K}]_S[\mathrm{G_{i\alpha}GTP}]_S}_{\mathrm{GTP\ binding\ (activation)}} \underbrace{-S_{\mathrm{PM}}k^{\mathrm{PI3K}}_{\mathrm{hydrolyze}}[\mathrm{PI3K-G_{i\alpha}GTP}]_S}_{\mathrm{GTP\ hydrolysis\ (inactivation)}} \underbrace{-S_{\mathrm{PM}}\left(\frac{S_{\mathrm{PM}}}{V_{\mathrm{cyt}}}\right)k^{\mathrm{PI3K}}_{\mathrm{PIP_2}}[\mathrm{PI3K-G_{i\alpha}GTP}]_S[\mathrm{PIP_2}]_S + S_{\mathrm{PM}}k^{\mathrm{PI3K}}_{-\mathrm{PIP_2}}[\mathrm{PI3K-G_{i\alpha}GTP-PIP_2}]_S}_{\mathrm{PI3K\ binding\ PIP_2}} \underbrace{+S_{\mathrm{PM}}k^{\mathrm{PI3K}}_{\mathrm{PIP_2,cat}}[\mathrm{PI3K-G_{i\alpha}GTP-PIP_2}]_S}_{\mathrm{Formation\ of\ PIP_3}} \tag{25}$$

$$S_{\mathrm{PM}}\frac{d}{dt}[\mathrm{PI3K-G_{i\alpha}GDP}]_S = \underbrace{S_{\mathrm{PM}}k^{\mathrm{PI3K}}_{\mathrm{hydrolyze}}[\mathrm{PI3K-G_{i\alpha}GTP}]_S}_{\mathrm{GTP\ hydrolysis\ (inactivation)}} \underbrace{-S_{\mathrm{PM}}k^{\mathrm{PI3K}}_{-\mathrm{G_{i\alpha}GDP}}[\mathrm{PI3K-G_{i\alpha}GDP}]_S + S_{\mathrm{PM}}k^{\mathrm{PI3K}}_{\mathrm{G_{i\alpha}GDP}}[\mathrm{PI3K}]_S[\mathrm{G_{i\alpha}GDP}]_S}_{\mathrm{G}_{i\alpha}\mathrm{GDP\ binding}} \tag{26}$$

$$S_{\mathrm{PM}}\frac{d}{dt}[\mathrm{PI3K}-\mathrm{G_{i\alpha}GTP}-\mathrm{PIP_2}]_S = \underbrace{S_{\mathrm{PM}}\big(\frac{S_{\mathrm{PM}}}{V_{\mathrm{cyt}}}\big)k^{\mathrm{PI3K}}_{\mathrm{PIP_2}}[\mathrm{PI3K}-\mathrm{G_{i\alpha}GTP}]_S[\mathrm{PIP_2}]_S - S_{\mathrm{PM}}k^{\mathrm{PI3K}}_{-\mathrm{PIP_2}}[\mathrm{PI3K}-\mathrm{G_{i\alpha}GTP}-\mathrm{PIP_2}]_S}_{\text{PI3K binding PIP}_2} \underbrace{-S_{\mathrm{PM}}k^{\mathrm{PI3K}}_{\mathrm{PIP_2,cat}}[\mathrm{PI3K}-\mathrm{G_{i\alpha}GTP}-\mathrm{PIP_2}]_S}_{\text{Formation of PIP}_3} \quad (27)$$

## S4.4 Proteins in $P2Y_1$ pathway

## S4.5 $P2Y_1$

We continue to use the model from Purvis et al. with a Quasi-Steady State assumption on certain states that rapidly switch between "active" and "inactive" states. We also introduce two new states, ADP-p$P2Y_1$ and ADP-p$P2Y_1$-$G_q$GDP, that represent desensitization via PKC phosphorylation.

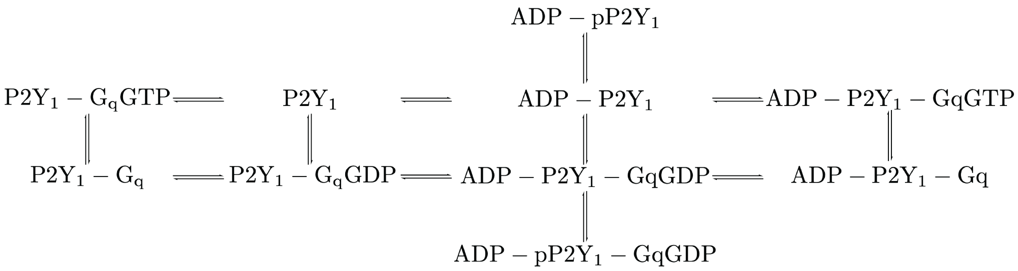


Fig. S2: $P2Y_1$ reaction diagram

| Parameter | Value | Units | Reference |
|---|---|---|---|
| $K_{\mathrm{act}}^{\mathrm{P2Y_1}}$ | See Table S1 | n.d. | |
| $\alpha^{P2Y_1}$ | See Table S1 | n.d. | |
| $\beta^{P2Y_1}$ | See Table S1 | n.d. | |
| $\delta^{P2Y_1}$ | See Table S1 | n.d. | |
| $\gamma^{P2Y_1}$ | See Table S1 | n.d. | |
| $k_{\mathrm{G_qGDP}}^{\mathrm{P2Y_1}}$ | See Table S1 | $\mathrm{dm^2/mol\text{-}s}$ | |
| $k_{\mathrm{-G_qGDP}}^{\mathrm{P2Y_1}}$ | See Table S1 | 1/s | |
| $k_{\mathrm{G_qGTP}}^{\mathrm{P2Y_1}}$ | See Table S1 | $\mathrm{dm^2/mol\text{-}s}$ | |
| $k_{\mathrm{-G_qGTP}}^{\mathrm{P2Y_1}}$ | See Table S1 | 1/s | |
| $k_{\mathrm{ADP}}^{\mathrm{P2Y_1}}$ | See Table S1 | 1/M-s | |
| $k_{\mathrm{-ADP}}^{\mathrm{P2Y_1}}$ | See Table S1 | 1/M-s | |
| $k_{\mathrm{-GDP}}^{\mathrm{P2Y_1}}$ | See Table S1 | 1/s | |
| $k_{\mathrm{GDP}}^{\mathrm{P2Y_1}}$ | See Table S1 | 1/M-s | |
| $k_{\mathrm{-GTP}}^{\mathrm{P2Y_1}}$ | See Table S1 | 1/s | |
| $k_{\mathrm{GTP}}^{\mathrm{P2Y_1}}$ | See Table S1 | 1/M-s | |
| $k_{\mathrm{phos}}^{\mathrm{P2Y_1}}$ | See Table S1 | 1/M-s | |
| $k_{\mathrm{dephos}}^{\mathrm{P2Y_1}}$ | See Table S1 | 1/s | |
| [GDP] | $1.3362 \cdot 10^{-5}$ | M | [12] |
| [GTP] | $7.117 \cdot 10^{-4}$ | M | [12] |
| $N^{\mathrm{P2Y_1}}$ | 983 | # | [17] |

Tab. S10: Parameter values associated with $P2Y_{12}$. At time At time $t = 0$ s in the steady-state simulation, $[P2Y_1]_S = N^{\mathrm{P2Y_1}}/S_{\mathrm{PM}}$ and all other states are initialized to 0.

$$
\begin{aligned}
S_{\mathrm{PM}}\frac{d}{dt}[\mathrm{P2Y_1}]_S = &\underbrace{-S_{\mathrm{PM}}\left(k_{\mathrm{ADP}}^{\mathrm{P2Y_1}}\frac{\alpha^{\mathrm{P2Y_1}}K_{\mathrm{act}}^{\mathrm{P2Y_1}}+1}{K_{\mathrm{act}}^{\mathrm{P2Y_1}}+1}\right)[\mathrm{ADP}][\mathrm{P2Y_1}]_S + S_{\mathrm{PM}}k_{-\mathrm{ADP}}^{\mathrm{P2Y_1}}[\mathrm{ADP}-\mathrm{P2Y_1}]_S}_{\text{ADP Binding}} \\
&\underbrace{\begin{aligned}&-S_{\mathrm{PM}}\left(k_{\mathrm{G_qGDP}}^{\mathrm{P2Y_1}}\frac{\beta^{\mathrm{P2Y_1}}K_{\mathrm{act}}^{\mathrm{P2Y_1}}}{K_{\mathrm{act}}^{\mathrm{P2Y_1}}+1}\right)[\mathrm{G_qGDP}]_S[\mathrm{P2Y_1}]_S \\ &\quad+S_{\mathrm{PM}}\left(k_{-\mathrm{G_qGDP}}^{\mathrm{P2Y_1}}\frac{\beta^{\mathrm{P2Y_1}}K_{\mathrm{act}}^{\mathrm{P2Y_1}}}{\beta^{\mathrm{P2Y_1}}K_{\mathrm{act}}^{\mathrm{P2Y_1}}+1}\right)[\mathrm{P2Y_1}-\mathrm{G_qGDP}]_S\end{aligned}}_{\mathrm{G}_q\text{GDP Binding}} \\
&\underbrace{\begin{aligned}&-S_{\mathrm{PM}}\left(k_{\mathrm{G_qGTP}}^{\mathrm{P2Y_1}}\frac{K_{\mathrm{act}}^{\mathrm{P2Y_1}}}{K_{\mathrm{act}}^{\mathrm{P2Y_1}}+1}\right)[\mathrm{G_qGTP}]_S[\mathrm{P2Y_1}]_S \\ &\quad+S_{\mathrm{PM}}k_{-\mathrm{G_qGTP}}^{\mathrm{P2Y_1}}[\mathrm{P2Y_1}-\mathrm{G_qGTP}]_S\end{aligned}}_{\mathrm{G}_q\text{GTP Binding}}
\end{aligned} \tag{28}
$$

$$
\begin{aligned}
S_{\mathrm{PM}}\frac{d}{dt}[\mathrm{ADP}-\mathrm{P2Y_1}]_S = &\underbrace{S_{\mathrm{PM}}\left(k_{\mathrm{ADP}}^{\mathrm{P2Y_1}}\frac{\alpha^{\mathrm{P2Y_1}}K_{\mathrm{act}}^{\mathrm{P2Y_1}}+1}{K_{\mathrm{act}}^{\mathrm{P2Y_1}}+1}\right)[\mathrm{ADP}][\mathrm{P2Y_1}]_S - S_{\mathrm{PM}}k_{-\mathrm{ADP}}^{\mathrm{P2Y_1}}[\mathrm{ADP}-\mathrm{P2Y_1}]_S}_{\text{ADP Binding}} \\
&\underbrace{\begin{aligned}&-S_{\mathrm{PM}}\left(k_{\mathrm{G_qGDP}}^{\mathrm{P2Y_1}}\frac{\alpha^{\mathrm{P2Y_1}}\beta^{\mathrm{P2Y_1}}K_{\mathrm{act}}^{\mathrm{P2Y_1}}}{\alpha^{\mathrm{P2Y_1}}K_{\mathrm{act}}^{\mathrm{P2Y_1}}+1}\right)[\mathrm{G_qGDP}]_S[\mathrm{ADP}-\mathrm{P2Y_1}]_S \\ &\quad+S_{\mathrm{PM}}\left(\frac{k_{-\mathrm{G_qGDP}}^{\mathrm{P2Y_1}}}{\gamma^{\mathrm{P2Y_1}}}\frac{\alpha^{\mathrm{P2Y_1}}\beta^{\mathrm{P2Y_1}}K_{\mathrm{act}}^{\mathrm{P2Y_1}}}{\alpha^{\mathrm{P2Y_1}}\beta^{\mathrm{P2Y_1}}\delta^{\mathrm{P2Y_1}}K_{\mathrm{act}}^{\mathrm{P2Y_1}}+1}\right)[\mathrm{ADP}-\mathrm{P2Y_1}-\mathrm{G_qGDP}]_S\end{aligned}}_{\mathrm{G}_q\text{GDP Binding}} \\
&\underbrace{\begin{aligned}&-S_{\mathrm{PM}}\left(k_{\mathrm{G_qGTP}}^{\mathrm{P2Y_1}}\frac{\alpha^{\mathrm{P2Y_1}}K_{\mathrm{act}}^{\mathrm{P2Y_1}}}{\alpha^{\mathrm{P2Y_1}}K_{\mathrm{act}}^{\mathrm{P2Y_1}}+1}\right)[\mathrm{G_qGTP}]_S[\mathrm{ADP}-\mathrm{P2Y_1}]_S \\ &\quad+S_{\mathrm{PM}}k_{-\mathrm{G_qGTP}}^{\mathrm{P2Y_1}}[\mathrm{ADP}-\mathrm{P2Y_1}-\mathrm{G_qGTP}]_S\end{aligned}}_{\mathrm{G}_q\text{GTP Binding}} \\
&\underbrace{\begin{aligned}&-S_{\mathrm{PM}}k_{\mathrm{phos}}^{\mathrm{P2Y_1}}[\mathrm{ADP}-\mathrm{P2Y_1}]_S[\mathrm{PKCa}-\mathrm{Ca}-\mathrm{DAG}] \\ &\quad+S_{\mathrm{PM}}k_{\mathrm{dephos}}^{\mathrm{P2Y_1}}[\mathrm{ADP}-\mathrm{pP2Y_1}]_S\end{aligned}}_{\text{Inactivation by PKC}}
\end{aligned} \tag{29}
$$

$$
\begin{aligned}
S_{\mathrm{PM}}\frac{d}{dt}[\mathrm{P2Y_1-G_qGDP}]_S = & \underbrace{-S_{\mathrm{PM}}\left(k_{\mathrm{ADP}}^{\mathrm{P2Y_1}}\frac{\alpha^{\mathrm{P2Y_1}}\beta^{\mathrm{P2Y_1}}K_{\mathrm{act}}^{\mathrm{P2Y_1}}+1}{\beta^{\mathrm{P2Y_1}}K_{\mathrm{act}}^{\mathrm{P2Y_1}}+1}\right)[\mathrm{ADP}][\mathrm{P2Y_1-G_qGDP}]_S + S_{\mathrm{PM}}\left(\frac{k_{\mathrm{-ADP}}^{\mathrm{P2Y_1}}}{\gamma^{\mathrm{P2Y_1}}}\frac{\alpha^{\mathrm{P2Y_1}}\beta^{\mathrm{P2Y_1}}K_{\mathrm{act}}^{\mathrm{P2Y_1}}+1}{\alpha^{\mathrm{P2Y_1}}\beta^{\mathrm{P2Y_1}}\delta^{\mathrm{P2Y_1}}K_{\mathrm{act}}^{\mathrm{P2Y_1}}+1}\right)[\mathrm{ADP-P2Y_1-G_qGDP}]_S}_{\text{ADP Binding}} \\
& \underbrace{+S_{\mathrm{PM}}\left(k_{\mathrm{G_qGDP}}^{\mathrm{P2Y_1}}\frac{\beta^{\mathrm{P2Y_1}}K_{\mathrm{act}}^{\mathrm{P2Y_1}}}{K_{\mathrm{act}}^{\mathrm{P2Y_1}}+1}\right)[\mathrm{G_qGDP}]_S[\mathrm{P2Y_1}]_S - S_{\mathrm{PM}}\left(k_{\mathrm{-G_qGDP}}^{\mathrm{P2Y_1}}\frac{\beta^{\mathrm{P2Y_1}}K_{\mathrm{act}}^{\mathrm{P2Y_1}}}{\beta^{\mathrm{P2Y_1}}K_{\mathrm{act}}^{\mathrm{P2Y_1}}+1}\right)[\mathrm{P2Y_1-G_qGDP}]_S}_{\mathrm{G}_q\text{GDP Binding}} \\
& \underbrace{+S_{\mathrm{PM}}k_{\mathrm{GDP}}^{\mathrm{P2Y_1}}[\mathrm{GDP}][\mathrm{P2Y_1-G_q}]_S - S_{\mathrm{PM}}\left(k_{\mathrm{-GDP}}^{\mathrm{P2Y_1}}\frac{\beta^{\mathrm{P2Y_1}}K_{\mathrm{act}}^{\mathrm{P2Y_1}}}{\beta^{\mathrm{P2Y_1}}K_{\mathrm{act}}^{\mathrm{P2Y_1}}+1}\right)[\mathrm{P2Y_1-G_qGDP}]_S}_{\text{GDP Binding}}
\end{aligned}
\tag{30}
$$

$$
\begin{aligned}
S_{\mathrm{PM}}\frac{d}{dt}[\mathrm{ADP-P2Y_1-G_qGDP}]_S = & \underbrace{S_{\mathrm{PM}}\left(k_{\mathrm{ADP}}^{\mathrm{P2Y_1}}\frac{\alpha^{\mathrm{P2Y_1}}\beta^{\mathrm{P2Y_1}}K_{\mathrm{act}}^{\mathrm{P2Y_1}}+1}{\beta^{\mathrm{P2Y_1}}K_{\mathrm{act}}^{\mathrm{P2Y_1}}+1}\right)[\mathrm{ADP}][\mathrm{P2Y_1-G_qGDP}]_S - S_{\mathrm{PM}}\left(\frac{k_{\mathrm{-ADP}}^{\mathrm{P2Y_1}}}{\gamma^{\mathrm{P2Y_1}}}\frac{\alpha^{\mathrm{P2Y_1}}\beta^{\mathrm{P2Y_1}}K_{\mathrm{act}}^{\mathrm{P2Y_1}}+1}{\alpha^{\mathrm{P2Y_1}}\beta^{\mathrm{P2Y_1}}\delta^{\mathrm{P2Y_1}}K_{\mathrm{act}}^{\mathrm{P2Y_1}}+1}\right)[\mathrm{ADP-P2Y_1-G_qGDP}]_S}_{\text{ADP Binding}} \\
& \underbrace{+S_{\mathrm{PM}}\left(k_{\mathrm{G_qGDP}}^{\mathrm{P2Y_1}}\frac{\alpha^{\mathrm{P2Y_1}}\beta^{\mathrm{P2Y_1}}K_{\mathrm{act}}^{\mathrm{P2Y_1}}}{\alpha^{\mathrm{P2Y_1}}K_{\mathrm{act}}^{\mathrm{P2Y_1}}+1}\right)[\mathrm{G_qGDP}]_S[\mathrm{ADP-P2Y_1}]_S - S_{\mathrm{PM}}\left(\frac{k_{\mathrm{-G_qGDP}}^{\mathrm{P2Y_1}}}{\gamma^{\mathrm{P2Y_1}}}\frac{\alpha^{\mathrm{P2Y_1}}\beta^{\mathrm{P2Y_1}}K_{\mathrm{act}}^{\mathrm{P2Y_1}}}{\alpha^{\mathrm{P2Y_1}}\beta^{\mathrm{P2Y_1}}\delta^{\mathrm{P2Y_1}}K_{\mathrm{act}}^{\mathrm{P2Y_1}}+1}\right)[\mathrm{ADP-P2Y_1-G_qGDP}]_S}_{\mathrm{G}_q\text{GDP Binding}} \\
& \underbrace{+S_{\mathrm{PM}}k_{\mathrm{GDP}}^{\mathrm{P2Y_1}}[\mathrm{GDP}][\mathrm{ADP-P2Y_1-G_q}]_S - S_{\mathrm{PM}}\left(k_{\mathrm{-GDP}}^{\mathrm{P2Y_1}}\frac{\alpha^{\mathrm{P2Y_1}}\beta^{\mathrm{P2Y_1}}\delta^{\mathrm{P2Y_1}}K_{\mathrm{act}}^{\mathrm{P2Y_1}}}{\alpha^{\mathrm{P2Y_1}}\beta^{\mathrm{P2Y_1}}\delta^{\mathrm{P2Y_1}}K_{\mathrm{act}}^{\mathrm{P2Y_1}}+1}\right)[\mathrm{ADP-P2Y_1-G_qGDP}]_S}_{\text{GDP Binding}} \\
& \underbrace{-S_{\mathrm{PM}}k_{\mathrm{phos}}^{\mathrm{P2Y_1}}[\mathrm{ADP-P2Y_1-G_qGDP}]_S[\mathrm{PKCa-Ca-DAG}] + S_{\mathrm{PM}}k_{\mathrm{dephos}}^{\mathrm{P2Y_1}}[\mathrm{ADP-pP2Y_1-G_qGDP}]_S}_{\text{Inactivation by PKC}}
\end{aligned}
\tag{31}
$$

$$S_{\mathrm{PM}}\frac{d}{dt}[\mathrm{P2Y_1-G_qGTP}]_S = \underbrace{S_{\mathrm{PM}}k_{\mathrm{GTP}}^{\mathrm{P2Y_1}}[\mathrm{P2Y_1-G_q}]_S[\mathrm{GTP}] - S_{\mathrm{PM}}k_{\mathrm{-GTP}}^{\mathrm{P2Y_1}}[\mathrm{P2Y_1-G_qGTP}]_S}_{\text{GTP Binding}} \\ \underbrace{+ S_{\mathrm{PM}}\left(k_{\mathrm{G_qGTP}}^{\mathrm{P2Y_1}}\frac{K_{\mathrm{act}}^{\mathrm{P2Y_1}}}{K_{\mathrm{act}}^{\mathrm{P2Y_1}}+1}\right)[\mathrm{G_qGTP}]_S[\mathrm{P2Y_1}]_S - S_{\mathrm{PM}}k_{\mathrm{-G_qGTP}}^{\mathrm{P2Y_1}}[\mathrm{P2Y_1-G_qGTP}]_S}_{\mathrm{G}_q\text{GTP binding}} \tag{32}$$

$$S_{\mathrm{PM}}\frac{d}{dt}[\mathrm{ADP-P2Y_1-G_qGTP}]_S = \underbrace{S_{\mathrm{PM}}k_{\mathrm{GTP}}^{\mathrm{P2Y_1}}[\mathrm{ADP-P2Y_1-G_q}]_S[\mathrm{GTP}] - S_{\mathrm{PM}}k_{\mathrm{-GTP}}^{\mathrm{P2Y_1}}[\mathrm{ADP-P2Y_1-G_qGTP}]_S}_{\text{GTP Binding}} \\ \underbrace{+ S_{\mathrm{PM}}\left(k_{\mathrm{G_qGTP}}^{\mathrm{P2Y_1}}\frac{\alpha^{\mathrm{P2Y_1}}K_{\mathrm{act}}^{\mathrm{P2Y_1}}}{\alpha^{\mathrm{P2Y_1}}K_{\mathrm{act}}^{\mathrm{P2Y_1}}+1}\right)[\mathrm{G_qGTP}]_S[\mathrm{ADP-P2Y_1}]_S - S_{\mathrm{PM}}k_{\mathrm{-G_qGTP}}^{\mathrm{P2Y_1}}[\mathrm{ADP-P2Y_1-G_qGTP}]_S}_{\mathrm{G}_q\text{GTP binding}} \tag{33}$$

$$S_{\mathrm{PM}}\frac{d}{dt}[\mathrm{P2Y_1-G_q}]_S = \underbrace{- S_{\mathrm{PM}}k_{\mathrm{GTP}}^{\mathrm{P2Y_1}}[\mathrm{P2Y_1-G_q}]_S[\mathrm{GTP}] + S_{\mathrm{PM}}k_{\mathrm{-GTP}}^{\mathrm{P2Y_1}}[\mathrm{P2Y_1-G_qGTP}]_S}_{\text{GTP Binding}} \\ \underbrace{+ S_{\mathrm{PM}}\left(k_{\mathrm{-GDP}}^{\mathrm{P2Y_1}}\frac{\beta^{\mathrm{P2Y_1}}K_{\mathrm{act}}^{\mathrm{P2Y_1}}}{\beta^{\mathrm{P2Y_1}}K_{\mathrm{act}}^{\mathrm{P2Y_1}}+1}\right)[\mathrm{P2Y_1-G_qGDP}]_S - S_{\mathrm{PM}}k_{\mathrm{GDP}}^{\mathrm{P2Y_1}}[\mathrm{P2Y_1-G_q}]_S[\mathrm{GDP}]}_{\text{GDP binding}} \tag{34}$$

$$S_{\mathrm{PM}}\frac{d}{dt}[\mathrm{ADP-P2Y_1-G_q}]_S = \underbrace{- S_{\mathrm{PM}}k_{\mathrm{GTP}}^{\mathrm{P2Y_1}}[\mathrm{ADP-P2Y_1-G_q}]_S[\mathrm{GTP}] + S_{\mathrm{PM}}k_{\mathrm{-GTP}}^{\mathrm{P2Y_1}}[\mathrm{ADP-P2Y_1-G_qGTP}]_S}_{\text{GTP Binding}} \\ \underbrace{- S_{\mathrm{PM}}k_{\mathrm{GDP}}^{\mathrm{P2Y_1}}[\mathrm{ADP-P2Y_1-G_q}]_S[\mathrm{GDP}] + S_{\mathrm{PM}}\left(k_{\mathrm{-GDP}}^{\mathrm{P2Y_1}}\frac{\alpha^{\mathrm{P2Y_1}}\beta^{\mathrm{P2Y_1}}\delta^{\mathrm{P2Y_1}}K_{\mathrm{act}}^{\mathrm{P2Y_1}}}{\alpha^{\mathrm{P2Y_1}}\beta^{\mathrm{P2Y_1}}\delta^{\mathrm{P2Y_1}}K_{\mathrm{act}}^{\mathrm{P2Y_1}}+1}\right)[\mathrm{ADP-P2Y_1-G_qGDP}]_S}_{\text{GDP binding}} \tag{35}$$

$$S_{\mathrm{PM}}\frac{d}{dt}[\mathrm{ADP-pP2Y_1}]_S = \underbrace{S_{\mathrm{PM}}k_{\mathrm{phos}}^{\mathrm{P2Y_1}}[\mathrm{ADP-P2Y_1}]_S[\mathrm{PKCa-Ca-DAG}] - S_{\mathrm{PM}}k_{\mathrm{dephos}}^{\mathrm{P2Y_1}}[\mathrm{ADP-pP2Y_1}]_S}_{\text{Inactivation by PKC}} \tag{36}$$

$$S_{\mathrm{PM}}\frac{d}{dt}[\mathrm{ADP-pP2Y_1-G_qGDP}]_S = \underbrace{S_{\mathrm{PM}}k_{\mathrm{phos}}^{\mathrm{P2Y_1}}[\mathrm{ADP-P2Y_1-GqGDP}]_S[\mathrm{PKCa-Ca-DAG}] - S_{\mathrm{PM}}k_{\mathrm{dephos}}^{\mathrm{P2Y_1}}[\mathrm{ADP-pP2Y_1-G_qGDP}]_S}_{\text{Inactivation by PKC}} \tag{37}$$

### S4.5.1 $G_q$

| Parameter | Value | Units | Reference |
|---|---|---|---|
| $k_{GTP}$ | See Table S1 | 1/s | |
| $k^{G_q}_{G_{q\beta\gamma}}$ | 103584 | 1/M-s | [7] |
| $k^{G_q}_{-G_{q\beta\gamma}}$ | 7.7832 | 1/s | [7] |
| $N^{G_q}$ | 18583 | # | [17] |

Tab. S11: Parameter values associated with $G_q$. At time $t = 0$ s in the steady-state simulation, $[G_q GDP]_S = N^{G_q}/S_{PM}$ and all other states are initialized to 0.

$$\begin{aligned} S_{PM}\frac{d}{dt}[G_{q\alpha}GTP]_S = &\underbrace{-S_{PM}\left(k^{G_q}_{G_{\beta\gamma}}\frac{S_{PM}}{V_{cyt}}\right)[G_{q\alpha}GTP]_S[G_{q\beta\gamma}]_S + S_{PM}k^{G_q}_{-G_{q\beta\gamma}}[G_qGTP]_S}_{G_{q\beta\gamma}\text{ binding }G_{q\alpha}GTP} \\ &\underbrace{+S_{PM}k^{PLC}_{-G_{q\alpha}GTP}[PLC-G_{q\alpha}GTP]_S - S_{PM}k^{PLC}_{G_{q\alpha}GTP}[PLC]_S[G_{q\alpha}GTP]_S}_{\text{PLC binding }G_{q\alpha}GTP} \end{aligned} \tag{38}$$

$$\begin{aligned} S_{PM}\frac{d}{dt}[G_{q\alpha}GDP]_S = &\underbrace{S_{PM}k^{G_q}_{autohydrolysis}[G_qGTP]_S}_{\text{Autohydrolysis of }G_q\text{ GTP}} \\ &\underbrace{-S_{PM}\left(k^{G_q}_{G_{\beta\gamma}}\frac{S_{PM}}{V_{cyt}}\right)[G_{q\alpha}GDP]_S[G_{q\beta\gamma}]_S + V_{cyt}k^{G_q}_{-G_{\beta\gamma}}[G_qGDP]_S}_{G_{q\beta\gamma}\text{ binding }G_{q\alpha}GDP} \\ &\underbrace{+S_{PM}k^{PLC}_{-G_{q\alpha}GDP}[PLC-G_{q\alpha}GDP]_S - S_{PM}k^{PLC}_{G_{q\alpha}GDP}[PLC]_S[G_{q\alpha}GDP]_S}_{\text{PLC binding }G_{q\alpha}GDP} \end{aligned} \tag{39}$$

$$\begin{aligned} S_{PM}\frac{d}{dt}[G_{q\beta\gamma}]_S = &\underbrace{S_{PM}k^{G_q}_{autohydrolysis}[G_qGTP]_S}_{\text{Autohydrolysis of }G_iGTP} \\ &\underbrace{-S_{PM}\left(k^{G_q}_{G_{q\beta\gamma}}\frac{S_{PM}}{V_{cyt}}\right)[G_{q\alpha}GTP]_S[G_{q\beta\gamma}]_S + S_{PM}k^{G_q}_{-G_{q\beta\gamma}}[G_qGTP]_S}_{G_{q\beta\gamma}\text{ binding }G_{q\alpha}GTP} \\ &\underbrace{-S_{PM}\left(k^{G_q}_{G_{q\beta\gamma}}\frac{S_{PM}}{V_{cyt}}\right)[G_{q\alpha}GDP]_S[G_{q\beta\gamma}]_S + S_{PM}k^{G_q}_{-G_{q\beta\gamma}}[G_qGDP]_S}_{G_{q\beta\gamma}\text{ binding }G_{q\alpha}GDP} \end{aligned} \tag{40}$$

$$S_{\mathrm{PM}}\frac{d}{dt}[\mathrm{G_qGDP}]_S = \underbrace{-S_{\mathrm{PM}}\left(k^{\mathrm{P2Y_1}}_{\mathrm{G_qGDP}}\frac{\beta^{\mathrm{P2Y_1}}K^{\mathrm{P2Y_1}}_{\mathrm{act}}}{K^{\mathrm{P2Y_1}}_{\mathrm{act}}+1}\right)[\mathrm{G_qGDP}]_S[\mathrm{P2Y_1}]_S + S_{\mathrm{PM}}\left(k^{\mathrm{P2Y_1}}_{-\mathrm{G_qGDP}}\frac{\beta^{\mathrm{P2Y_1}}K^{\mathrm{P2Y_1}}_{\mathrm{act}}}{\beta^{\mathrm{P2Y_1}}K^{\mathrm{P2Y_1}}_{\mathrm{act}}+1}\right)[\mathrm{P2Y_1-G_qGDP}]_S}_{\mathrm{G}_q\mathrm{GDP\ Binding\ P2Y_1}}$$
$$\underbrace{-S_{\mathrm{PM}}\left(k^{\mathrm{P2Y_1}}_{\mathrm{G_qGDP}}\frac{\alpha^{\mathrm{P2Y_1}}\beta^{\mathrm{P2Y_1}}K^{\mathrm{P2Y_1}}_{\mathrm{act}}}{\alpha^{\mathrm{P2Y_1}}K^{\mathrm{P2Y_1}}_{\mathrm{act}}+1}\right)[\mathrm{G_qGDP}]_S[\mathrm{ADP-P2Y_1}]_S + S_{\mathrm{PM}}\left(\frac{k^{\mathrm{P2Y_1}}_{-\mathrm{G_qGDP}}}{\gamma^{\mathrm{P2Y_1}}}\frac{\alpha^{\mathrm{P2Y_1}}\beta^{\mathrm{P2Y_1}}K^{\mathrm{P2Y_1}}_{\mathrm{act}}}{\alpha^{\mathrm{P2Y_1}}\beta^{\mathrm{P2Y_1}}\delta^{\mathrm{P2Y_1}}K^{\mathrm{P2Y_1}}_{\mathrm{act}}+1}\right)[\mathrm{ADP-P2Y_1-G_qGDP}]_S}_{\mathrm{G}_q\mathrm{GDP\ Binding\ ADP-P2Y_1}}$$
$$\underbrace{+S_{\mathrm{PM}}\left(k^{\mathrm{G_q}}_{\mathrm{G_{q\beta\gamma}}}\frac{S_{\mathrm{PM}}}{V_{\mathrm{cyt}}}\right)[\mathrm{G_{q\alpha}GDP}]_S[\mathrm{G_{q\beta\gamma}}]_S - S_{\mathrm{PM}}k^{\mathrm{G_q}}_{-\mathrm{G_{q\beta\gamma}}}[\mathrm{G_qGDP}]_S}_{\mathrm{G}_{q\beta\gamma}\mathrm{\ binding\ G}_{q\alpha}\mathrm{GDP}} \tag{41}$$

$$S_{\mathrm{PM}}\frac{d}{dt}[\mathrm{G_qGTP}]_S = \underbrace{-S_{\mathrm{PM}}\left(k^{\mathrm{P2Y_1}}_{\mathrm{G_qGTP}}\frac{K^{\mathrm{P2Y_1}}_{\mathrm{act}}}{K^{\mathrm{P2Y_1}}_{\mathrm{act}}+1}\right)[\mathrm{G_qGTP}]_S[\mathrm{P2Y_1}]_S + S_{\mathrm{PM}}k^{\mathrm{P2Y_1}}_{-\mathrm{G_qGTP}}[\mathrm{P2Y_1-G_qGTP}]_S}_{\mathrm{G}_q\mathrm{GTP\ Binding}}$$
$$\underbrace{-S_{\mathrm{PM}}\left(k^{\mathrm{P2Y_1}}_{\mathrm{G_qGTP}}\frac{\alpha^{\mathrm{P2Y_1}}K^{\mathrm{P2Y_1}}_{\mathrm{act}}}{\alpha^{\mathrm{P2Y_1}}K^{\mathrm{P2Y_1}}_{\mathrm{act}}+1}\right)[\mathrm{G_qGTP}]_S[\mathrm{ADP-P2Y_1}]_S + S_{\mathrm{PM}}k^{\mathrm{P2Y_1}}_{-\mathrm{G_qGTP}}[\mathrm{ADP-P2Y_1-G_qGTP}]_S}_{\mathrm{G}_q\mathrm{GTP\ Binding}}$$
$$\underbrace{-S_{\mathrm{PM}}k^{\mathrm{G_q}}_{\mathrm{autohydrolysis}}[\mathrm{G_qGTP}]_S}_{\mathrm{Autohydrolysis\ of\ }G_{\mathrm{q}}\mathrm{\ GTP}}$$
$$\underbrace{+S_{\mathrm{PM}}\left(k^{\mathrm{G_q}}_{\mathrm{G_{q\beta\gamma}}}\frac{S_{\mathrm{PM}}}{V_{\mathrm{cyt}}}\right)[\mathrm{G_{q\alpha}GTP}]_S[\mathrm{G_{q\beta\gamma}}]_S - S_{\mathrm{PM}}k^{\mathrm{G_q}}_{-\mathrm{G_{q\beta\gamma}}}[\mathrm{G_qGTP}]_S}_{\mathrm{G}_{q\beta\gamma}\mathrm{\ binding\ G}_{q\alpha}\mathrm{GTP}} \tag{42}$$

### S4.5.2 PLC

| Parameter | Value | Units | Reference |
|---|---|---|---|
| $k^{\mathrm{PLC}}_{\mathrm{hydrolyze}}$ | See Table S1 | 1/s | |
| $k^{\mathrm{PLC}}_{-\mathrm{G_{q\alpha}GDP}}$ | See Table S1 | 1/s | |
| $k^{\mathrm{PLC}}_{-\mathrm{G_{q\alpha}GTP}}$ | See Table S1 | 1/s | |
| $k^{\mathrm{PLC}}_{\mathrm{G_{q\alpha}GDP}}$ | See Table S1 | dm$^2$/mol-s | |
| $k^{\mathrm{PLC}}_{\mathrm{G_{q\alpha}GTP}}$ | See Table S1 | dm$^2$/mol-s | |
| $k^{\mathrm{PLC}}_{\mathrm{PI}}$ | $1 \cdot 10^8$ | 1/M-s | [4] |
| $k^{\mathrm{PLC}}_{-\mathrm{PI}}$ | $7.05 \cdot 10^4$ | 1/s | [4] |
| $k^{\mathrm{PLC}}_{\mathrm{PI,cat}}$ | 1.43 | 1/s | [4] |
| $k^{\mathrm{PLC}}_{\mathrm{PIP}}$ | $1 \cdot 10^8$ | 1/M-s | [4] |
| $k^{\mathrm{PLC}}_{-\mathrm{PIP}}$ | $1.9 \cdot 10^7$ | 1/s | [4] |
| $k^{\mathrm{PLC}}_{\mathrm{PIP,cat}}$ | 0.35 | 1/s | [4] |
| $k^{\mathrm{PLC}}_{\mathrm{PIP_2}}$ | $1 \cdot 10^8$ | 1/M-s | [4] |
| $k^{\mathrm{PLC}}_{-\mathrm{PIP_2}}$ | $5 \cdot 10^4$ | 1/s | [4] |
| $k^{\mathrm{PLC}}_{\mathrm{PIP_2,cat}}$ | 9.8505 | 1/s | [4] |
| $N^{\mathrm{PLC}}$ | 1579 | # | [17] |

Tab. S12: Parameter values associated with PLC. At time $t = 0$ s in the steady-state simulation, $[\mathrm{PLC}]_S = N^{\mathrm{PLC}}/S_{\mathrm{PM}}$ and all other states are initialized to 0.

$$S_{\mathrm{PM}}\frac{d}{dt}[\mathrm{PLC}]_S = \underbrace{S_{\mathrm{PM}}k^{\mathrm{PLC}}_{-\mathrm{G_{q\alpha}GTP}}[\mathrm{PLC}-\mathrm{G_{q\alpha}GTP}]_S - S_{\mathrm{PM}}k^{\mathrm{PLC}}_{\mathrm{G_{q\alpha}GTP}}[\mathrm{PLC}]_S[\mathrm{G_{q\alpha}GTP}]_S}_{G_{q\alpha}\mathrm{GTP\ binding}} \\ \underbrace{+S_{\mathrm{PM}}k^{\mathrm{PLC}}_{-\mathrm{G_{q\alpha}GDP}}[\mathrm{PLC}-\mathrm{G_{q\alpha}GDP}]_S - S_{\mathrm{PM}}k^{\mathrm{PLC}}_{\mathrm{G_{q\alpha}GDP}}[\mathrm{PLC}]_S[\mathrm{G_{q\alpha}GDP}]_S}_{G_{q\alpha}\mathrm{GDP\ binding}} \quad (43)$$

$$S_{\mathrm{PM}}\frac{d}{dt}[\mathrm{PLC}-\mathrm{G_{q\alpha}GTP}]_S = \underbrace{-S_{\mathrm{PM}}k^{\mathrm{PLC}}_{-\mathrm{G_{q\alpha}GTP}}[\mathrm{PLC}-\mathrm{G_{q\alpha}GTP}]_S + S_{\mathrm{PM}}k^{\mathrm{PLC}}_{\mathrm{G_{q\alpha}GTP}}[\mathrm{PLC}]_S[\mathrm{G_{q\alpha}GTP}]_S}_{G_{q\alpha}\mathrm{GTP\ binding}}$$
$$\underbrace{-S_{\mathrm{PM}}k^{\mathrm{PLC}}_{\mathrm{hydrolyze}}[\mathrm{PLC}-\mathrm{G_{q\alpha}GTP}]_S}_{\mathrm{GTP\ hydrolysis\ (inactivation)}}$$
$$\underbrace{-S_{\mathrm{PM}}\Big(\frac{S_{\mathrm{PM}}}{V_{\mathrm{cyt}}}\Big)k^{\mathrm{PLC}}_{\mathrm{PI}}[\mathrm{PLC}-\mathrm{G_{q\alpha}GTP}]_S[\mathrm{PI}]_S + S_{\mathrm{PM}}k^{\mathrm{PLC}}_{-\mathrm{PI}}[\mathrm{PLC}-\mathrm{G_{q\alpha}GTP}-\mathrm{PI}]_S}_{\mathrm{PLC\ binding\ PI}}$$
$$\underbrace{+S_{\mathrm{PM}}k^{\mathrm{PLC}}_{\mathrm{PI,cat}}[\mathrm{PLC}-\mathrm{G_{q\alpha}GTP}-\mathrm{PI}]_S}_{\mathrm{Formation\ of\ I_1P}}$$
$$\underbrace{-S_{\mathrm{PM}}\Big(\frac{S_{\mathrm{PM}}}{V_{\mathrm{cyt}}}\Big)k^{\mathrm{PLC}}_{\mathrm{PIP}}[\mathrm{PLC}-\mathrm{G_{q\alpha}GTP}]_S[\mathrm{PIP}]_S + S_{\mathrm{PM}}k^{\mathrm{PLC}}_{-\mathrm{PIP}}[\mathrm{PLC}-\mathrm{G_{q\alpha}GTP}-\mathrm{PIP}]_S}_{\mathrm{PLC\ binding\ PIP}}$$
$$\underbrace{+S_{\mathrm{PM}}k^{\mathrm{PLC}}_{\mathrm{PIP,cat}}[\mathrm{PLC}-\mathrm{G_{q\alpha}GTP}-\mathrm{PIP}]_S}_{\mathrm{Formation\ of\ IP_2}}$$
$$\underbrace{-S_{\mathrm{PM}}\Big(\frac{S_{\mathrm{PM}}}{V_{\mathrm{cyt}}}\Big)k^{\mathrm{PLC}}_{\mathrm{PIP_2}}[\mathrm{PLC}-\mathrm{G_{q\alpha}GTP}]_S[\mathrm{PIP_2}]_S + S_{\mathrm{PM}}k^{\mathrm{PLC}}_{-\mathrm{PIP_2}}[\mathrm{PLC}-\mathrm{G_{q\alpha}GTP}-\mathrm{PIP_2}]_S}_{\mathrm{PLC\ binding\ PIP_2}}$$
$$\underbrace{+S_{\mathrm{PM}}k^{\mathrm{PLC}}_{\mathrm{PIP_2,cat}}[\mathrm{PLC}-\mathrm{G_{q\alpha}GTP}-\mathrm{PIP_2}]_S}_{\mathrm{Formation\ of\ IP_3}} \quad (44)$$

$$S_{\mathrm{PM}}\frac{d}{dt}[\mathrm{PLC}-\mathrm{G_{q\alpha}GDP}]_S = \underbrace{S_{\mathrm{PM}}k^{\mathrm{PLC}}_{\mathrm{hydrolyze}}[\mathrm{PLC}-\mathrm{G_{q\alpha}GTP}]_S}_{\mathrm{GTP\ hydrolysis\ (inactivation)}}$$
$$\underbrace{-S_{\mathrm{PM}}k^{\mathrm{PLC}}_{-\mathrm{G_{q\alpha}GDP}}[\mathrm{PLC}-\mathrm{G_{q\alpha}GDP}]_S + S_{\mathrm{PM}}k^{\mathrm{PLC}}_{\mathrm{G_{q\alpha}GDP}}[\mathrm{PLC}]_S[\mathrm{G_{q\alpha}GDP}]_S}_{G_{q\alpha}\ \mathrm{GDP\ binding}} \quad (45)$$

$$S_{\mathrm{PM}}\frac{d}{dt}[\mathrm{PLC}-\mathrm{G_{q\alpha}GTP}-\mathrm{PI}]_S = \underbrace{S_{\mathrm{PM}}\Big(\frac{S_{\mathrm{PM}}}{V_{\mathrm{cyt}}}\Big)k^{\mathrm{PLC}}_{\mathrm{PI}}[\mathrm{PLC}-\mathrm{G_{q\alpha}GTP}]_S[\mathrm{PI}]_S - S_{\mathrm{PM}}k^{\mathrm{PLC}}_{-\mathrm{PI}}[\mathrm{PLC}-\mathrm{G_{q\alpha}GTP}-\mathrm{PI}]_S}_{\mathrm{PLC\ binding\ PI}}$$
$$\underbrace{-S_{\mathrm{PM}}k^{\mathrm{PLC}}_{\mathrm{PI,cat}}[\mathrm{PLC}-\mathrm{G_{q\alpha}GTP}-\mathrm{PI}]_S}_{\mathrm{Formation\ of\ I_1P}} \quad (46)$$

$$S_{\mathrm{PM}}\frac{d}{dt}[\mathrm{PLC}-\mathrm{G_{q\alpha}GTP}-\mathrm{PIP}]_S = \underbrace{S_{\mathrm{PM}}\Big(\frac{S_{\mathrm{PM}}}{V_{\mathrm{cyt}}}\Big)k^{\mathrm{PLC}}_{\mathrm{PIP}}[\mathrm{PLC}-\mathrm{G_{q\alpha}GTP}]_S[\mathrm{PIP}]_S - S_{\mathrm{PM}}k^{\mathrm{PLC}}_{-\mathrm{PIP}}[\mathrm{PLC}-\mathrm{G_{q\alpha}GTP}-\mathrm{PIP}]_S}_{\text{PLC binding PI}} \underbrace{-S_{\mathrm{PM}}k^{\mathrm{PLC}}_{\mathrm{PIP,cat}}[\mathrm{PLC}-\mathrm{G_{q\alpha}GTP}-\mathrm{PIP}]_S}_{\text{Formation of IP}_2} \tag{47}$$

$$S_{\mathrm{PM}}\frac{d}{dt}[\mathrm{PLC}-\mathrm{G_{q\alpha}GTP}-\mathrm{PIP_2}]_S = \underbrace{S_{\mathrm{PM}}\Big(\frac{S_{\mathrm{PM}}}{V_{\mathrm{cyt}}}\Big)k^{\mathrm{PLC}}_{\mathrm{PIP_2}}[\mathrm{PLC}-\mathrm{G_{q\alpha}GTP}]_S[\mathrm{PIP_2}]_S - S_{\mathrm{PM}}k^{\mathrm{PLC}}_{-\mathrm{PIP_2}}[\mathrm{PLC}-\mathrm{G_{q\alpha}GTP}-\mathrm{PIP_2}]_S}_{\text{PLC binding PIP}_2} \underbrace{-S_{\mathrm{PM}}k^{\mathrm{PLC}}_{\mathrm{PIP_2,cat}}[\mathrm{PLC}-\mathrm{G_{q\alpha}GTP}-\mathrm{PIP_2}]_S}_{\text{Formation of IP}_3} \tag{48}$$

### S4.5.3 PKC

PKC exists in the cytosol at approximately the same copy number as CalDAG; its concentration in this model is controlled by the volume clustering coefficient $\beta_v$. PKC inactivates $P2Y_1$ through phosphorylation [11]

| Parameter | Value | Units | Reference |
|---|---|---|---|
| $k_{\mathrm{Ca}}^{\mathrm{PKC}}$ | $6 \cdot 10^5$ | 1/M-s | [2] |
| $k_{-\mathrm{Ca}}^{\mathrm{PKC}}$ | 0.5 | 1/s | [2] |
| $k_{\mathrm{DAG}}^{\mathrm{PKC-Ca}}$ | $8 \cdot 10^3$ | 1/M-s | [2] |
| $k_{-\mathrm{DAG}}^{\mathrm{PKC-Ca}}$ | 8.6348 | 1/s | [2] |
| $k_{\mathrm{act}}^{\mathrm{PKC}}$ | 1 | 1/s | [2] |
| $k_{-\mathrm{act}}^{\mathrm{PKC}}$ | 2 | 1/s | [2] |
| $k_{\mathrm{act}}^{\mathrm{PKC-Ca}}$ | 1.2706 | 1/M-s | [2] |
| $k_{-\mathrm{act}}^{\mathrm{PKC_{Ca}}}$ | 3.5026 | 1/s | [2] |
| $k_{\mathrm{act}}^{\mathrm{PKC-Ca-DAG}}$ | 1 | 1/M-s | [2] |
| $k_{-\mathrm{act}}^{\mathrm{PKC-Ca-DAG}}$ | 0.1 | 1/s | [2] |
| $N^{\mathrm{PKC}}$ | 36135 | # | [17] |

Tab. S13: Parameter values associated with PKC. At time $t = 0$ s in the steady-state simulation, $[\mathrm{PKC}] = N^{\mathrm{PKC}}/V_{\mathrm{cyt}}$ and all other states are initialized to 0.

$$V_{\mathrm{cyt}}\frac{d}{dt}[\mathrm{PKC}] = \underbrace{-V_{\mathrm{cyt}}k_{\mathrm{act}}^{\mathrm{PKC}}[\mathrm{PKC}] + V_{\mathrm{cyt}}k_{-\mathrm{act}}^{\mathrm{PKC}}[\mathrm{PKCa}]}_{\text{activation of PKC}} \underbrace{-V_{\mathrm{cyt}}k_{\mathrm{Ca}}^{\mathrm{PKC}}[\mathrm{PKC}][\mathrm{Ca}_{\mathrm{cyt}}] + V_{\mathrm{cyt}}k_{-\mathrm{Ca}}^{\mathrm{PKC}}[\mathrm{PKC-Ca}]}_{\text{Ca binding to PKC}} \tag{49}$$

$$V_{\mathrm{cyt}}\frac{d}{dt}[\mathrm{PKCa}] = \underbrace{V_{\mathrm{cyt}}k_{\mathrm{act}}^{\mathrm{PKC}}[\mathrm{PKC}] - V_{\mathrm{cyt}}k_{-\mathrm{act}}^{\mathrm{PKC}}[\mathrm{PKCa}]}_{\text{activation of PKC}} \underbrace{-V_{\mathrm{cyt}}k_{\mathrm{Ca}}^{\mathrm{PKC}}[\mathrm{PKCa}][\mathrm{Ca}_{\mathrm{cyt}}] + V_{\mathrm{cyt}}k_{-\mathrm{Ca}}^{\mathrm{PKC}}[\mathrm{PKCa-Ca}]}_{\text{Ca binding to PKCa}} \tag{50}$$

$$V_{\text{cyt}}\frac{d}{dt}[\text{PKC}-\text{Ca}] = \underbrace{-V_{\text{cyt}}k_{\text{act}}^{\text{PKC}-\text{Ca}}[\text{PKC}-\text{Ca}] + V_{\text{cyt}}k_{-\text{act}}^{\text{PKC}-\text{Ca}}[\text{PKCa}-\text{Ca}]}_{\text{activation of PKC-Ca}} \underbrace{+V_{\text{cyt}}k_{\text{Ca}}^{\text{PKC}}[\text{PKC}][\text{Ca}_{\text{cyt}}] - V_{\text{cyt}}k_{-\text{Ca}}^{\text{PKC}}[\text{PKC}-\text{Ca}]}_{\text{Ca binding to PKC}} \underbrace{-S_{\text{PM}}k_{\text{DAG}}^{\text{PKC}}[\text{PKC}-\text{Ca}][\text{DAG}]_S + V_{\text{cyt}}k_{-\text{DAG}}^{\text{PKC}}[\text{PKC}-\text{Ca}-\text{DAG}]}_{\text{DAG binding to PKC-Ca}} \tag{51}$$

$$V_{\text{cyt}}\frac{d}{dt}[\text{PKCa}-\text{Ca}] = \underbrace{V_{\text{cyt}}k_{\text{act}}^{\text{PKC}-\text{Ca}}[\text{PKC}-\text{Ca}] - V_{\text{cyt}}k_{-\text{act}}^{\text{PKC}-\text{Ca}}[\text{PKCa}-\text{Ca}]}_{\text{activation of PKC}} \underbrace{V_{\text{cyt}}k_{\text{Ca}}^{\text{PKC}}[\text{PKCa}][\text{Ca}_{\text{cyt}}] - V_{\text{cyt}}k_{-\text{Ca}}^{\text{PKC}}[\text{PKCa}-\text{Ca}]}_{\text{Ca binding to PKCa}} \underbrace{-S_{\text{PM}}k_{\text{DAG}}^{\text{PKC}}[\text{PKCa}-\text{Ca}][\text{DAG}]_S + V_{\text{cyt}}k_{-\text{DAG}}^{\text{PKC}}[\text{PKCa}-\text{Ca}-\text{DAG}]}_{\text{DAG binding to PKCa-Ca}} \tag{52}$$

$$V_{\text{cyt}}\frac{d}{dt}[\text{PKC}-\text{Ca}-\text{DAG}] = \underbrace{-V_{\text{cyt}}k_{\text{act}}^{\text{PKC}-\text{Ca}-\text{DAG}}[\text{PKC}-\text{Ca}-\text{DAG}] + V_{\text{cyt}}k_{-\text{act}}^{\text{PKC}-\text{Ca}-\text{DAG}}[\text{PKCa}-\text{Ca}-\text{DAG}]}_{\text{activation of PKC-Ca-DAG}} \underbrace{+S_{\text{PM}}k_{\text{DAG}}^{\text{PKC}}[\text{PKC}-\text{Ca}][\text{DAG}]_S - V_{\text{cyt}}k_{-\text{DAG}}^{\text{PKC}}[\text{PKC}-\text{Ca}-\text{DAG}]}_{\text{DAG binding to PKC-Ca}} \tag{53}$$

$$V_{\text{cyt}}\frac{d}{dt}[\text{PKCa}-\text{Ca}-\text{DAG}] = \underbrace{V_{\text{cyt}}k_{\text{act}}^{\text{PKC}-\text{Ca}-\text{DAG}}[\text{PKC}-\text{Ca}-\text{DAG}] - V_{\text{cyt}}k_{-\text{act}}^{\text{PKC}-\text{Ca}-\text{DAG}}[\text{PKCa}-\text{Ca}-\text{DAG}]}_{\text{activation of PKC-Ca-DAG}} \underbrace{+S_{\text{PM}}k_{\text{DAG}}^{\text{PKC}}[\text{PKCa}-\text{Ca}][\text{DAG}]_S - V_{\text{cyt}}k_{-\text{DAG}}^{\text{PKC}}[\text{PKCa}-\text{Ca}-\text{DAG}]}_{\text{DAG binding to PKCa-Ca}} \tag{54}$$

## S4.6 Calcium and Inositol Species

### S4.6.1 Inositol

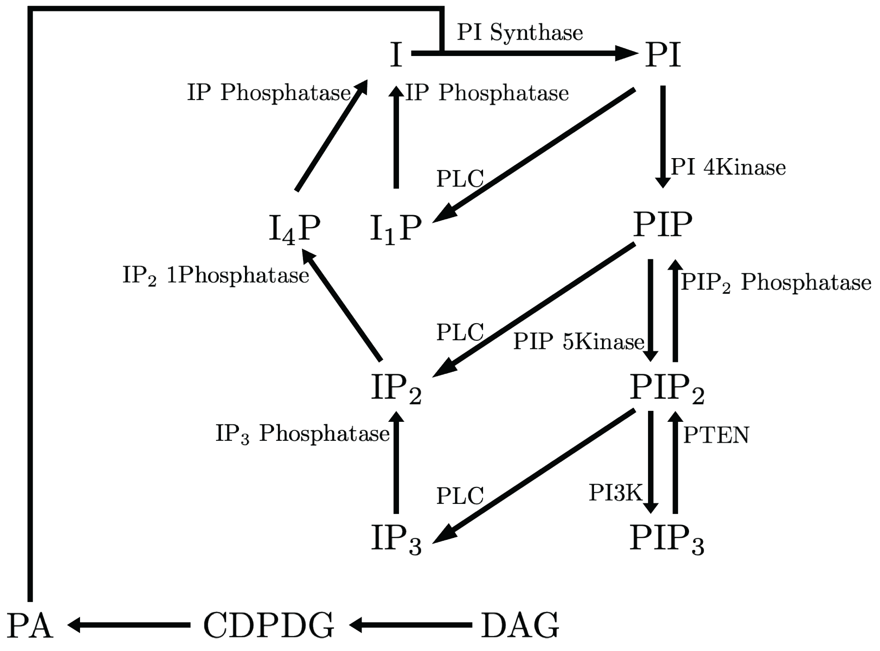


Fig. S3: Inositol reaction diagram

| Parameter | Value | Units | Reference |
|---|---|---|---|
| $k^{\text{PISynthase}}_{\text{I,cat}}$ | 13.6 | 1/s | [15] |
| $k^{\text{PI4Kinase}}_{\text{PI,cat}}$ | 2.77 | 1/s | [5] |
| $K^{\text{PI4Kinase}}_{\text{PI,M}}$ | $1.6 \cdot 10^{-5}$ | M | [5] |
| $k^{\text{PIP5Kinase}}_{\text{PIP,cat}}$ | 1.021 | 1/s | [14] |
| $K^{\text{PIP5Kinase}}_{\text{PIP,cat}}$ | $1. \cdot 10^{-5}$ | M | [14] |
| $k^{\text{PIP}_2\text{Phosphatase}}_{-\text{PIP}_2\text{,cat}}$ | 1 | 1/s | [8] |
| $K^{\text{PIP}_2\text{Phosphatase}}_{-\text{PIP}_2\text{,M}}$ | 0.00025 | M | [8] |
| $k^{\text{IPPhosphatase}}_{-\text{I}_1\text{P,cat}}$ | 1 | 1/s | [1] |
| $K^{\text{IPPhosphatase}}_{-\text{I}_1\text{P,M}}$ | 0.00012 | M | [1] |
| $k^{\text{IPPhosphatase}}_{-\text{I}_4\text{P,cat}}$ | 1 | 1/s | [1] |
| $K^{\text{IPPhosphatase}}_{-\text{I}_4\text{P,M}}$ | 0.00012 | M | [1] |
| $k^{\text{IP}_3\text{5Phosphatase}}_{-\text{IP}_3\text{,cat}}$ | 31.25 | 1/s | [9] |
| $K^{\text{IP}_3\text{5Phosphatase}}_{-\text{IP}_3\text{,M}}$ | $2.4 \cdot 10^{-5}$ | M | [9] |
| $k^{\text{IP}_2\text{1Phosphatase}}_{-\text{IP}_2\text{,cat}}$ | 0.05027 | 1/s | [10] |
| $K^{\text{IP}_2\text{1Phosphatase}}_{\text{IP}_2\text{,M}}$ | $9 \cdot 10^{-7}$ | M | [10] |
| $k^{\text{PTEN}}_{-\text{PIP}_3\text{,cat}}$ | See Table S2 | 1/s | |
| $K^{\text{PTEN}}_{-\text{PIP}_3\text{,m}}$ | See Table S2 | M | |
| $k^{\text{DAGKinase}}_{\text{DAG,cat}}$ | 0.2618 | 1/s | [16] |
| $K^{\text{DAGKinase}}_{\text{DAG,M}}$ | 0.00025 | M | [16] |
| $k^{\text{CDPDGSynthase}}_{\text{PA,cat}}$ | 8.917 | 1/s | [6] |
| $K^{\text{CDPDGSynthase}}_{\text{PA,M}}$ | 0.0005 | M | [6] |
| $K^{\text{CDPDGSynthase}}_{\text{CTP,M}}$ | 0.001 | M | [6] |
| $K^{\text{PISynthase}}_{\text{I,M}}$ | $1.3 \cdot 10^{-5}$ | M | [15] |
| $K^{\text{PISynthase}}_{\text{CDPDG,M}}$ | 0.00028 | M | [15] |
| [CTP] | 0.000278 | M | [12] |
| [PISynthase] | $1.482896 \cdot 10^{-6}$ | M | [17] |
| [PI4Kinase] | $491.53105 \cdot 10^{-9}$ | M | [17] |
| [PIP5Kinase] | $329.3479 \cdot 10^{-9}$ | M | [17] |
| [$\text{PIP}_2$Phosphatase] | $698.273 \cdot 10^{-9}$ | M | [17] |
| [IPPhosphatase] | $4.55939 \cdot 10^{-6}$ | M | [17] |
| [$\text{IP}_3$5Phosphatase] | $698.273 \cdot 10^{-9}$ | M | [17] |
| [$\text{IP}_2$1Phosphatase] | $2.420569 \cdot 10^{-6}$ | M | [17] |
| [DGKinase] | $692.737739 \cdot 10^{-9}$ | M | [17] |
| [CDPDGSynthase] | $2.159028 \cdot 10^{-6}$ | M | [17] |
| [PTEN] | $100.1882 \cdot 10^{-9}$ | M | [17] |
| $N^{\text{Ins}}$ | 291460 | # | |

Tab. S14: Parameter values associated with inositol. At time $t = 0$ s in the steady-state simulation, $[\text{I}]_S = N_{\text{Ins}}/S_{\text{PM}}$ and all other states are initialized to 0.

$$S_{\mathrm{PM}}\frac{d}{dt}[\mathrm{PI}]_S = S_{\mathrm{PM}} \underbrace{\frac{\left(\frac{S_{\mathrm{PM}}}{V_{\mathrm{cyt}}}\right)k^{\mathrm{PISynthase}}_{\mathrm{I,cat}}[\mathrm{PISynthase}][\mathrm{CDPDG}]_S}{K^{\mathrm{PISynthase}}_{\mathrm{CDPDG,M}}K^{\mathrm{PISynthase}}_{\mathrm{I,M}} + K^{\mathrm{PISynthase}}_{\mathrm{CDPDG,M}}[\mathrm{I}] + (K^{\mathrm{PISynthase}}_{\mathrm{I,M}}[\mathrm{CDPDG}]_S + [\mathrm{I}][\mathrm{CDPDG}]_S)\left(\frac{S_{\mathrm{PM}}}{V_{\mathrm{cyt}}}\right)}[\mathrm{I}]}_{\text{Synthesis of PI}}$$

$$\underbrace{-V_{\mathrm{cyt}}\frac{k^{\mathrm{PI4Kinase}}_{\mathrm{PI,cat}}[\mathrm{PI4Kinase}]}{\left(\frac{V_{\mathrm{cyt}}}{S_{\mathrm{PM}}}\right)K^{\mathrm{PI4Kinase}}_{\mathrm{PI,M}} + [\mathrm{PI}]_S}[\mathrm{PI}]_S}_{\text{Synthesis of PIP}} \tag{55}$$

$$\underbrace{-S_{\mathrm{PM}}\left(\frac{S_{\mathrm{PM}}}{V_{\mathrm{cyt}}}\right)k^{\mathrm{PLC}}_{\mathrm{PI}}[\mathrm{PLC}-\mathrm{G_{q\alpha}GTP}]_S[\mathrm{PI}]_S + S_{\mathrm{PM}}k^{\mathrm{PLC}}_{-\mathrm{PI}}[\mathrm{PLC}-\mathrm{G_{q\alpha}GTP}-\mathrm{PI}]_S}_{\text{PLC binding PI}}$$

$$S_{\mathrm{PM}}\frac{d}{dt}[\mathrm{PIP}]_S = \underbrace{V_{\mathrm{cyt}}\frac{k^{\mathrm{PI4Kinase}}_{\mathrm{PI,cat}}[\mathrm{PI4Kinase}]}{\left(\frac{V_{\mathrm{cyt}}}{S_{\mathrm{PM}}}\right)K^{\mathrm{PI4Kinase}}_{\mathrm{PI,M}} + [\mathrm{PI}]_S}[\mathrm{PI}]_S}_{\text{Synthesis of PIP}} \underbrace{-V_{\mathrm{cyt}}\frac{k^{\mathrm{PIP5Kinase}}_{\mathrm{PIP,cat}}[\mathrm{PIP5Kinase}]}{\left(\frac{V_{\mathrm{cyt}}}{S_{\mathrm{PM}}}\right)K^{\mathrm{PIP5Kinase}}_{\mathrm{PIP,M}} + [\mathrm{PIP}]_S}[\mathrm{PIP}]_S}_{\text{Synthesis of PIP}_2}$$

$$\underbrace{+V_{\mathrm{cyt}}\frac{k^{\mathrm{PIP_2Phosphatase}}_{\mathrm{PIP_2,cat}}[\mathrm{PIP_2Phosphatase}]}{\left(\frac{V_{\mathrm{cyt}}}{S_{\mathrm{PM}}}\right)K^{\mathrm{PIP_2Phosphatase}}_{\mathrm{PIP_2,M}} + [\mathrm{PIP_2}]_S}[\mathrm{PIP_2}]_S}_{\text{Dephosphorylation of PIP}_2} \tag{56}$$

$$\underbrace{-S_{\mathrm{PM}}\left(\frac{S_{\mathrm{PM}}}{V_{\mathrm{cyt}}}\right)k^{\mathrm{PLC}}_{\mathrm{PIP}}[\mathrm{PLC}-\mathrm{G_{q\alpha}GTP}]_S[\mathrm{PIP}]_S + S_{\mathrm{PM}}k^{\mathrm{PLC}}_{-\mathrm{PIP}}[\mathrm{PLC}-\mathrm{G_{q\alpha}GTP}-\mathrm{PIP}]_S}_{\text{PLC binding PIP}}$$

$$S_{\mathrm{PM}}\frac{d}{dt}[\mathrm{PIP_2}]_S = \underbrace{V_{\mathrm{cyt}}\frac{k^{\mathrm{PIP5Kinase}}_{\mathrm{PIP}}[\mathrm{PIP5Kinase}]}{\left(\frac{V_{\mathrm{cyt}}}{S_{\mathrm{PM}}}\right)K^{\mathrm{PIP5Kinase}}_{\mathrm{PIP,M}} + [\mathrm{PIP}]_S}[\mathrm{PIP}]_S}_{\text{Synthesis of PIP}_2} \underbrace{-V_{\mathrm{cyt}}\frac{k^{\mathrm{PIP_2Phosphatase}}_{\mathrm{PIP_2}}[\mathrm{PIP_2Phosphatase}]}{\left(\frac{V_{\mathrm{cyt}}}{S_{\mathrm{PM}}}\right)K^{\mathrm{PIP_2Phosphatase}}_{\mathrm{PIP_2,M}} + [\mathrm{PIP_2}]_S}[\mathrm{PIP_2}]_S}_{\text{Dephosphorylation of PIP}_2}$$

$$\underbrace{+V_{\mathrm{cyt}}\frac{k^{\mathrm{PTEN}}_{\mathrm{PIP_3}}[\mathrm{PTEN}]}{\left(\frac{V_{\mathrm{cyt}}}{S_{\mathrm{PM}}}\right)K^{\mathrm{PTEN}}_{\mathrm{PIP_3,M}} + [\mathrm{PIP_3}]_S}[\mathrm{PIP_3}]_S}_{\text{PIP}_3\text{ degredation}}$$

$$\underbrace{\begin{aligned}&-S_{\mathrm{PM}}\left(\frac{S_{\mathrm{PM}}}{V_{\mathrm{cyt}}}\right)k^{\mathrm{PLC}}_{\mathrm{PIP_2}}[\mathrm{PLC}-\mathrm{G_{q\alpha}GTP}]_S[\mathrm{PIP_2}]_S\\ &\quad +S_{\mathrm{PM}}k^{\mathrm{PLC}}_{-\mathrm{PIP_2}}[\mathrm{PLC}-\mathrm{G_{q\alpha}GTP}-\mathrm{PIP_2}]_S\end{aligned}}_{\text{PLC binding PIP}_2} \tag{57}$$

$$\underbrace{\begin{aligned}&-S_{\mathrm{PM}}\left(\frac{S_{\mathrm{PM}}}{V_{\mathrm{cyt}}}\right)k^{\mathrm{PI3K}}_{\mathrm{PIP_2}}[\mathrm{PI3K}-\mathrm{G_{i\alpha}GTP}]_S[\mathrm{PIP_2}]_S\\ &\quad +S_{\mathrm{PM}}k^{\mathrm{PI3K}}_{-\mathrm{PIP_2}}[\mathrm{PI3K}-\mathrm{G_{i\alpha}GTP}-\mathrm{PIP_2}]_S\end{aligned}}_{\text{PI3K binding PIP}_2}$$

$$V_{\mathrm{cyt}}\frac{d}{dt}[\mathrm{I_1P}] = \underbrace{S_{\mathrm{PM}}k^{\mathrm{PLC}}_{\mathrm{PI,cat}}[\mathrm{PLC-G_{q\alpha}GTP-PI}]_S}_{\text{Formation of } \mathrm{I_1P}} \underbrace{-V_{\mathrm{cyt}}\frac{k^{\mathrm{IPPhosphatase}}_{\mathrm{I_1P,cat}}[\mathrm{IPPhosphatase}]}{K^{\mathrm{IPPhosphatase}}_{\mathrm{I_1P,M}}+[\mathrm{I_1P}]}[\mathrm{I_1P}]}_{\text{Dephosphorylation of } \mathrm{I_1P}} \tag{58}$$

$$V_{\mathrm{cyt}}\frac{d}{dt}[\mathrm{I_4P}] = \underbrace{V_{\mathrm{cyt}}\frac{k^{\mathrm{IP_2Phosphatase}}_{\mathrm{IP_2,cat}}[\mathrm{IP_2Phosphatase}]}{K^{\mathrm{IP_2Phosphatase}}_{\mathrm{IP_2,M}}+[\mathrm{IP_2}]}[\mathrm{IP_2}]}_{\text{Dephosphorylation of } \mathrm{IP_2}} \underbrace{-V_{\mathrm{cyt}}\frac{k^{\mathrm{IPPhosphatase}}_{\mathrm{I_4P,cat}}[\mathrm{IPPhosphatase}]}{K^{\mathrm{IPPhosphatase}}_{\mathrm{I_4P,M}}+[\mathrm{I_4P}]}[\mathrm{I_4P}]}_{\text{Dephosphorylation of } \mathrm{I_4P}} \tag{59}$$

$$\begin{aligned} V_{\mathrm{cyt}}\frac{d}{dt}[\mathrm{IP_2}] = &\underbrace{S_{\mathrm{PM}}k^{\mathrm{PLC}}_{\mathrm{PIP,cat}}[\mathrm{PLC-G_{q\alpha}GTP-PIP}]_S}_{\text{Formation of } \mathrm{IP_2}} \underbrace{-V_{\mathrm{cyt}}\frac{k^{\mathrm{IP_2Phosphatase}}_{\mathrm{IP_2,cat}}[\mathrm{IP_2Phosphatase}]}{K^{\mathrm{IP_2Phosphatase}}_{\mathrm{IP_2,M}}+[\mathrm{IP_2}]}[\mathrm{IP_2}]}_{\text{Dephosphorylation of } \mathrm{IP_2}} \\ &\underbrace{+V_{\mathrm{cyt}}\frac{k^{\mathrm{IP_35Phosphatase}}_{\mathrm{IP_3,cat}}[\mathrm{IP_35Phosphatase}]}{K^{\mathrm{IP_35Phosphatase}}_{\mathrm{IP_3,M}}+[\mathrm{IP_3}]}[\mathrm{IP_3}]}_{\text{Dephosphorylation of } \mathrm{IP_3}} \end{aligned} \tag{60}$$

$$\begin{aligned} V_{\mathrm{cyt}}\frac{d}{dt}[\mathrm{I}] = &\underbrace{V_{\mathrm{cyt}}\frac{k^{\mathrm{IPPhosphatase}}_{\mathrm{I_1P,cat}}[\mathrm{IPPhosphatase}]}{K^{\mathrm{IPPhosphatase}}_{\mathrm{I_1P,M}}+[\mathrm{I_1P}]}[\mathrm{I_1P}]}_{\text{Dephosphorylation of } \mathrm{I_1P}} \underbrace{+V_{\mathrm{cyt}}\frac{k^{\mathrm{IPPhosphatase}}_{\mathrm{I_4P,cat}}[\mathrm{IPPhosphatase}]}{K^{\mathrm{IPPhosphatase}}_{\mathrm{I_4P,M}}+[\mathrm{I_4P}]}[\mathrm{I_4P}]}_{\text{Dephosphorylation of } \mathrm{I_4P}} \\ &- S_{\mathrm{PM}}\underbrace{\frac{\left(\frac{S_{\mathrm{PM}}}{V_{\mathrm{cyt}}}\right)k^{\mathrm{PISynthase}}_{\mathrm{I,cat}}[\mathrm{PISynthase}][\mathrm{CDPDG}]_S}{K^{\mathrm{PISynthase}}_{\mathrm{CDPDG,M}}K^{\mathrm{PISynthase}}_{\mathrm{I,M}}+K^{\mathrm{PISynthase}}_{\mathrm{CDPDG,M}}[\mathrm{I}]+(K^{\mathrm{PISynthase}}_{\mathrm{I,M}}[\mathrm{CDPDG}]_S+[\mathrm{I}][\mathrm{CDPDG}]_S)\left(\frac{S_{\mathrm{PM}}}{V_{\mathrm{cyt}}}\right)}[\mathrm{I}]}_{\text{Synthesis of PI}} \end{aligned} \tag{61}$$

$$\begin{aligned} V_{\mathrm{cyt}}\frac{d}{dt}[\mathrm{IP_3}] = &\underbrace{S_{\mathrm{PM}}k^{\mathrm{PLC}}_{\mathrm{PIP_2,cat}}[\mathrm{PLC-G_{q\alpha}GTP-PIP_2}]_S}_{\text{Formation of } \mathrm{IP_3}} \underbrace{-V_{\mathrm{cyt}}\frac{k^{\mathrm{IP_35Phosphatase}}_{\mathrm{IP_3}}[\mathrm{IP_35Phosphatase}]}{K^{\mathrm{IP_35Phosphatase}}_{\mathrm{IP_3,M}}+[\mathrm{IP_3}]}[\mathrm{IP_3}]}_{\text{Dephosphorylation of } \mathrm{IP_3}} \\ &\underbrace{-S_{\mathrm{IM}}\frac{(k_2L_3+l_4)[\mathrm{Ca^{2+}_{cyt}}]}{L_3+[\mathrm{Ca^{2+}_{cyt}}](1+L_1/L_3)}[\mathrm{IP_3R_n}]_S[\mathrm{IP_3}]+S_{\mathrm{IM}}\frac{k_{-2}+l_{-4}[\mathrm{Ca^{2+}_{cyt}}]}{1+[\mathrm{Ca^{2+}_{cyt}}]/L_5}[\mathrm{IP_3R_o}]_S}_{\mathrm{IP_3}\text{ binding to }\mathrm{IP_3}\text{ receptor}} \end{aligned} \tag{62}$$

$$\begin{aligned} S_{\mathrm{PM}}\frac{d}{dt}[\mathrm{PIP_3}]_S = &\underbrace{S_{\mathrm{PM}}k^{\mathrm{PI3K}}_{\mathrm{PIP_2,cat}}[\mathrm{PI3K-G_{i\alpha}GTP-PIP_2}]_S}_{\mathrm{PIP_3}\text{ generation}} \underbrace{-V_{\mathrm{cyt}}\frac{k^{\mathrm{PTEN}}_{\mathrm{PIP_3,cat}}[\mathrm{PTEN}]}{\left(\frac{V_{\mathrm{cyt}}}{S_{\mathrm{PM}}}\right)K^{\mathrm{PTEN}}_{\mathrm{PIP_3,M}}+[\mathrm{PIP_3}]_S}[\mathrm{PIP_3}]_S}_{\mathrm{PIP_3}\text{ degradation}} \\ &\underbrace{\begin{aligned}&+S_{\mathrm{PM}}k^{\mathrm{RASA3}}_{-\mathrm{PIP_3}}[\mathrm{RASA3-PIP_3}]_S \\ &\quad -S_{\mathrm{PM}}\left(\frac{S_{\mathrm{PM}}}{V_{\mathrm{cyt}}}\right)k^{\mathrm{RASA3}}_{\mathrm{PIP_3}}[\mathrm{RASA3}]_S[\mathrm{PIP_3}]_S\end{aligned}}_{\mathrm{PIP_3}\text{ inactivation of RASA3}} \end{aligned} \tag{63}$$

$$
\begin{aligned}
S_{\mathrm{PM}}\frac{d}{dt}[\mathrm{DAG}]_S = {} & \underbrace{S_{\mathrm{PM}} k_{\mathrm{PIP_2,cat}}^{\mathrm{PLC}}[\mathrm{PLC-G_{q\alpha}GTP-PIP_2}]_S}_{\text{Conversion to IP}_3} \underbrace{+S_{\mathrm{PM}} k_{\mathrm{PIP,cat}}^{\mathrm{PLC}}[\mathrm{PLC-G_{q\alpha}GTP-PIP}]_S}_{\text{Conversion to IP}_2} \\
& \underbrace{+S_{\mathrm{PM}} k_{\mathrm{PI,cat}}^{\mathrm{PLC}}[\mathrm{PLC-G_{q\alpha}GTP-PI}]_S}_{\text{Conversion to I}_1\text{P}} \underbrace{-V_{\mathrm{cyt}}\frac{k_{\mathrm{DAG,cat}}^{\mathrm{DAGKinase}}[\mathrm{DAGKinase}]}{\left(\frac{V_{\mathrm{cyt}}}{S_{\mathrm{PM}}}\right)K_{\mathrm{DAG,M}}^{\mathrm{DAGKinase}}+[\mathrm{DAG}]_S}[\mathrm{DAG}]_S}_{\text{Phosphorylation of DAG}} \\
& \underbrace{-S_{\mathrm{PM}} k_{\mathrm{DAG}}^{\mathrm{PKC-Ca}}[\mathrm{PKC-Ca}][\mathrm{DAG}]_S + V_{\mathrm{cyt}} k_{\mathrm{-DAG}}^{\mathrm{PKC-Ca}}[\mathrm{PKC-Ca-DAG}]}_{\text{DAG binding to PKC-Ca}} \\
& \underbrace{-S_{\mathrm{PM}} k_{\mathrm{DAG}}^{\mathrm{PKC-Ca}}[\mathrm{PKCa-Ca}][\mathrm{DAG}]_S + V_{\mathrm{cyt}} k_{\mathrm{-DAG}}^{\mathrm{PKC-Ca}}[\mathrm{PKCa-Ca-DAG}]}_{\text{DAG binding to PKCa-Ca}}
\end{aligned}
\tag{64}
$$

$$
\begin{aligned}
S_{\mathrm{PM}}\frac{d}{dt}[\mathrm{PA}]_S = {} & \underbrace{V_{\mathrm{cyt}}\frac{k_{\mathrm{DAG,cat}}^{\mathrm{DAGKinase}}[\mathrm{DAGKinase}]}{\left(\frac{V_{\mathrm{cyt}}}{S_{\mathrm{PM}}}\right)K_{\mathrm{DAG,M}}^{\mathrm{DAGKinase}}+[\mathrm{DAG}]_S}[\mathrm{DAG}]_S}_{\text{Phosphorylation of DAG}} \\
& -S_{\mathrm{PM}}\underbrace{\frac{k_{\mathrm{PA,cat}}^{\mathrm{CDPDGSynthase}}[\mathrm{CDPDGSynthase}][\mathrm{CTP}]}{K_{\mathrm{PA,M}}^{\mathrm{CDPDGSynthase}}K_{\mathrm{CTP,M}}^{\mathrm{CDPDGSynthase}}+K_{\mathrm{PA,M}}^{\mathrm{CDPDGSynthase}}[\mathrm{CTP}]+(K_{\mathrm{CTP,M}}^{\mathrm{CDPDGSynthase}}[\mathrm{PA}]_S+[\mathrm{PA}]_S[\mathrm{CTP}])\left(\frac{S_{\mathrm{PM}}}{V_{\mathrm{cyt}}}\right)}[\mathrm{PA}]_S}_{\text{Synthesis of CDPDG}}
\end{aligned}
\tag{65}
$$

$$
\begin{aligned}
S_{\mathrm{PM}}\frac{d}{dt}[\mathrm{CDPDG}]_S = {} & S_{\mathrm{PM}}\underbrace{\frac{k_{\mathrm{PA,cat}}^{\mathrm{CDPDGSynthase}}[\mathrm{CDPDGSynthase}][\mathrm{CTP}]}{K_{\mathrm{PA,M}}^{\mathrm{CDPDGSynthase}}K_{\mathrm{CTP,M}}^{\mathrm{CDPDGSynthase}}+K_{\mathrm{PA,M}}^{\mathrm{CDPDGSynthase}}[\mathrm{CTP}]+(K_{\mathrm{CTP,M}}^{\mathrm{CDPDGSynthase}}[\mathrm{PA}]_S+[\mathrm{PA}]_S[\mathrm{CTP}])\left(\frac{S_{\mathrm{PM}}}{V_{\mathrm{cyt}}}\right)}[\mathrm{PA}]_S}_{\text{Synthesis of CDPDG}} \\
& -S_{\mathrm{PM}}\underbrace{\frac{\left(\frac{S_{\mathrm{PM}}}{V_{\mathrm{cyt}}}\right)k_{\mathrm{I,cat}}^{\mathrm{PISynthase}}[\mathrm{PISynthase}][\mathrm{CDPDG}]_S}{K_{\mathrm{CDPDG,M}}^{\mathrm{PISynthase}}K_{\mathrm{I,M}}^{\mathrm{PISynthase}}+K_{\mathrm{CDPDG,M}}^{\mathrm{PISynthase}}[\mathrm{I}]+(K_{\mathrm{I,M}}^{\mathrm{PISynthase}}[\mathrm{CDPDG}]_S+[\mathrm{I}][\mathrm{CDPDG}]_S)\left(\frac{S_{\mathrm{PM}}}{V_{\mathrm{cyt}}}\right)}[\mathrm{I}]}_{\text{Synthesis of PI}}
\end{aligned}
\tag{66}
$$

### S4.6.2 Calcium

$R$ is the ideal gas constant, $T$ is temperature, $z$ is the charge of a Calcium ion, and $F$ is the Faraday constant. To calculate the flux of calcium into the cytosol, the Nernst Potentials across the two membranes of our models are needed:

$$\psi_{\mathrm{IM}} = \frac{RT}{zF}\ln\left(\frac{[\mathrm{Ca_{dts}}]}{[\mathrm{Ca_{cyt}}]}\right) \qquad \psi_{\mathrm{PM}} = \frac{RT}{zF}\ln\left(\frac{[\mathrm{Ca_{prp}}]}{[\mathrm{Ca_{cyt}}]}\right)$$

| Parameter | Value | Units | Reference |
|---|---|---|---|
| $\gamma^{\mathrm{IP_3R}}$ | See Table S1 | S | |
| $\gamma^{\mathrm{leak}}$ | See Table S1 | S/dm$^2$ | |
| $k_{\mathrm{Ca}}^{\mathrm{Fura}}$ | $6 \cdot 10^{8}$ | 1/M-s | |
| $k_{-\mathrm{Ca}}^{\mathrm{Fura}}$ | 180 | 1/s | |

Tab. S15: Parameter values associated with calcium. At time $t = 0$ s in the steady-state simulation, $[\mathrm{Ca^{2+}_{PRP}}] = 1 \cdot 10^{-3}$ M, $[\mathrm{Ca^{2+}_{cyt}}] = 40 \cdot 10^{-9}$ M, $[\mathrm{Ca^{2+}_{DTS}}] = 100 \cdot 10^{-6}$ M, $[\mathrm{Fura-Ca}] = 0$ M. At each time step, we compute $[\mathrm{Fura}] = 5 \cdot 10^{-6} - [\mathrm{Fura-Ca}]$. Fura (the fluorescent calcium probe) is only considered during the parameter estimation of calcium data. For all other cases, $k_{\mathrm{Ca}}^{\mathrm{Fura}}\mathrm{Fura} = 0$.

$$V_{\mathrm{cyt}}\frac{d}{dt}[\mathrm{Fura-Ca}] = \underbrace{V_{\mathrm{cyt}}k_{\mathrm{Ca}}^{\mathrm{Fura}}[\mathrm{Fura}][\mathrm{Ca^{2+}_{cyt}}] - V_{\mathrm{cyt}}k_{-\mathrm{Ca}}^{\mathrm{Fura}}[\mathrm{Fura-Ca}]}_{\text{Calcium binding to calcium probe}} \tag{67}$$

$$\begin{aligned} V_{\mathrm{DTS}}\frac{d}{dt}[\mathrm{Ca^{2+}_{DTS}}] = {} & \underbrace{2S_{\mathrm{IM}}k_{5\mathrm{f}}^{\mathrm{SERCA}}[\mathrm{SERCA_{E2}PCa_2}]_S - 2S_{\mathrm{IM}}k_{5\mathrm{r}}^{\mathrm{SERCA}}[\mathrm{SERCA_{E2}P}]_S[\mathrm{Ca^{2+}_{DTS}}]}_{2\mathrm{Ca^{2+}_{DTS}}\text{ ions binding to SERCA}} \\ & \underbrace{-\frac{N^{\mathrm{IP_3R}}}{4}\frac{\gamma^{\mathrm{IP_3R}}}{zF}\left(\frac{0.1[\mathrm{IP_3R_o}]_S + 0.9[\mathrm{IP_3R_a}]_S}{[\mathrm{IP_3R}]_{\mathrm{S,tot}}}\right)^4\psi_{\mathrm{IM}}}_{\text{Calcium release from IP}_3\text{R}} \end{aligned} \tag{68}$$

$$\begin{aligned} V_{\mathrm{PRP}}\frac{d}{dt}[\mathrm{Ca^{2+}_{PRP}}] = {} & \underbrace{2S_{\mathrm{PM}}k_{5\mathrm{f}}^{\mathrm{PMCA}}[\mathrm{PMCA_{E2}PCa_2}]_S - 2S_{\mathrm{PM}}k_{5\mathrm{r}}^{\mathrm{PMCA}}[\mathrm{PMCA_{E2}P}]_S[\mathrm{Ca^{2+}_{PRP}}]}_{2\mathrm{Ca^{2+}_{PRP}}\text{ ions binding to PMCA}} \\ & \underbrace{-S_{\mathrm{PM}}\frac{\gamma^{\mathrm{leak}}}{zF}\psi_{\mathrm{PM}}}_{\text{Calcium leak into cytoplasm}} \end{aligned} \tag{69}$$

$$
\begin{aligned}
V_{\mathrm{cyt}}\frac{d}{dt}[\mathrm{Ca}^{2+}_{\mathrm{cyt}}] = {} & \underbrace{\frac{N_{\mathrm{IP_3R}}}{4}\frac{\gamma^{\mathrm{IP_3R}}}{zF}\Big(\frac{0.1[\mathrm{IP_3R_o}]_S + 0.9[\mathrm{IP_3R_a}]_S}{[\mathrm{IP_3R}]_{\mathrm{S,tot}}}\Big)^4\psi_{\mathrm{IM}}}_{\text{Calcium release from IP}_3\text{R}} \quad \underbrace{+S_{\mathrm{PM}}\frac{\gamma^{\mathrm{leak}}}{zF}\psi_{\mathrm{PM}}}_{\text{Calcium leak into cytoplasm}} \\
& \underbrace{-S_{\mathrm{IM}}\frac{(k_1^{\mathrm{IP_3R}}L_1^{\mathrm{IP_3R}} + l_2^{\mathrm{IP_3R}})[\mathrm{Ca}^{2+}_{\mathrm{cyt}}]}{L_1^{\mathrm{IP_3R}} + [\mathrm{Ca}^{2+}_{\mathrm{cyt}}](1 + L_1^{\mathrm{IP_3R}}/L_3^{\mathrm{IP_3R}})}[\mathrm{IP_3R_n}]_S + S_{\mathrm{IM}}(k_{-1}^{\mathrm{IP_3R}} + l_{-2}^{\mathrm{IP_3R}})[\mathrm{IP_3R_{i1}}]_S}_{\text{Calcium inactivating resting IP}_3\text{R}} \\
& \underbrace{-S_{\mathrm{IM}}\frac{(k_4^{\mathrm{IP_3R}}L_5^{\mathrm{IP_3R}} + l_6^{\mathrm{IP_3R}})[\mathrm{Ca}^{2+}_{\mathrm{cyt}}]}{L_5^{\mathrm{IP_3R}} + [\mathrm{Ca}^{2+}_{\mathrm{cyt}}]}[\mathrm{IP_3R_o}]_S + S_{\mathrm{IM}}\frac{L_1^{\mathrm{IP_3R}}(k_{-4}^{\mathrm{IP_3R}} + l_{-6}^{\mathrm{IP_3R}})}{L_1^{\mathrm{IP_3R}} + [\mathrm{Ca}^{2+}_{\mathrm{cyt}}]}[\mathrm{IP_3R_a}]_S}_{\text{Calcium activating receptor}} \\
& \underbrace{-S_{\mathrm{IM}}\frac{k_1^{\mathrm{IP_3R}}L_1^{\mathrm{IP_3R}} + l_2^{\mathrm{IP_3R}}}{L_1^{\mathrm{IP_3R}} + [\mathrm{Ca}^{2+}_{\mathrm{cyt}}]}[\mathrm{IP_3R_a}]_S[\mathrm{Ca}^{2+}_{\mathrm{cyt}}] + S_{\mathrm{IM}}(k_{-1}^{\mathrm{IP_3R}} + l_{-2}^{\mathrm{IP_3R}})[\mathrm{IP_3R_{i2}}]_S}_{\text{Calcium inactivating active IP}_3\text{R}} \\
& \underbrace{-2S_{\mathrm{IM}}k_{\mathrm{2f}}^{\mathrm{SERCA}}[\mathrm{SERCA_{E1}}]_S[\mathrm{Ca}^{2+}_{\mathrm{cyt}}]^2 + 2S_{\mathrm{IM}}k_{\mathrm{2r}}^{\mathrm{SERCA}}[\mathrm{SERCA_{E1}} - 2\mathrm{Ca}]_S}_{2\text{Ca}_{\text{cyt}}\text{ ions binding to SERCA}} \\
& \underbrace{-2S_{\mathrm{IM}}k_{\mathrm{2f}}^{\mathrm{PMCA}}[\mathrm{PMCA_{E1}}]_S[\mathrm{Ca}^{2+}_{\mathrm{cyt}}]^2 + 2S_{\mathrm{IM}}k_{\mathrm{2r}}^{\mathrm{PMCA}}[\mathrm{PMCA_{E1}} - 2\mathrm{Ca}]_S}_{2\text{Ca}_{\text{cyt}}\text{ ions binding to PMCA}} \\
& \underbrace{-V_{\mathrm{cyt}}k_{\mathrm{Ca}}^{\mathrm{PKC}}[\mathrm{PKC}][\mathrm{Ca}^{2+}_{\mathrm{cyt}}] + V_{\mathrm{cyt}}k_{-\mathrm{Ca}}^{\mathrm{PKC}}[\mathrm{PKC} - \mathrm{Ca}]}_{\text{Ca binding to PKC}} \\
& \underbrace{-V_{\mathrm{cyt}}k_{\mathrm{Ca}}^{\mathrm{PKC}}[\mathrm{PKCa}][\mathrm{Ca}^{2+}_{\mathrm{cyt}}] + V_{\mathrm{cyt}}k_{-\mathrm{Ca}}^{\mathrm{PKC}}[\mathrm{PKCa} - \mathrm{Ca}]}_{\text{Ca binding to PKCa}} \\
& \underbrace{-V_{\mathrm{cyt}}k_{\mathrm{Ca}}^{\mathrm{CalDAG-GEFI}}[\mathrm{Ca}^{2+}_{\mathrm{cyt}}][\mathrm{CalDAG-GEFI}] + V_{\mathrm{cyt}}k_{-\mathrm{Ca}}^{\mathrm{CalDAG-GEFI}}[\mathrm{CalDAG-GEFI-Ca}]}_{\text{CalDAG-GEFI binding calcium}} \\
& \underbrace{-V_{\mathrm{cyt}}k_{\mathrm{Ca}}^{\mathrm{CalDAG-GEFI}}[\mathrm{Ca}^{2+}_{\mathrm{cyt}}][\mathrm{CalDAG-GEFI-Ca}] + V_{\mathrm{cyt}}k_{-\mathrm{Ca}}^{\mathrm{CalDAG-GEFI}}[\mathrm{CalDAG-GEFI-2Ca}]}_{\text{CalDAG-GEFI-Ca binding calcium}} \\
& - \underbrace{V_{\mathrm{cyt}}k_{\mathrm{Ca}}^{\mathrm{Fura}}[\mathrm{Ca2+}][\mathrm{Fura}] + V_{\mathrm{cyt}}k_{-\mathrm{Ca}}^{\mathrm{Fura}}[\mathrm{Fura} - \mathrm{Ca}]}_{\text{Fluorescent Probe binding calcium}}
\end{aligned}
\tag{70}
$$

## S4.7 Calcium channels/pumps

### S4.7.1 $IP_3R$

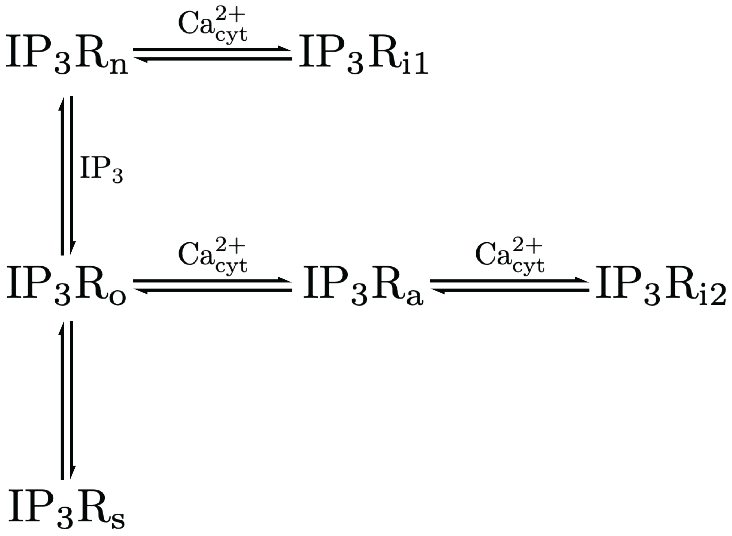


Fig. S4: $IP_3R$ reaction diagram. Model setup and states are exactly the same as in Sneyd and Dufour [13]

$$
\begin{aligned}
S_{IM}\frac{d}{dt}[IP_3R_n]_S = & \underbrace{-S_{IM}\frac{(k_1^{IP_3R}L_1^{IP_3R}+l_2^{IP_3R})[Ca^{2+}_{cyt}]}{L_1^{IP_3R}+[Ca^{2+}_{cyt}](1+L_1^{IP_3R}/L_3^{IP_3R})}[IP_3R_n]_S + S_{IM}(k_{-1}^{IP_3R}+l_{-2}^{IP_3R})[IP_3R_{i1}]_S}_{\text{Calcium inactivating resting IP}_3\text{R}} \\
& \underbrace{- S_{IM}\frac{k_2^{IP_3R}L_3^{IP_3R}+l_4^{IP_3R}[Ca^{2+}_{cyt}]}{L_3^{IP_3R}+[Ca^{2+}_{cyt}](1+L_3^{IP_3R}/L_1^{IP_3R})}[IP_3R_n]_S[IP_3] + S_{IM}\frac{k_{-2}^{IP_3R}+l_{-4}^{IP_3R}[Ca^{2+}_{cyt}]}{1+[Ca^{2+}_{cyt}]/L_5^{IP_3R}}[IP_3R_o]_S}_{\text{IP}_3\text{ binding to receptor}}
\end{aligned} \tag{71}
$$

$$
S_{IM}\frac{d}{dt}[IP_3R_{i1}]_S = \underbrace{S_{IM}\frac{(k_1^{IP_3R}L_1^{IP_3R}+l_2^{IP_3R})[Ca^{2+}_{cyt}]}{L_1^{IP_3R}+[Ca^{2+}_{cyt}](1+L_1^{IP_3R}/L_3^{IP_3R})}[IP_3R_n]_S - S_{IM}(k_{-1}^{IP_3R}+l_{-2}^{IP_3R})[IP_3R_{i1}]_S}_{\text{Calcium inactivating resting IP}_3\text{R}} \tag{72}
$$

| Parameter | Value | Units | Reference |
|---|---|---|---|
| $k_1^{\mathrm{IP_3R}}$ | 640000 | 1/M-s | [13] |
| $k_{-1}^{\mathrm{IP_3R}}$ | 0.04 | 1/s | [13] |
| $k_2^{\mathrm{IP_3R}}$ | 37400000 | 1/M-s | [13] |
| $k_{-2}^{\mathrm{IP_3R}}$ | 1.4 | 1/s | [13] |
| $k_3^{\mathrm{IP_3R}}$ | 0.11 | 1/M-s | [13] |
| $k_{-3}^{\mathrm{IP_3R}}$ | 29.8 | 1/s | [13] |
| $k_4^{\mathrm{IP_3R}}$ | 4000000 | 1/M-s | [13] |
| $k_{-4}^{\mathrm{IP_3R}}$ | 0.54 | 1/s | [13] |
| $L_1^{\mathrm{IP_3R}}$ | 1.2e-07 | M | [13] |
| $L_3^{\mathrm{IP_3R}}$ | 2.5e-08 | M | [13] |
| $L_5^{\mathrm{IP_3R}}$ | 5.47e-05 | M | [13] |
| $l_2^{\mathrm{IP_3R}}$ | 1.7 | 1/s | [13] |
| $l_4^{\mathrm{IP_3R}}$ | 1700000 | 1/M-s | [13] |
| $l_6^{\mathrm{IP_3R}}$ | 4707 | 1/s | [13] |
| $l_{-2}^{\mathrm{IP_3R}}$ | 0.8 | 1/s | [13] |
| $l_{-4}^{\mathrm{IP_3R}}$ | 2500000 | 1/M-s | [13] |
| $l_{-6}^{\mathrm{IP_3R}}$ | 11.4 | 1/s | [13] |
| $N^{\mathrm{IP_3R}}$ | 3361 | # | [17] |

Tab. S16: Parameter values associated with $\mathrm{IP_3R}$. At time $t = 0$ s in the steady-state simulation, $[\mathrm{IP_3R_n}]_S = N^{\mathrm{IP_3R}}/S_{\mathrm{IM}}$ and all other states are initialized to 0.

$$S_{\mathrm{IM}}\frac{d}{dt}[\mathrm{IP_3R_o}]_S = \underbrace{S_{\mathrm{IM}}\frac{k_2^{\mathrm{IP_3R}}L_3^{\mathrm{IP_3R}} + l_4^{\mathrm{IP_3R}}[\mathrm{Ca^{2+}_{cyt}}]}{L_3^{\mathrm{IP_3R}} + [\mathrm{Ca^{2+}_{cyt}}](1 + L_3^{\mathrm{IP_3R}}/L_1^{\mathrm{IP_3R}})}[\mathrm{IP_3R_n}]_S[\mathrm{IP_3}] - S_{\mathrm{IM}}\frac{k_{-2}^{\mathrm{IP_3R}} + l_{-4}^{\mathrm{IP_3R}}[\mathrm{Ca^{2+}_{cyt}}]}{1 + [\mathrm{Ca^{2+}_{cyt}}]/L_5^{\mathrm{IP_3R}}}[\mathrm{IP_3R_o}]_S}_{\mathrm{IP_3\ binding\ to\ receptor}}$$
$$\underbrace{-S_{\mathrm{IM}}\frac{(k_4^{\mathrm{IP_3R}}L_5^{\mathrm{IP_3R}} + l_6^{\mathrm{IP_3R}})}{L_5^{\mathrm{IP_3R}} + [\mathrm{Ca^{2+}_{cyt}}]}[\mathrm{IP_3R_o}]_S[\mathrm{Ca^{2+}_{cyt}}] + S_{\mathrm{IM}}\frac{L_1^{\mathrm{IP_3R}}(k_{-4}^{\mathrm{IP_3R}} + l_{-6}^{\mathrm{IP_3R}})}{L_1^{\mathrm{IP_3R}} + [\mathrm{Ca^{2+}_{cyt}}]}[\mathrm{IP_3R_a}]_S}_{\text{Calcium activating receptor}} \tag{73}$$
$$\underbrace{-S_{\mathrm{IM}}\frac{k_3^{\mathrm{IP_3R}}L_5^{\mathrm{IP_3R}}}{L_5^{\mathrm{IP_3R}} + [\mathrm{Ca^{2+}_{cyt}}]}[\mathrm{IP_3R_o}]_S + S_{\mathrm{IM}}k_{-3}^{\mathrm{IP_3R}}[\mathrm{IP_3R_s}]_S}_{\text{receptor closing}}$$

$$S_{\mathrm{IM}}\frac{d}{dt}[\mathrm{IP_3R_a}]_S = \underbrace{S_{\mathrm{IM}}\frac{(k_4^{\mathrm{IP_3R}}L_5^{\mathrm{IP_3R}} + l_6^{\mathrm{IP_3R}})}{L_5^{\mathrm{IP_3R}} + [\mathrm{Ca^{2+}_{cyt}}]}[\mathrm{IP_3R_o}]_S[\mathrm{Ca^{2+}_{cyt}}] - S_{\mathrm{IM}}\frac{L_1^{\mathrm{IP_3R}}(k_{-4}^{\mathrm{IP_3R}} + l_{-6}^{\mathrm{IP_3R}})}{L_1^{\mathrm{IP_3R}} + [\mathrm{Ca^{2+}_{cyt}}]}[\mathrm{IP_3R_a}]_S}_{\text{Calcium activating receptor}}$$
$$\underbrace{-S_{\mathrm{IM}}\frac{k_1^{\mathrm{IP_3R}}L_1^{\mathrm{IP_3R}} + l_2^{\mathrm{IP_3R}}}{L_1^{\mathrm{IP_3R}} + [\mathrm{Ca^{2+}_{cyt}}]}[\mathrm{IP_3R_a}]_S[\mathrm{Ca^{2+}_{cyt}}] + S_{\mathrm{IM}}(k_{-1}^{\mathrm{IP_3R}} + l_{-2}^{\mathrm{IP_3R}})[\mathrm{IP_3R_{i2}}]_S}_{\text{Calcium inactivating active }\mathrm{IP_3R}} \tag{74}$$

$$S_{\mathrm{IM}}\frac{d}{dt}[\mathrm{IP_3R_s}]_S = \underbrace{-S_{\mathrm{IM}}\frac{k_3^{\mathrm{IP_3R}}L_5^{\mathrm{IP_3R}}}{L_5^{\mathrm{IP_3R}} + [\mathrm{Ca^{2+}_{cyt}}]}[\mathrm{IP_3R_o}]_S + S_{\mathrm{IM}}k_{-3}^{\mathrm{IP_3R}}[\mathrm{IP_3R_s}]_S}_{\text{receptor closing}} \tag{75}$$

$$S_{\mathrm{IM}}\frac{d}{dt}[\mathrm{IP_3R_{i2}}]_S = \underbrace{S_{\mathrm{IM}}\frac{k_1^{\mathrm{IP_3R}}L_1^{\mathrm{IP_3R}} + l_2^{\mathrm{IP_3R}}}{L_1^{\mathrm{IP_3R}} + [\mathrm{Ca^{2+}_{cyt}}]}[\mathrm{IP_3R_a}]_S[\mathrm{Ca^{2+}_{cyt}}] - S_{\mathrm{IM}}(k_{-1}^{\mathrm{IP_3R}} + l_{-2}^{\mathrm{IP_3R}})[\mathrm{IP_3R_{i2}}]_S}_{\text{Calcium inactivating active }\mathrm{IP_3R}} \tag{76}$$

### S4.7.2 SERCA

The full SERCA pump model is a 6 state model that involves shuttling two calcium ions across the DTS membrane at a time. The three states without a calcium bound are assumed to be in a quasi-steady state equilibrium with each other. Let $[\text{SERCA}]_S = [\text{SERCA}_{\text{E1}}]_S + [\text{SERCA}_{\text{E2}}]_S + [\text{SERCA}_{\text{E2}}\text{P}]_S$. Then the ratios as follows:

$$[\text{SERCA}_{\text{E1}}]_S = (0.24996)[\text{SERCA}]_S$$
$$[\text{SERCA}_{\text{E2}}]_S = (0.74989)[\text{SERCA}]_S$$
$$[\text{SERCA}_{\text{E2}}\text{P}]_S = (1.4998 \cdot 10^{-4})[\text{SERCA}]_S$$

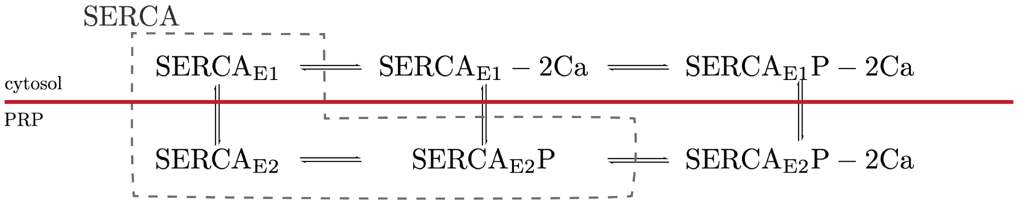


Fig. S5: SERCA reaction diagram. The three states without a calcium bound are all assumed to be in equilibrium with each other.

| Parameter | Value | Units | Reference |
|---|---|---|---|
| $k_{2f}^{\text{SERCA}}$ | See Table S1 | $1/\text{M}^2$-s | |
| $k_{2r}^{\text{SERCA}}$ | See Table S1 | 1/s | |
| $k_{3f}^{\text{SERCA}}$ | See Table S1 | 1/s | |
| $k_{3r}^{\text{SERCA}}$ | See Table S1 | 1/s | |
| $k_{4f}^{\text{SERCA}}$ | See Table S1 | 1/s | |
| $k_{4r}^{\text{SERCA}}$ | See Table S1 | 1/s | |
| $k_{5f}^{\text{SERCA}}$ | See Table S1 | 1/s | |
| $k_{5r}^{\text{SERCA}}$ | See Table S1 | $1/\text{M}^2$-s | |
| $N^{\text{SERCA}}$ | 57083 | # | [17] |

Tab. S17: Parameter values associated with SERCA. At time $t = 0$ s in the steady-state simulation, $[\text{SERCA}]_S = N^{\text{SERCA}}/S_{\text{IM}}$ and all other states are initialized to 0.

$$S_{\rm IM}\frac{d}{dt}[\text{SERCA}]_S = \underbrace{-S_{\rm IM}k_{\rm 2f}^{\rm SERCA}[\text{SERCA}_{\rm E1}]_S[\text{Ca}_{\rm cyt}^{2+}]^2 + S_{\rm IM}k_{\rm 2r}^{\rm SERCA}[\text{SERCA}_{\rm E1}-2\text{Ca}]_S}_{\text{2Ca}_{\rm cyt}\text{ ions binding to SERCA}} \underbrace{+S_{\rm IM}k_{\rm 5f}^{\rm SERCA}[\text{SERCA}_{\rm E2}\text{P}-2\text{Ca}]_S - S_{\rm IM}k_{\rm 5r}^{\rm SERCA}[\text{SERCA}_{\rm E2}\text{P}]_S[\text{Ca}_{\rm DTS}^{2+}]^2}_{\text{2Ca}_{\rm dts}\text{ ions binding to SERCA}} \tag{77}$$

$$S_{\rm IM}\frac{d}{dt}[\text{SERCA}_{\rm E1}-2\text{Ca}]_S = \underbrace{S_{\rm IM}k_{\rm 2f}^{\rm SERCA}[\text{SERCA}_{\rm E1}]_S[\text{Ca}_{\rm cyt}^{2+}]^2 - S_{\rm IM}k_{\rm 2r}^{\rm SERCA}[\text{SERCA}_{\rm E1}-2\text{Ca}]_S}_{\text{2Ca}_{\rm cyt}\text{ ions binding to SERCA}} \underbrace{-S_{\rm IM}k_{\rm 3f}^{\rm SERCA}[\text{SERCA}_{\rm E1}-\text{Ca}_2]_S + S_{\rm IM}k_{\rm 3r}^{\rm SERCA}[\text{SERCA}_{\rm E1}\text{P}-2\text{Ca}]_S}_{\text{SERCA phosphorylation}} \tag{78}$$

$$S_{\rm IM}\frac{d}{dt}[\text{SERCA}_{\rm E1}\text{P}-2\text{Ca}]_S = \underbrace{S_{\rm IM}k_{\rm 3f}^{\rm SERCA}[\text{SERCA}_{\rm E1}-2\text{Ca}]_S + S_{\rm IM}k_{\rm 3r}^{\rm SERCA}[\text{SERCA}_{\rm E1}\text{P}-2\text{Ca}]_S}_{\text{SERCA phosphorylation}} \underbrace{-S_{\rm IM}k_{\rm 4f}^{\rm SERCA}[\text{SERCA}_{\rm E1}\text{P}-2\text{Ca}]_S + S_{\rm IM}k_{\rm 4r}^{\rm SERCA}[\text{SERCA}_{\rm E2}\text{P}-2\text{Ca}]_S}_{\text{SERCA shuttling into DTS}} \tag{79}$$

$$S_{\rm IM}\frac{d}{dt}[\text{SERCA}_{\rm E2}\text{P}-2\text{Ca}]_S = \underbrace{S_{\rm IM}k_{\rm 4f}^{\rm SERCA}[\text{SERCA}_{\rm E1}\text{P}-2\text{Ca}]_S - S_{\rm IM}k_{\rm 4r}^{\rm SERCA}[\text{SERCA}_{\rm E2}\text{P}-2\text{Ca}]_S}_{\text{SERCA shuttling into DTS}} \underbrace{-S_{\rm IM}k_{\rm 5f}^{\rm SERCA}[\text{SERCA}_{\rm E2}\text{P}-2\text{Ca}]_S + S_{\rm IM}k_{\rm 5r}^{\rm SERCA}[\text{SERCA}_{\rm E2}\text{P}]_S[\text{Ca}_{\rm DTS}^{2+}]^2}_{\text{2Ca}_{\rm dts}\text{ ions binding to SERCA}} \tag{80}$$

### S4.7.3 PMCA

The full PMCA pump model is a 6 state model that involves shuttling two calcium ions across the plasma membrane at a time. The three states without a calcium bound are assumed to be in a quasi-steady state equilibrium with each other. Let $[\text{PMCA}]_S = [\text{PMCA}_{\text{E1}}]_S + [\text{PMCA}_{\text{E2}}]_S + [\text{PMCA}_{\text{E2}}\text{P}]_S$. Then the ratios as follows:

$$[\text{PMCA}_{\text{E1}}]_S = (0.24996)[\text{PMCA}]_S$$
$$[\text{PMCA}_{\text{E2}}]_S = (0.74989)[\text{PMCA}]_S$$
$$[\text{PMCA}_{\text{E2}}\text{P}]_S = (1.4998 \cdot 10^{-4})[\text{PMCA}]_S$$

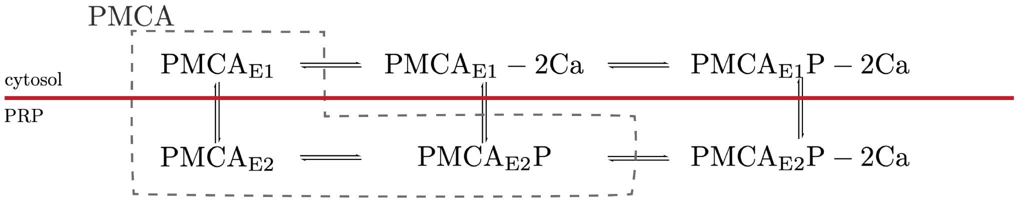


Fig. S6: PMCA reaction diagram. The three states without a calcium bound are all assumed to be in equilibrium with each other.

| Parameter | Value | Units | Reference |
|---|---|---|---|
| $k_{2f}^{\text{PMCA}}$ | See Table S1 | 1/M$^2$-s | |
| $k_{2r}^{\text{PMCA}}$ | See Table S1 | 1/s | |
| $k_{3f}^{\text{PMCA}}$ | See Table S1 | 1/s | |
| $k_{3r}^{\text{PMCA}}$ | See Table S1 | 1/s | |
| $k_{4f}^{\text{PMCA}}$ | See Table S1 | 1/s | |
| $k_{4r}^{\text{PMCA}}$ | See Table S1 | 1/s | |
| $k_{5f}^{\text{PMCA}}$ | See Table S1 | 1/s | |
| $k_{5r}^{\text{PMCA}}$ | See Table S1 | 1/M$^2$-s | |
| $N^{\text{PMCA}}$ | 611 | # | [17] |

Tab. S18: Parameter values associated with PMCA. At time $t = 0$ s in the steady-state simulation, $[\text{PMCA}]_S = N^{\text{PMCA}}/S_{\text{PM}}$ and all other states are initialized to 0.

$$S_{\mathrm{PM}}\frac{d}{dt}[\mathrm{PMCA}]_S = \underbrace{-S_{\mathrm{PM}}k_{2\mathrm{f}}^{\mathrm{PMCA}}[\mathrm{PMCA}_{\mathrm{E1}}]_S[\mathrm{Ca}_{\mathrm{cyt}}^{2+}]^2 + S_{\mathrm{PM}}k_{2\mathrm{r}}^{\mathrm{PMCA}}[\mathrm{PMCA}_{\mathrm{E1}} - 2\mathrm{Ca}]_S}_{2\mathrm{Ca}_{\mathrm{cyt}}\text{ ions binding to PMCA}} \underbrace{+S_{\mathrm{PM}}k_{5\mathrm{f}}^{\mathrm{PMCA}}[\mathrm{PMCA}_{\mathrm{E2}}\mathrm{P} - 2\mathrm{Ca}]_S - S_{\mathrm{PM}}k_{5\mathrm{r}}^{\mathrm{PMCA}}[\mathrm{PMCA}_{\mathrm{E2}}\mathrm{P}]_S[\mathrm{Ca}_{\mathrm{PRP}}^{2+}]^2}_{2\mathrm{Ca}_{\mathrm{dts}}\text{ ions binding to PMCA}} \tag{81}$$

$$S_{\mathrm{PM}}\frac{d}{dt}[\mathrm{PMCA}_{\mathrm{E1}} - 2\mathrm{Ca}]_S = \underbrace{S_{\mathrm{PM}}k_{2\mathrm{f}}^{\mathrm{PMCA}}[\mathrm{PMCA}_{\mathrm{E1}}]_S[\mathrm{Ca}_{\mathrm{cyt}}^{2+}]^2 - S_{\mathrm{PM}}k_{2\mathrm{r}}^{\mathrm{PMCA}}[\mathrm{PMCA}_{\mathrm{E1}} - 2\mathrm{Ca}]_S}_{2\mathrm{Ca}_{\mathrm{cyt}}\text{ ions binding to PMCA}} \underbrace{-S_{\mathrm{PM}}k_{3\mathrm{f}}^{\mathrm{PMCA}}[\mathrm{PMCA}_{\mathrm{E1}} - 2\mathrm{Ca}]_S + S_{\mathrm{PM}}k_{3\mathrm{r}}^{\mathrm{PMCA}}[\mathrm{PMCA}_{\mathrm{E1}}\mathrm{P} - 2\mathrm{Ca}]_S}_{\text{PMCA phosphorylation}} \tag{82}$$

$$S_{\mathrm{PM}}\frac{d}{dt}[\mathrm{PMCA}_{\mathrm{E1}}\mathrm{P} - 2\mathrm{Ca}]_S = \underbrace{S_{\mathrm{PM}}k_{3\mathrm{f}}^{\mathrm{PMCA}}[\mathrm{PMCA}_{\mathrm{E1}} - 2\mathrm{Ca}]_S + S_{\mathrm{PM}}k_{3\mathrm{r}}^{\mathrm{PMCA}}[\mathrm{PMCA}_{\mathrm{E1}}\mathrm{P} - 2\mathrm{Ca}]_S}_{\text{PMCA phosphorylation}} \underbrace{-S_{\mathrm{PM}}k_{4\mathrm{f}}^{\mathrm{PMCA}}[\mathrm{PMCA}_{\mathrm{E1}}\mathrm{P} - 2\mathrm{Ca}]_S + S_{\mathrm{PM}}k_{4\mathrm{r}}^{\mathrm{PMCA}}[\mathrm{PMCA}_{\mathrm{E2}}\mathrm{P} - 2\mathrm{Ca}]_S}_{\text{PMCA shuttling into PRP}} \tag{83}$$

$$S_{\mathrm{PM}}\frac{d}{dt}[\mathrm{PMCA}_{\mathrm{E2}}\mathrm{P} - 2\mathrm{Ca}]_S = \underbrace{S_{\mathrm{PM}}k_{4\mathrm{f}}^{\mathrm{PMCA}}[\mathrm{PMCA}_{\mathrm{E1}}\mathrm{P} - 2\mathrm{Ca}]_S - S_{\mathrm{PM}}k_{4\mathrm{r}}^{\mathrm{PMCA}}[\mathrm{PMCA}_{\mathrm{E2}}\mathrm{P} - 2\mathrm{Ca}]_S}_{\text{PMCA shuttling into PRP}} \underbrace{-S_{\mathrm{PM}}k_{5\mathrm{f}}^{\mathrm{PMCA}}[\mathrm{PMCA}_{\mathrm{E2}}\mathrm{P} - 2\mathrm{Ca}]_S + S_{\mathrm{PM}}k_{5\mathrm{r}}^{\mathrm{PMCA}}[\mathrm{PMCA}_{\mathrm{E2}}\mathrm{P}]_S[\mathrm{Ca}_{\mathrm{PRP}}^{2+}]^2}_{2\mathrm{Ca}_{\mathrm{dts}}\text{ ions binding to PMCA}} \tag{84}$$

# S5 Quasi Steady State Approximation

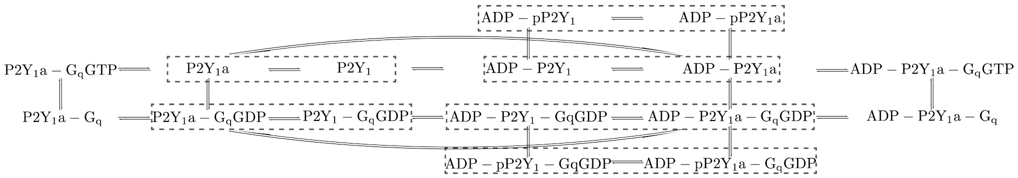


Fig. S7: $\mathrm{P2Y_1}$ reaction diagram without a quasi steady state approximation. Four states are added on top of the model used in Purvis et al. [12], $\mathrm{p-P2Y_1-G_qGDP}$, $\mathrm{ADP-pP2Y_1-G_qGDP}$, $\mathrm{p-P2Y_1a-G_qGDP}$, and $\mathrm{ADP-pP2Y_1a-G_qGDP}$ These states correspond to a desensitized receptor mediated by PKC.

We briefly outline the process of reducing the number of equations in Purvis et al. [12] by utilizing a quasi steady state (QSS) assumption. The work here is for the $\mathrm{P2Y_1}$ receptor only; the exact same computation can be done to the $\mathrm{P2Y_{12}}$ receptor to yield analogous equations. Figure S7 shows the entire reaction diagram for the $\mathrm{P2Y_1}$ receptor. It is generally assumed for G-protein coupled receptors that the receptors must be in an "active" conformation in order to process G protein [7]. Our assumption is that the receptor in the "active" conformation is in a quasi-steady state equilibrium with the corresponding "inactive" conformation, showed by the grey boxes in the figure. Under this assumption (to be made more precise below), we can reduce the number of equations by six

Below, we write out each of the 12 equations that are contained in the grey boxes in Figure S7. For concision, we divide all terms by $S_{\mathrm{PM}}$. The full equations are as follows:

$$\frac{d}{dt}[\mathrm{P2Y_1}]_S = \underbrace{-k_{\mathrm{act}}^{\mathrm{P2Y_1}}[\mathrm{P2Y_1}]_S + k_{\mathrm{-act}}^{\mathrm{P2Y_1}}[\mathrm{P2Y_1a}]_S}_{\text{activation}} - \underbrace{k_{\mathrm{ADP}}^{\mathrm{P2Y_1}}[\mathrm{P2Y_1}]_S[\mathrm{ADP}] + k_{\mathrm{-ADP}}^{\mathrm{P2Y_1}}[\mathrm{ADP-P2Y_1}]_S}_{\text{ADP Binding}} \tag{85}$$

$$\begin{aligned}\frac{d}{dt}[\mathrm{P2Y_1a}]_S = &\underbrace{k_{\mathrm{act}}^{\mathrm{P2Y_1}}[\mathrm{P2Y_1}]_S - k_{\mathrm{-act}}^{\mathrm{P2Y_1}}[\mathrm{P2Y_1a}]_S}_{\text{activation}} - \underbrace{\alpha k_{\mathrm{ADP}}^{\mathrm{P2Y_1}}[\mathrm{P2Y_1a}]_S[\mathrm{ADP}] + k_{\mathrm{-ADP}}^{\mathrm{P2Y_1}}[\mathrm{ADP-P2Y_1a}]_S}_{\text{ADP Binding}} \\ &\underbrace{-\beta k_{\mathrm{G_qGDP}}^{\mathrm{P2Y_1}}[\mathrm{P2Y_1a}]_S[\mathrm{G_qGDP}]_S + k_{\mathrm{-G_qGDP}}^{\mathrm{P2Y_1}}[\mathrm{P2Y_1a-G_qGDP}]_S}_{\mathrm{G}_q\text{GDP binding}} \\ &\underbrace{-k_{\mathrm{G_qGTP}}^{\mathrm{P2Y_1}}[\mathrm{P2Y_1a}]_S[\mathrm{G_qGTP}]_S + k_{\mathrm{-G_qGTP}}^{\mathrm{P2Y_1}}[\mathrm{P2Y_1a-G_qGTP}]_S}_{\mathrm{G}_q\text{GTP binding}}\end{aligned} \tag{86}$$

$$\begin{aligned}\frac{d}{dt}[\mathrm{ADP-P2Y_1}]_S = &\underbrace{-\alpha k_{\mathrm{act}}^{\mathrm{P2Y_1}}[\mathrm{ADP-P2Y_1}]_S + k_{\mathrm{-act}}^{\mathrm{P2Y_1}}[\mathrm{ADP-P2Y_1a}]_S}_{\text{activation}} + \underbrace{k_{\mathrm{ADP}}^{\mathrm{P2Y_1}}[\mathrm{P2Y_1}]_S[\mathrm{ADP}] - k_{\mathrm{-ADP}}^{\mathrm{P2Y_1}}[\mathrm{ADP-P2Y_1}]_S}_{\text{ADP Binding}} \\ &\underbrace{-k_{\mathrm{phos}}^{\mathrm{P2Y_1}}[\mathrm{ADP-P2Y_1}]_S[\mathrm{PKCa-Ca-DAG}] + k_{\mathrm{dephos}}^{\mathrm{P2Y_1}}[\mathrm{ADP-pP2Y_1}]_S}_{\text{Desensitization by PKC}}\end{aligned} \tag{87}$$

$$\begin{aligned}\frac{d}{dt}[\mathrm{ADP-P2Y_1a}]_S = &\underbrace{\alpha k_{\mathrm{act}}^{\mathrm{P2Y_1}}[\mathrm{ADP-P2Y_1}]_S - k_{\mathrm{-act}}^{\mathrm{P2Y_1}}[\mathrm{ADP-P2Y_1a}]_S}_{\text{activation}} + \underbrace{\alpha k_{\mathrm{ADP}}^{\mathrm{P2Y_1}}[\mathrm{P2Y_1a}]_S[\mathrm{ADP}] - k_{\mathrm{-ADP}}^{\mathrm{P2Y_1}}[\mathrm{ADP-P2Y_1a}]_S}_{\text{ADP Binding}} \\ &\underbrace{-k_{\mathrm{phos}}^{\mathrm{P2Y_1}}[\mathrm{ADP-P2Y_1a}]_S[\mathrm{PKCa-Ca-DAG}] + k_{\mathrm{dephos}}^{\mathrm{P2Y_1}}[\mathrm{ADP-pP2Y_1a}]_S}_{\text{Desensitization by PKC}} \\ &- \underbrace{\beta k_{\mathrm{G_qGDP}}^{\mathrm{P2Y_1}}[\mathrm{ADP-P2Y_1a}]_S[\mathrm{G_qGDP}]_S + \frac{k_{\mathrm{-G_qGDP}}^{\mathrm{P2Y_1}}}{\delta\gamma}[\mathrm{ADP-P2Y_1a-G_qGDP}]_S}_{\mathrm{G}_q\text{GDP binding}} \\ &- \underbrace{k_{\mathrm{G_qGTP}}^{\mathrm{P2Y_1}}[\mathrm{ADP-P2Y_1a}]_S[\mathrm{G_qGTP}]_S + k_{\mathrm{-G_qGTP}}^{\mathrm{P2Y_1}}[\mathrm{ADP-P2Y_1a-G_qGTP}]_S}_{\mathrm{G}_q\text{GTP binding}}\end{aligned} \tag{88}$$

$$\frac{d}{dt}[\mathrm{P2Y_1-G_qGDP}]_S = \underbrace{-\beta k_{\mathrm{act}}^{\mathrm{P2Y_1}}[\mathrm{P2Y_1-G_qGDP}]_S + k_{\mathrm{-act}}^{\mathrm{P2Y_1}}[\mathrm{P2Y_1a-G_qGDP}]_S}_{\text{activation}} \underbrace{- k_{\mathrm{ADP}}^{\mathrm{P2Y_1}}[\mathrm{P2Y_1-G_qGDP}]_S[\mathrm{ADP}] + \frac{k_{\mathrm{-ADP}}^{\mathrm{P2Y_1}}}{\gamma}[\mathrm{ADP-P2Y_1-G_qGDP}]_S}_{\text{ADP Binding}} \tag{89}$$

$$\begin{aligned}\frac{d}{dt}[\mathrm{P2Y_1a-G_qGDP}]_S = &\underbrace{\beta k_{\mathrm{act}}^{\mathrm{P2Y_1}}[\mathrm{P2Y_1-G_qGDP}]_S - k_{\mathrm{-act}}^{\mathrm{P2Y_1}}[\mathrm{P2Y_1a-G_qGDP}]_S}_{\text{activation}} \\ &\underbrace{- \alpha k_{\mathrm{ADP}}^{\mathrm{P2Y_1}}[\mathrm{P2Y_1a-G_qGDP}]_S[\mathrm{ADP}] + \frac{k_{\mathrm{-ADP}}^{\mathrm{P2Y_1}}}{\delta\gamma}[\mathrm{ADP-P2Y_1a-G_qGDP}]_S}_{\text{ADP Binding}} \\ &\underbrace{+ \beta k_{\mathrm{G_qGDP}}^{\mathrm{P2Y_1}}[\mathrm{P2Y_1a}]_S[\mathrm{G_qGDP}]_S - k_{\mathrm{-G_qGDP}}^{\mathrm{P2Y_1}}[\mathrm{P2Y_1a-G_qGDP}]_S}_{\mathrm{G}_q\text{GDP binding}} \\ &\underbrace{-k_{\mathrm{GDP}}^{\mathrm{P2Y_1}}[\mathrm{P2Y_1a-G_qGDP}]_S + k_{\mathrm{-GDP}}^{\mathrm{P2Y_1}}[\mathrm{P2Y_1a-G_q}]_S[\mathrm{GDP}]}_{\text{GDP binding}}\end{aligned} \tag{90}$$

$$\begin{aligned}\frac{d}{dt}[\mathrm{ADP-P2Y_1-G_qGDP}]_S = &\underbrace{- \alpha\beta\delta k_{\mathrm{act}}^{\mathrm{P2Y_1}}[\mathrm{ADP-P2Y_1-G_qGDP}]_S + k_{\mathrm{-act}}^{\mathrm{P2Y_1}}[\mathrm{ADP-P2Y_1a-G_qGDP}]_S}_{\text{activation}} \\ &\underbrace{+ k_{\mathrm{ADP}}^{\mathrm{P2Y_1}}[\mathrm{P2Y_1-G_qGDP}]_S[\mathrm{ADP}] - \frac{k_{\mathrm{-ADP}}^{\mathrm{P2Y_1}}}{\gamma}[\mathrm{ADP-P2Y_1-G_qGDP}]_S}_{\text{ADP Binding}} \\ &\underbrace{-k_{\mathrm{phos}}^{\mathrm{P2Y_1}}[\mathrm{ADP-P2Y_1-G_qGDP}]_S[\mathrm{PKCa-Ca-DAG}] + k_{\mathrm{dephos}}^{\mathrm{P2Y_1}}[\mathrm{ADP-pP2Y_1-G_qGDP}]_S}_{\text{Desensitization by PKC}}\end{aligned} \tag{91}$$

$$\begin{aligned}\frac{d}{dt}[\mathrm{ADP-P2Y_1a-G_qGDP}]_S = &\underbrace{\alpha\beta\delta k_{\mathrm{act}}^{\mathrm{P2Y_1}}[\mathrm{ADP-P2Y_1-G_qGDP}]_S - k_{\mathrm{-act}}^{\mathrm{P2Y_1}}[\mathrm{ADP-P2Y_1a-G_qGDP}]_S}_{\text{activation}} \\ &\underbrace{+ \alpha k_{\mathrm{ADP}}^{\mathrm{P2Y_1}}[\mathrm{P2Y_1a-G_qGDP}]_S[\mathrm{ADP}] - \frac{k_{\mathrm{-ADP}}^{\mathrm{P2Y_1}}}{\delta\gamma}[\mathrm{ADP-P2Y_1a-G_qGDP}]_S}_{\text{ADP Binding}} \\ &\underbrace{-k_{\mathrm{phos}}^{\mathrm{P2Y_1}}[\mathrm{ADP-P2Y_1a-G_qGDP}]_S[\mathrm{PKCa-Ca-DAG}] + k_{\mathrm{dephos}}^{\mathrm{P2Y_1}}[\mathrm{ADP-pP2Y_1a-G_qGDP}]_S}_{\text{Desensitization by PKC}} \\ &\underbrace{+\beta k_{\mathrm{G_qGDP}}^{\mathrm{P2Y_1}}[\mathrm{ADP-P2Y_1a}]_S[\mathrm{G_qGDP}]_S - \frac{k_{\mathrm{-G_qGDP}}^{\mathrm{P2Y_1}}}{\delta\gamma}[\mathrm{ADP-P2Y_1a-G_qGDP}]_S}_{\mathrm{G}_q\text{GDP binding}} \\ &\underbrace{-k_{\mathrm{G_qGDP}}^{\mathrm{P2Y_1}}[\mathrm{ADP-P2Y_1a-G_qGDP}]_S + k_{\mathrm{-GDP}}^{\mathrm{P2Y_1}}[\mathrm{ADP-P2Y_1-G_q}]_S[\mathrm{GDP}]}_{\text{GDP binding}}\end{aligned} \tag{92}$$

$$\frac{d}{dt}[\mathrm{ADP-pP2Y_1}]_S = \underbrace{-\alpha k_{\mathrm{act}}^{\mathrm{P2Y_1}}[\mathrm{ADP-pP2Y_1}]_S + k_{\mathrm{-act}}^{\mathrm{P2Y_1}}[\mathrm{ADP-pP2Y_1a}]_S}_{\text{activation}} \underbrace{-k_{\mathrm{phos}}^{\mathrm{P2Y_1}}[\mathrm{ADP-P2Y_1}]_S[\mathrm{PKCa-Ca-DAG}] + k_{\mathrm{dephos}}^{\mathrm{P2Y_1}}[\mathrm{ADP-pP2Y_1}]_S}_{\text{Desensitization by PKC}} \tag{93}$$

$$\frac{d}{dt}[\mathrm{ADP-pP2Y_1a}]_S = \underbrace{\alpha k_{\mathrm{act}}^{\mathrm{P2Y_1}}[\mathrm{ADP-pP2Y_1}]_S - k_{\mathrm{-act}}^{\mathrm{P2Y_1}}[\mathrm{ADP-pP2Y_1a}]_S}_{\text{activation}} \underbrace{-k_{\mathrm{phos}}^{\mathrm{P2Y_1}}[\mathrm{ADP-P2Y_1a}]_S[\mathrm{PKCa-Ca-DAG}] + k_{\mathrm{dephos}}^{\mathrm{P2Y_1}}[\mathrm{ADP-pP2Y_1a}]_S}_{\text{Desensitization by PKC}} \tag{94}$$

$$\frac{d}{dt}[\mathrm{ADP-pP2Y_1-G_qGDP}]_S = \underbrace{-\alpha\beta\delta k_{\mathrm{act}}^{\mathrm{P2Y_1}}[\mathrm{ADP-pP2Y_1-G_qGDP}]_S + k_{\mathrm{-act}}^{\mathrm{P2Y_1}}[\mathrm{ADP-pP2Y_1a-G_qGDP}]_S}_{\text{activation}} \underbrace{+k_{\mathrm{phos}}^{\mathrm{P2Y_1}}[\mathrm{ADP-P2Y_1-G_qGDP}]_S[\mathrm{PKCa-Ca-DAG}] - k_{\mathrm{dephos}}^{\mathrm{P2Y_1}}[\mathrm{ADP-pP2Y_1-G_qGDP}]_S}_{\text{Desensitization by PKC}} \tag{95}$$

$$\frac{d}{dt}[\mathrm{ADP-pP2Y_1a-G_qGDP}]_S = \underbrace{\alpha\beta\delta k_{\mathrm{act}}^{\mathrm{P2Y_1}}[\mathrm{ADP-pP2Y_1-G_qGDP}]_S - k_{\mathrm{-act}}^{\mathrm{P2Y_1}}[\mathrm{ADP-pP2Y_1a-G_qGDP}]_S}_{\text{activation}} \underbrace{+k_{\mathrm{phos}}^{\mathrm{P2Y_1}}[\mathrm{ADP-P2Y_1a-G_qGDP}]_S[\mathrm{PKCa-Ca-DAG}] - k_{\mathrm{dephos}}^{\mathrm{P2Y_1}}[\mathrm{ADP-pP2Y_1a-G_qGDP}]_S}_{\text{Desensitization by PKC}} \tag{96}$$

If we make the assumption that $k_{\mathrm{act}}^{\mathrm{P2Y_1}}$ is large compared to other reaction rates (i.e. $k_{\mathrm{-ADP}}^{\mathrm{P2Y_1}}$ and $k_{\mathrm{-G_qGDP}}^{\mathrm{P2Y_1}}$) under the condition that $k_{\mathrm{-act}}^{\mathrm{P2Y_1}}/k_{\mathrm{act}}^{\mathrm{P2Y_1}} = K_{\mathrm{act}}^{\mathrm{P2Y_1}}$ is constant, then Equations 85, 93, 89, and 95 reduce to the following algebraic relationships:

$$[\mathrm{P2Y_1a}]_S = K_{\mathrm{act}}[\mathrm{P2Y_1}]_S \tag{97a}$$
$$[\mathrm{ADP-P2Y_1a}]_S = \alpha K_{\mathrm{act}}[\mathrm{ADP-P2Y_1}]_S \tag{97b}$$
$$[\mathrm{P2Y_1a-G_qGDP}]_S = \beta K_{\mathrm{act}}[\mathrm{P2Y_1-G_qGDP}]_S \tag{97c}$$
$$[\mathrm{ADP-P2Y_1a-G_qGDP}]_S = \alpha\beta\delta K_{\mathrm{act}}[\mathrm{ADP-P2Y_1-G_qGDP}]_S \tag{97d}$$
$$[\mathrm{ADP-pP2Y_1a}]_S = \alpha K_{\mathrm{act}}[\mathrm{ADP-pP2Y_1}]_S \tag{97e}$$
$$[\mathrm{ADP-pP2Y_1a-G_qGDP}]_S = \alpha\beta\delta K_{\mathrm{act}}[\mathrm{ADP-pP2Y_1-G_qGDP}]_S \tag{97f}$$

We then define the concentrations $\overline{[\mathrm{P2Y_1}]}_S = [\mathrm{P2Y_1a}]_S + [\mathrm{P2Y_1}]_S$, and similar for the other three equations above. Plugging in the above equations into these definitions yield the following relations:

$$[\mathrm{P2Y_1a}]_S = \frac{K_{\mathrm{act}}}{K_{\mathrm{act}}+1}\overline{[\mathrm{P2Y_1}]}_S \tag{98a}$$

$$[\mathrm{P2Y_1}]_S = \frac{1}{K_{\mathrm{act}}+1}\overline{[\mathrm{P2Y_1}]}_S \tag{98b}$$

$$[\mathrm{ADP-P2Y_1a}]_S = \frac{\alpha K_{\mathrm{act}}}{\alpha K_{\mathrm{act}}+1}\overline{[\mathrm{ADP-P2Y_1}]}_S \tag{98c}$$

$$[\mathrm{ADP-P2Y_1}]_S = \frac{1}{\alpha K_{\mathrm{act}}+1}\overline{[\mathrm{ADP-P2Y_1}]}_S \tag{98d}$$

$$[\mathrm{P2Y_1a-GDP}]_S = \frac{\beta K_{\mathrm{act}}}{\beta K_{\mathrm{act}}+1}\overline{[\mathrm{P2Y_1-GDP}]}_S \tag{98e}$$

$$[\mathrm{P2Y_1-GDP}]_S = \frac{1}{\beta K_{\mathrm{act}}+1}\overline{[\mathrm{P2Y_1-GDP}]}_S \tag{98f}$$

$$[\mathrm{ADP-P2Y_1a-G_qGDP}]_S = \frac{\alpha\beta\delta K_{\mathrm{act}}}{\alpha\beta\delta K_{\mathrm{act}}+1}\overline{[\mathrm{P2Y_1-G_qGDP}]}_S \tag{98g}$$

$$[\mathrm{ADP-P2Y_1-G_qGDP}]_S = \frac{1}{\alpha\beta\delta K_{\mathrm{act}}+1}\overline{[\mathrm{P2Y_1-G_qGDP}]}_S \tag{98h}$$

$$[\mathrm{ADP-pP2Y_1a}]_S = \frac{\alpha K_{\mathrm{act}}}{\alpha K_{\mathrm{act}}+1}\overline{[\mathrm{ADP-pP2Y_1}]}_S \tag{98i}$$

$$[\mathrm{ADP-pP2Y_1}]_S = \frac{1}{\alpha K_{\mathrm{act}}+1}\overline{[\mathrm{ADP-pP2Y_1}]}_S \tag{98j}$$

$$[\mathrm{ADP-pP2Y_1a-G_qGDP}]_S = \frac{\alpha\beta\delta K_{\mathrm{act}}}{\alpha\beta\delta K_{\mathrm{act}}+1}\overline{[\mathrm{pP2Y_1-G_qGDP}]}_S \tag{98k}$$

$$[\mathrm{ADP-pP2Y_1-G_qGDP}]_S = \frac{1}{\alpha\beta\delta K_{\mathrm{act}}+1}\overline{[\mathrm{pP2Y_1-G_qGDP}]}_S \tag{98l}$$

Finally, we compute $\frac{d}{dt}\overline{[\mathrm{P2Y_1}]}_S$ by adding the derivatives of $[\mathrm{P2Y_1a}]_S$ and $[\mathrm{P2Y_1}]_S$ and substituting for $\overline{[\mathrm{P2Y_1}]}_S$ (and equivalently for the other reduced states):

$$\begin{aligned}\frac{d}{dt}\overline{[\mathrm{P2Y_1}]}_S = &-\left(k^{\mathrm{P2Y_1}}_{\mathrm{ADP}}\frac{1}{K_{\mathrm{act}}+1}+k^{\mathrm{P2Y_1}}_{\mathrm{ADP}}\frac{\alpha K_{\mathrm{act}}}{K_{\mathrm{act}}+1}\right)[\mathrm{ADP}]\overline{[\mathrm{P2Y_1}]}_S\\
&+\left(k^{\mathrm{P2Y_1}}_{-\mathrm{ADP}}\frac{1}{\alpha K_{\mathrm{act}}+1}+k^{\mathrm{P2Y_1}}_{-\mathrm{ADP}}\frac{\alpha K_{\mathrm{act}}}{\alpha K_{\mathrm{act}}+1}\right)\overline{[\mathrm{ADP}-\mathrm{P2Y_1}]}_S\\
&-\left(\beta k^{\mathrm{P2Y_1}}_{\mathrm{G_qGDP}}\frac{1}{K_{\mathrm{act}}+1}\right)[\mathrm{G_qGDP}]\overline{[\mathrm{P2Y_1}]}_S\\
&+\left(k^{\mathrm{P2Y_1}}_{-\mathrm{G_qGDP}}\frac{\beta K_{\mathrm{act}}}{\beta K_{\mathrm{act}}+1}\right)\overline{[\mathrm{P2Y_1}-\mathrm{G_qGDP}]}_S\\
&-\left(k^{\mathrm{P2Y_1}}_{\mathrm{G_qGTP}}\frac{K_{\mathrm{act}}}{K_{\mathrm{act}}+1}\right)[\mathrm{G_qGTP}]\overline{[\mathrm{P2Y_1}]}_S\\
&+k^{\mathrm{P2Y_1}}_{-\mathrm{G_qGTP}}[\mathrm{P2Y_1a}-\mathrm{G_qGTP}]_S\end{aligned} \tag{99}$$

$$\begin{aligned}\frac{d}{dt}\overline{[\mathrm{ADP}-\mathrm{P2Y_1}]}_S = &\left(k^{\mathrm{P2Y_1}}_{\mathrm{ADP}}\frac{1}{K_{\mathrm{act}}+1}+\alpha k^{\mathrm{P2Y_1}}_{\mathrm{ADP}}\frac{K_{\mathrm{act}}}{K_{\mathrm{act}}+1}\right)[\mathrm{ADP}]\overline{[\mathrm{P2Y_1}]}_S\\
&-\left(k^{\mathrm{P2Y_1}}_{-\mathrm{ADP}}\frac{1}{\alpha K_{act}+1}+k^{\mathrm{P2Y_1}}_{-\mathrm{ADP}}\frac{\alpha K_{\mathrm{act}}}{\alpha K_{\mathrm{act}}+1}\right)\overline{[\mathrm{ADP}-\mathrm{P2Y_1}]}_S\\
&-\left(\beta k^{\mathrm{P2Y_1}}_{\mathrm{G_qGDP}}\frac{\alpha K_{\mathrm{act}}}{\alpha K_{\mathrm{act}}+1}\right)[\mathrm{G_qGDP}]\overline{[\mathrm{ADP}-\mathrm{P2Y_1}]}_S\\
&+\left(\frac{k^{\mathrm{P2Y_1}}_{-\mathrm{G_qGDP}}}{\delta\gamma}\frac{\alpha\beta\delta K_{\mathrm{act}}}{\alpha\beta\delta k_{\mathrm{act}}+1}\right)\overline{[\mathrm{ADP}-\mathrm{P2Y1}-\mathrm{G_qGDP}]}_S\\
&-\left(k^{\mathrm{P2Y_1}}_{\mathrm{G_qGTP}}\frac{\alpha K_{\mathrm{act}}}{\alpha K_{\mathrm{act}}+1}\right)[\mathrm{G_qGTP}]\overline{[\mathrm{ADP}-\mathrm{P2Y_1}]}_S\\
&+k^{\mathrm{P2Y_1}}_{-\mathrm{G_qGTP}}[\mathrm{ADP}-\mathrm{P2Y_1a}-\mathrm{G_qGTP}]_S\\
&-k^{\mathrm{P2Y_1}}_{\mathrm{phos}}\overline{[\mathrm{ADP}-\mathrm{P2Y_1}]}_S[\mathrm{PKCa}-\mathrm{Ca}-\mathrm{DAG}]+k^{\mathrm{P2Y_1}}_{\mathrm{dephos}}\overline{[\mathrm{ADP}-\mathrm{pP2Y_1}]}_S\end{aligned} \tag{100}$$

$$\begin{aligned}\frac{d}{dt}\overline{[\mathrm{P2Y_1}-\mathrm{G_qGDP}]}_S = &-\left(k^{\mathrm{P2Y_1}}_{\mathrm{ADP}}\frac{1}{\beta K_{\mathrm{act}}+1}+\alpha k^{\mathrm{P2Y_1}}_{\mathrm{ADP}}\frac{\beta K_{\mathrm{act}}}{\beta K_{\mathrm{act}}+1}\right)[\mathrm{ADP}]\overline{[\mathrm{P2Y_1}-\mathrm{G_qGDP}]}_S\\
&+\left(\frac{k^{\mathrm{P2Y_1}}_{-\mathrm{ADP}}}{\gamma}\frac{1}{\alpha\beta\delta K_{\mathrm{act}}+1}+\frac{k^{\mathrm{P2Y_1}}_{-\mathrm{ADP}}}{\delta\gamma}\frac{\alpha\beta\delta K_{\mathrm{act}}}{\alpha\beta\delta K_{\mathrm{act}}+1}\right)\overline{[\mathrm{ADP}-\mathrm{P2Y_1}-\mathrm{G_qGDP}]}_S\\
&+\left(\beta k^{\mathrm{P2Y_1}}_{\mathrm{G_qGDP}}\frac{K_{\mathrm{act}}}{K_{\mathrm{act}}+1}\right)[\mathrm{G_qGDP}]\overline{[\mathrm{P2Y_1}]}_S\\
&-\left(k^{\mathrm{P2Y_1}}_{-\mathrm{G_qGDP}}\frac{\beta K_{\mathrm{act}}}{\beta K_{\mathrm{act}}+1}\right)\overline{[\mathrm{P2Y_1}-\mathrm{G_qGDP}]}_S\\
&+k^{\mathrm{P2Y_1}}_{\mathrm{GDP}}[\mathrm{GDP}][\mathrm{P2Y_1a}-\mathrm{G_q}]_S-\left(k^{\mathrm{P2Y_1}}_{-\mathrm{GDP}}\frac{\beta K_{\mathrm{act}}}{\beta K_{\mathrm{act}}+1}\right)\overline{[\mathrm{P2Y_1}-\mathrm{G_qGDP}]}_S\end{aligned} \tag{101}$$

$$\begin{aligned}
\frac{d}{dt}\overline{[\mathrm{ADP}-\mathrm{P2Y_1}-\mathrm{G_qGDP}]}_S = &\left(k^{\mathrm{P2Y_1}}_{\mathrm{ADP}}\frac{1}{\beta K_{\mathrm{act}}+1}+k^{\mathrm{P2Y_1}}_{-\mathrm{ADP}}\frac{\alpha\beta K_{\mathrm{act}}}{\beta K_{\mathrm{act}}+1}\right)[\mathrm{ADP}]\overline{[\mathrm{P2Y_1}-\mathrm{G_qGDP}]}_S\\
&-\left(\frac{k^{\mathrm{P2Y_1}}_{-\mathrm{ADP}}}{\gamma}\frac{1}{\alpha\beta\delta K_{\mathrm{act}}+1}+\frac{k^{\mathrm{P2Y_1}}_{-\mathrm{ADP}}}{\delta\gamma}\frac{\alpha\beta\delta K_{\mathrm{act}}}{\alpha\beta\delta K_{\mathrm{act}}+1}\right)\overline{[\mathrm{ADP}-\mathrm{P2Y_1}-\mathrm{G_qGDP}]}_S\\
&\left(k^{\mathrm{P2Y_1}}_{\mathrm{G_qGDP}}\frac{\alpha\beta K_{\mathrm{act}}}{\alpha K_{\mathrm{act}}+1}\right)[\mathrm{G_qGDP}]\overline{[\mathrm{ADP}-\mathrm{P2Y_1}]}_S\\
&-\left(\frac{k^{\mathrm{P2Y_1}}_{-\mathrm{G_qGDP}}}{\delta\gamma}\frac{\alpha\beta\delta K_{\mathrm{act}}}{\alpha\beta\delta k_{\mathrm{act}}+1}\right)\overline{[\mathrm{ADP}-\mathrm{P2Y1}-\mathrm{G_qGDP}]}_S\\
&+k^{\mathrm{P2Y_1}}_{\mathrm{GDP}}[\mathrm{GDP}][\mathrm{ADP}-\mathrm{P2Y_1a}-\mathrm{G_q}]_S\\
&-\left(k^{\mathrm{P2Y_1}}_{-\mathrm{GDP}}\frac{\alpha\beta\delta K_{\mathrm{act}}}{\alpha\beta\delta K_{\mathrm{act}}+1}\right)\overline{[\mathrm{ADP}-\mathrm{P2Y1}-\mathrm{G_qGDP}]}_S\\
&-k^{\mathrm{P2Y_1}}_{\mathrm{phos}}\overline{[\mathrm{ADP}-\mathrm{P2Y_1}-\mathrm{G_qGDP}]}_S[\mathrm{PKCa}-\mathrm{Ca}-\mathrm{DAG}]\\
&+k^{\mathrm{P2Y_1}}_{\mathrm{dephos}}\overline{[\mathrm{ADP}-\mathrm{pP2Y_1}-\mathrm{G_qGDP}]}_S
\end{aligned} \tag{102}$$

$$\frac{d}{dt}\overline{[\mathrm{ADP}-\mathrm{pP2Y_1}]}_S = k^{\mathrm{P2Y_1}}_{\mathrm{phos}}\overline{[\mathrm{ADP}-\mathrm{P2Y_1}]}_S[\mathrm{PKCa}-\mathrm{Ca}-\mathrm{DAG}]-k^{\mathrm{P2Y_1}}_{\mathrm{dephos}}\overline{[\mathrm{ADP}-\mathrm{pP2Y_1}]}_S \tag{103}$$

$$\frac{d}{dt}[\mathrm{ADP}-\mathrm{pP2Y_1}-\mathrm{G_qGDP}]_S = k^{\mathrm{P2Y_1}}_{\mathrm{phos}}\overline{[\mathrm{ADP}-\mathrm{P2Y_1}-\mathrm{G_qGDP}]}_S[\mathrm{PKCa}-\mathrm{Ca}-\mathrm{DAG}]-k^{\mathrm{P2Y_1}}_{\mathrm{dephos}}\overline{[\mathrm{ADP}-\mathrm{pP2Y_1}-\mathrm{G_qGDP}]}_S \tag{104}$$

Which, after simplifying and dropping the bars and "a" from activated states, yields the equations in the earlier sections.

# References


[1] J. R. Atack, S. I. Rapoport, and C. L. Varley. Characterization of inositol monophosphatase in human cerebrospinal fluid. *Brain Research*, 613(2):305–308, 1993.

[2] U. S. Bhalla and R. Iyengar. Emergent properties of networks of biological signaling pathways. *Science*, 283(5400):381–387, 1999.

[3] A. A. Cook, W. Deng, J. Ren, R. Li, J. Sondek, and W. Bergmeier. Calcium-induced structural rearrangements release autoinhibition in the RAP-GEF CalDAG-GEFI. *J Biol Chem*, 293(22):8521–8529, 2018.

[4] R. S. E. Human platelets contain phospholipase c that hydrolyzes polyphosphoinositides. *PNAS*, 80(17):5417–5420, 1983.

[5] H. Kanoh, Y. Banno, M. Hirata, and Y. Nozawa. Partial purification and characterization of phosphatidylinositol kinases from human platelets. *Biochimica et Biophysica Acta (BBA) - Lipids and Lipid Metabolism*, 1046(2):120–126, 1990.

[6] M. J. Kelley and G. M. Carman. Purification and characterization of cdp-diacylglycerol synthase from saccharomyces cerevisiae. *Journal of Biological Chemistry*, 262(30):14563–14570, 1987.

[7] T. L. Kinzer-Ursem and J. J. Linderman. Both ligand- and cell-specific parameters control ligand agonism in a kinetic model of g protein–coupled receptor signaling. *PLoS Comput Biol*, 3(6):84–94, 2007.

[8] M. Matzaris, S. Jackson, K. Laxminarayan, C. Speed, and C. Mitchell. Identification and characterization of the phosphatidylinositol-(4, 5)-bisphosphate 5-phosphatase in human platelets. *Journal of Biological Chemistry*, 269(5):3397–3402, 1994.

[9] C. A. Mitchell, T. M. Connolly, and P. W. Majerus. Identification and isolation of a 75-kda inositol polyphosphate-5-phosphatase from human platelets. *Journal of Biological Chemistry*, 264(15):8873–8877, 1989.

[10] J. D. Moyer, O. Reizes, N. M. Dean, and N. Malinowski. D-myo-inositol (1,4)-bisphosphate 1-phosphatase. partial purification from rat liver and characterization. *Biochemical and Biophysical Research Communications*, 146(3):1018–1026, 1987.

[11] D. S. Paul, T. N. Blatt, W. J. Schug, E. G. Clark, T. Kawano, N. Mackman, S. Murcia, K. O. Poe, J. M. N. Mwiza, T. K. Harden, and et al. Loss of p2y1 receptor desensitization does not impact hemostasis or thrombosis despite increased platelet reactivity in vitro. *Journal of Thrombosis and Haemostasis*, 21(7):1891–1902, Jul 2023.

[12] J. E. Purvis, M. S. Chatterjee, L. F. Brass, and S. L. Diamond. A molecular signaling model of platelet phosphoinositide and calcium regulation during homeostasis and p2y1 activation. *Blood*, 112(10):4069–4079, 2008.

[13] J. Sneyd and J.-F. Dufour. A dynamic model of the type-2 inositol trisphosphate receptor. *Proc. Natl. Acad. Sci.*, 99:2398–2403, 2002.

[14] T. Urumow and O. Wieland. Purification and partial characterization of phosphatidylinositol-4-phosphate kinase from rat liver plasma membranes. further evidence for a stimulatory g-protein. *Biochimica et Biophysica Acta (BBA) - Molecular Cell Research*, 1052(1):152–158, 1990.

[15] L. A. Vargas, L. X. Miao, and A. F. Rosenthal. Inhibition of platelet phosphatidylinositol synthetase by an analog of cdp-diacylglycerol. *Biochimica et Biophysica Acta (BBA) - Lipids and Lipid Metabolism*, 796(2):123–128, 1984.

[16] J. Wissing, S. Heim, and K. G. Wagner. Diacylglycerol kinase from suspension cultured plant cells: purification and properties. *Plant Physiology*, 90(4):1546–1551, Aug 1989.

[17] M. Zeiler, M. Moser, and M. Mann. Copy number analysis of the murine platelet proteome spanning the complete abundance range. *Molecular and Cellular Proteomics*, 13(12):3435–3445, 2014.
